# Arene Activation by Calcium Hydride/Zinc Amide Equilibration

**DOI:** 10.1021/jacs.5c10735

**Published:** 2025-08-02

**Authors:** Kyle G. Pearce, Agustín Morales, Michael S. Hill, Mary F. Mahon, Claire L. McMullin

**Affiliations:** Department of Chemistry, 1555 University of Bath, Claverton Down, Bath BA2 7AY, U.K.

## Abstract

The β-diketiminato calcium hydride, [(BDI)­CaH]_2_ (BDI = HC­{(Me)­CNDipp}_2_, where Dipp = 2,6-*i*-Pr_2_C_6_H_3_), reacts with [Zn­{N­(SiMe_3_)_2_}_2_] and [Zn­(TMP)_2_] (TMP = 2,2,6,6-tetramethylpiperidide) to provide labile species and complex equilibria with a degree of commonality in ultimately providing the products of calcium to zinc β-diketiminate transmetalation. The silazide system provides the known [(BDI)­Ca­{N­(SiMe_3_)_2_}] and the heterobimetallic, [(BDI)­Ca­{N­(SiMe_3_)_2_}­(μ-H)­Zn]_2_, as a viable instrument for ligand transfer to yield [(BDI)­ZnH] as the final reaction product. In contrast, the TMP-derived system evidences an enhancement in Brønsted basicity, yielding [(BDI)­Zn­(C_6_H_5_)] by deprotonation of the benzene solvent. This process occurs via the heterobimetallic [(BDI)­Ca­(μ-N­{C­(CH_3_)_2_CH_2_}_2_CH)­(μ-H)­Zn­(μ-H)]_2_ and the previously unreported calcium amide [(BDI)­Ca­(TMP)]. The [(BDI)­Zn­(C_6_H_5_)] produced by this reaction may be exploited in biphenyl synthesis by a telescoped palladium-catalyzed cross-coupling with bromobenzene, which is unaffected by the presence of the other residual reaction products. This protocol has also been extended to the deprotonation of several further nonactivated arenes to yield the corresponding β-diketiminato arylzinc reagents with only mesitylene providing 3,5-dimethylbenzyl formation by C­(sp^3^)-H deprotonation. Although [(BDI)­Ca­(TMP)] reacts with both benzene and toluene, with the latter reaction providing the calcium benzyl, [(BDI)­Ca­(CH_2_C_6_H_5_)], and it is plausible that the latter species arises from initial C­(sp^2^)-H deprotonation and subsequent isomerization, density functional theory (DFT) calculations identify arene deprotonation to be kinetically and thermodynamically disfavored. The bimetallic derivative, [(BDI)­Ca­(μ-N­{C­(CH_3_)_2_­CH_2_}_2_CH)­(μ-H)­Zn­(μ-H)]_2_, cannot be definitively identified as the agent of arene deprotonation. We conclude, however, that the zinc arylation process requires the synergic cooperation of both metals.

## Graphical abstract

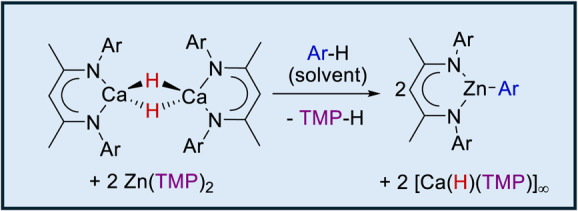

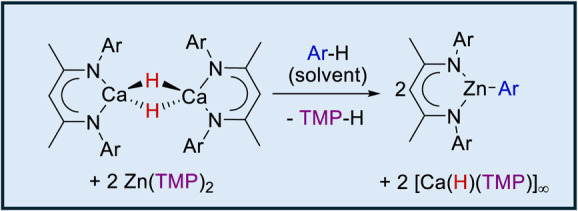

## Introduction

The deprotonation of carbon–hydrogen bonds provides a bedrock reaction in synthetic chemistry. Group 1 derivatives of so-called “utility amides” have, thus, long been indispensable as strong but non-nucleophilic Brønsted bases.^1,2^ Use of the 2,2,6,6-tetramethylpiperidide (TMP) anion (**I**, Scheme 1), for example, enables a suite of especially challenging deprotonation and bond activation processes. While the potential of systems derived from lithiated TMP has been appreciated for more than half a century,^3^ and has prompted rigorous study of their solution and solid-state structures,^4–8^ more recent reports have highlighted the complementary or advantageous reactivity achieved by extension to lithium’s heavier group 1 congeners, Na,^9–16^ K,^17,20^ Rb and Cs.^18^ Hevia and co-workers, for example, have shown that combinations of NaTMP, PMDETA (*N*,*N*,*N*′,*N*′,*N*′-pentamethyl­diethylene­triamine) and B­(O*i*-Pr)_3_ react with benzene, anisole or naphthalene to yield the relevant sodium arylboronates, presumably by boron ester interception of the incipient C_Ar_-Na bond.^12^

**Scheme 1:**
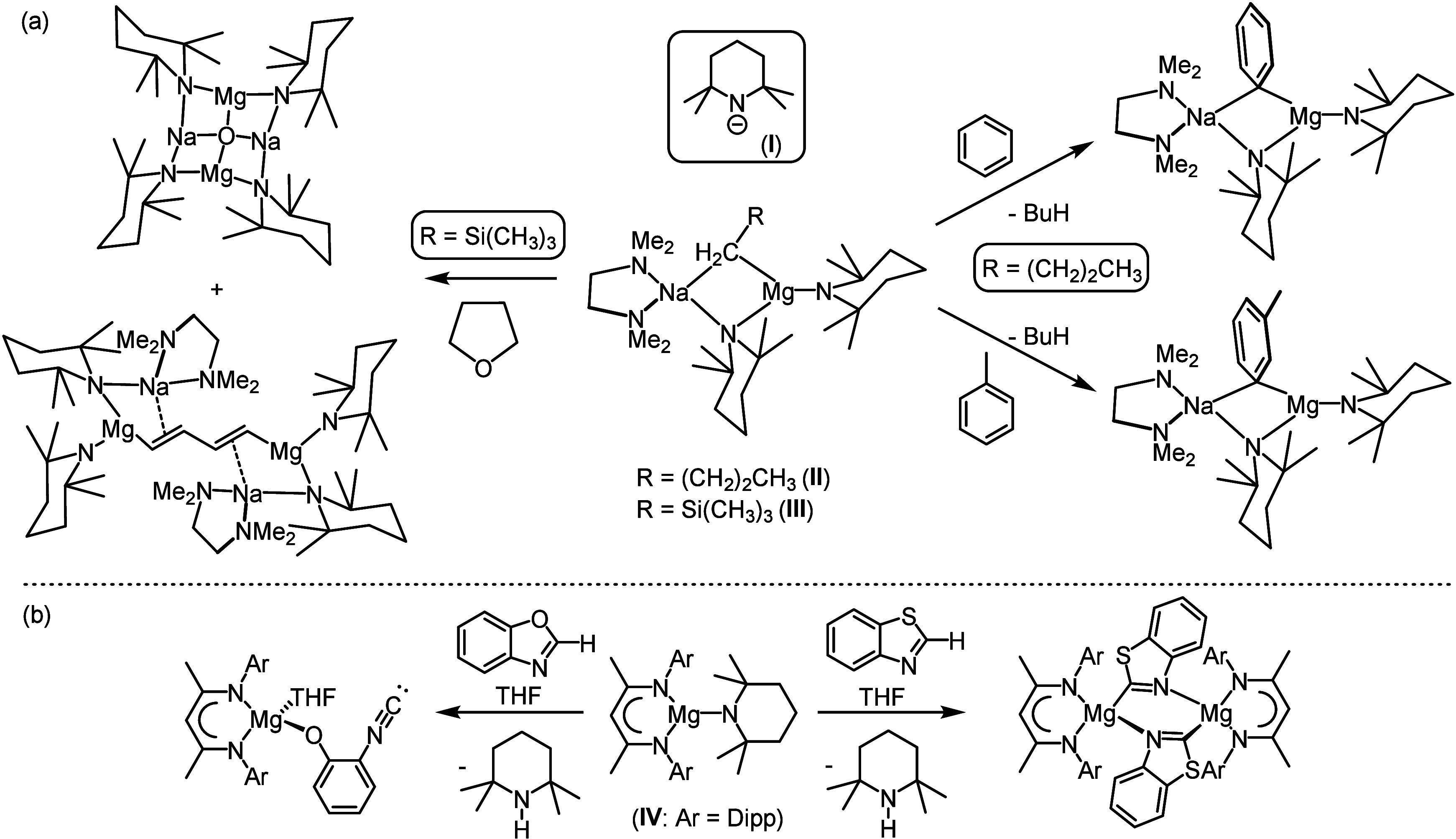
(a) Deprotonation of Benzene and *Meta* Deprotonation of Toluene by **II** and the Reactivity of of **III** toward THF^31^ and (b) Reactivity of **IV** with Benzothiazole and Benzoxazole^93^

The combination of LiTMP, NaTMP or KTMP with an organo-magnesium^19–44^ -zinc,^45–77^ -aluminum.^78–88^ or -gallium^83,84,89^ reagent has led to the development of potent bimetallic systems in which the metals combine in a spectacularly cooperative fashion to effect bond activation otherwise inaccessible to either monometallic component. Compound **II**, [(TMEDA)­Na­(μ-Bu)­(μ-TMP)­Mg­(TMP)], for example, affords its phenyl-bridged analogue when reacted with benzene^23^ and can effect the unprecedented *meta*-deprotonation of toluene (Scheme 1a).^27^ Although predated by Knochel’s “turbo” Hauser bases, [(TMP)­MgCl·LiCl], [(TMP)_2_Mg·LiCl] and [(TMP)_2_Zn·2MgCl_2_·2LiCl], for the selective metalation of more activated arenes and heteroarenes,^90–92^ the precise roles of both metals and the TMP anion throughout this reactivity remain somewhat ill-defined. The potency of such bimetallic combinations was, however, further demonstrated by the silylmethyl analogue of **II**, compound **III**, which induces the cleavage of two C–O and four C–H bonds of THF to yield both the “inverse crown”, [Na_2_Mg_2_(TMP)_4_(O)], and the dimagnesiated butadiene product, [{(TMEDA)­Na­(μ-TMP)}_2_­{1,4-(Mg­(TMP)}_2_­(C_4_H_4_)] (Scheme 1a).^31^

Beyond heterobimetallic systems, TMP reagents with magnesium as the sole constituent metal have also attracted scrutiny. In the early 1990s, for example, Eaton and co-workers demonstrated that magnesiation of methyl benzoate and cyclopropyl, cyclobutyl and even cubane carboxamides could be performed at 25 °C with [(TMP)_2_Mg] or [(TMP)­MgBr].^94–99^ Notably, however, while the use of such species has achieved the deprotonation of both borylated benzene and ferrocene molecules,^100,101^ no reactivity toward the less acidified C–H bonds (i.e., p*K*_a_ > 40) of wholly hydrocarbon arenes appears to have been documented.

Hevia and co-workers have more recently extended this research to the development of the β-diketiminate species, [(BDI)­Mg­(TMP)] (**IV**; BDI = HC­{(Me)­CNDipp}_2_, where Dipp = 2,6-*i*-Pr_2_C_6_H_3_).^93^ Compound **IV** effects the deprotonation of benzothiazole (p*K*_a_ *ca*. 27), *N*-methyl benzimidazole (p*K*_a_ *ca*. 32) and benzoxazole (p*K*_a_ *ca*. 25), albeit in the latter case with resultant C–O bond cleavage of the assumed C2-magnesiated-benzoxazolyl intermediate (Scheme 1b).^93,103^ Although this limited precedent evidences no appreciable advantage over other BDI-supported magnesium bases,^104^ the enhanced scope of reactivity provided by TMP derivatives of the heavier group 1 elements signposts the potential benefits of more focused development of tractable heavier alkaline earth tetramethylpiperidides.

Our own research has long exploited calcium hexamethyldisilazide derivatives such as [(BDI)­Ca­{N­(SiMe_3_)_2_}] (**V**) and [(BDI)­Ca­(THF)­{N­(SiMe_3_)_2_}] (**V·THF**) as workhorse reagents.^105–109^ Naïve consideration of the available p*K*_a_ data [(Me_3_Si)_2_N–H, *ca*, 25; TMP-H 37] indicates that progression to analogous TMP derivatives could represent an enhancement in basicity in the region of 10 orders of magnitude.^1,110,111^ With this observation in mind, it is, thus, surprising that examples of calcium TMP derivatives are limited to Westerhausen’s [Ca­(TMP)_2_(TMEDA)] and [Ca­(TMP­(TMEDA)­(μ-I)]_2_ (TMEDA = *N*,*N*,*N*′,*N*′-tetramethyl­ethylenediamine).^112,113^ While no reactivity of these compounds has been described, anecdotal evidence of attempts to synthesize base-free [(TMP)_2_Ca] hint at a tantalising potency derived from a combination of self- and solvent activation.^114^ Indeed, our own previous attempts to synthesize TMP variants of **V** or **V·THF** by an analogous salt metathesis route,^106,108^ were thwarted by the formation of an intractable mixture of products.^115^ This observation is also reminiscent of our and others’ attempts to access BDI organocalcium derivatives via organopotassium metathesis, which were frustrated by deprotonation of the BDI methyl substituents.^116,117^

In contrast to these complications, the base-free calcium hydride, [(BDI)­CaH]_2_ (**VI**), reacts with terminal alkenes to afford highly reactive σ-*n*-alkyl derivatives, [(BDI)­CaR]_2_.^118–121^ As a continuation of this theme, we have more recently observed that nucleophilic aryl derivatives may be accessed by reaction of **VI** with the appropriate Ar_2_Hg (Scheme 2).^122,123^ Circumventing the use of toxic organomercurials, treatment of **VI** with dialkylzinc compounds also results in the formation of [(BDI)­CaR]_2_.^124,125^ These alkylcalcium species, including the previously inaccessible methyl derivative,^124^ may be isolated in good yield after the initial formation of dimethyl­(hydrido)­zincate intermediates, but are liable to further transmetalation to [(BDI)­ZnR], which is irreversible due to the insoluble polymeric nature of [Ca­(H)­Me]_∞_ (Scheme 2).

**Scheme 2:**
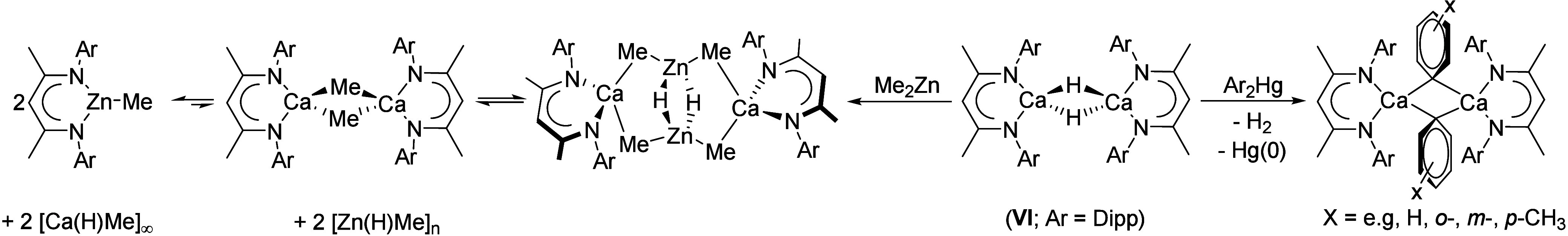
Reactivity of **VI** toward Mercury Aryls and Dimethylzinc

The apparent generality of this alkylzinc-to-calcium transmetalation led us to speculate that a similar zinc-based strategy could be applied to heteroatomic anions. In this contribution, therefore, we report the reactivity of **VI** toward the commercially available zinc amides, [Zn­{N­(SiMe_3_)_2_}_2_] and [Zn­(TMP)_2_], chemistry which presents a similar bimetallic pathway and proves to be a means to isolate [(BDI)­Ca­(TMP)]. While this latter compound is highly reactive, the selective deprotonation of nonacidified (i.e., p*K*a > *ca*. 40) arene C­(*sp* ^2^)-H bonds requires the retention of both the group 2 and group 12 centers. Although the origin of this reactivity cannot yet be established, these conditions allow the generation of useful [(BDI)­ZnAr] derivatives by *in situ* transmetalation of both the BDI and aryl anions from labile calcium-containing intermediates.

## Results and Discussion

### Zinc Amide/Calcium Hydride Transmetalation and Benzene C–H Metalation

The established utility of the calcium silazide reagents, **V** and **V·2THF**, led us to first assess the reactivity of compound **VI** and [Zn­{N­(SiMe_3_)_2_}_2_]. An initial reaction performed with a stoichiometric ratio of the two compounds proved inconclusive and provided a variety of unidentifiable BDI-containing products. Treatment of **VI** with two molar equivalents of the zinc amide, however, resulted in a single β-diketiminato product, identified at the first point of analysis by ^1^H NMR spectroscopy as the known zinc hydride, [(BDI)­ZnH] by its characteristic BDI γ-methine and hydridic singlet resonances at δ_H_ 5.02 and 4.39 ppm, respectively (Figure S4).^126^ This procedure evidently, therefore, results in facile room temperature calcium to zinc exchange of not only the amide and hydride functions but also the BDI ligands. Immediate cooling of an identical reaction mixture to −35 °C, however, afforded colorless single crystals of [(BDI)­Ca­{N­(SiMe_3_)_2_}­(μ-H)­Zn]_2_ (**1**), the structure of which was confirmed by X-ray diffraction analysis (Figure 1). Unlike the zincate complexes arising from the dialkylzinc reactions,^124,125^ compound **1** is better rationalized as a product of hydride-for-amide transmetalation in which two [(BDI)­Ca­{N­(SiMe_3_)_2_}] moieties are connected by a single {ZnH_2_} unit. The calcium and zinc centers are, thus, linked by a pair of μ_2_-bridging hydrides [Zn1–H1 1.603(18), Ca1–H1 2.25(2) Å]. Zn1 also interacts strongly with the γ-methine carbon of the BDI ligand backbones [Zn1–C2 2.2551(16) Å],^127^ albeit with a Zn–C bond length that is elongated in comparison to a typical Zn–C single bond [2.01 Å].^128–130^ The calcium atoms in **1** are situated ca. 1.498 Å above the least-squares plane defined by the BDI backbone and are oriented away from the Zn center. Despite the additional interaction between the calcium atoms and the bridging hydrides, the silazide ligands are bound with a Ca1–N3 bond [2.2739(13) Å] that is only marginally shorter than those reported for both [(BDI)­Ca­{N­(SiMe_3_)_2_}] [2.313(1) Å][Bibr ref106] and [(^DiPep^BDI)­Ca­{N­(SiMe_3_)_2_}] [2.2811(12) Å; ^DiPep^BDI = HC­{(Me)­CNDiPep}_2_; DiPep = 2,6-di-*iso-*pentylphenyl],^131^ in which the group 2 centers maintain distorted trigonal (N^*BDI*^)_2_ *N* ^*Si*^-coordination environments. From this structural perspective, therefore, compound **1** may be considered as a single molecular equivalent of {ZnH_2_} encapsulated by two molecules of [(BDI)­Ca­{N­(SiMe_3_)_2_}].

**Figure 1:**
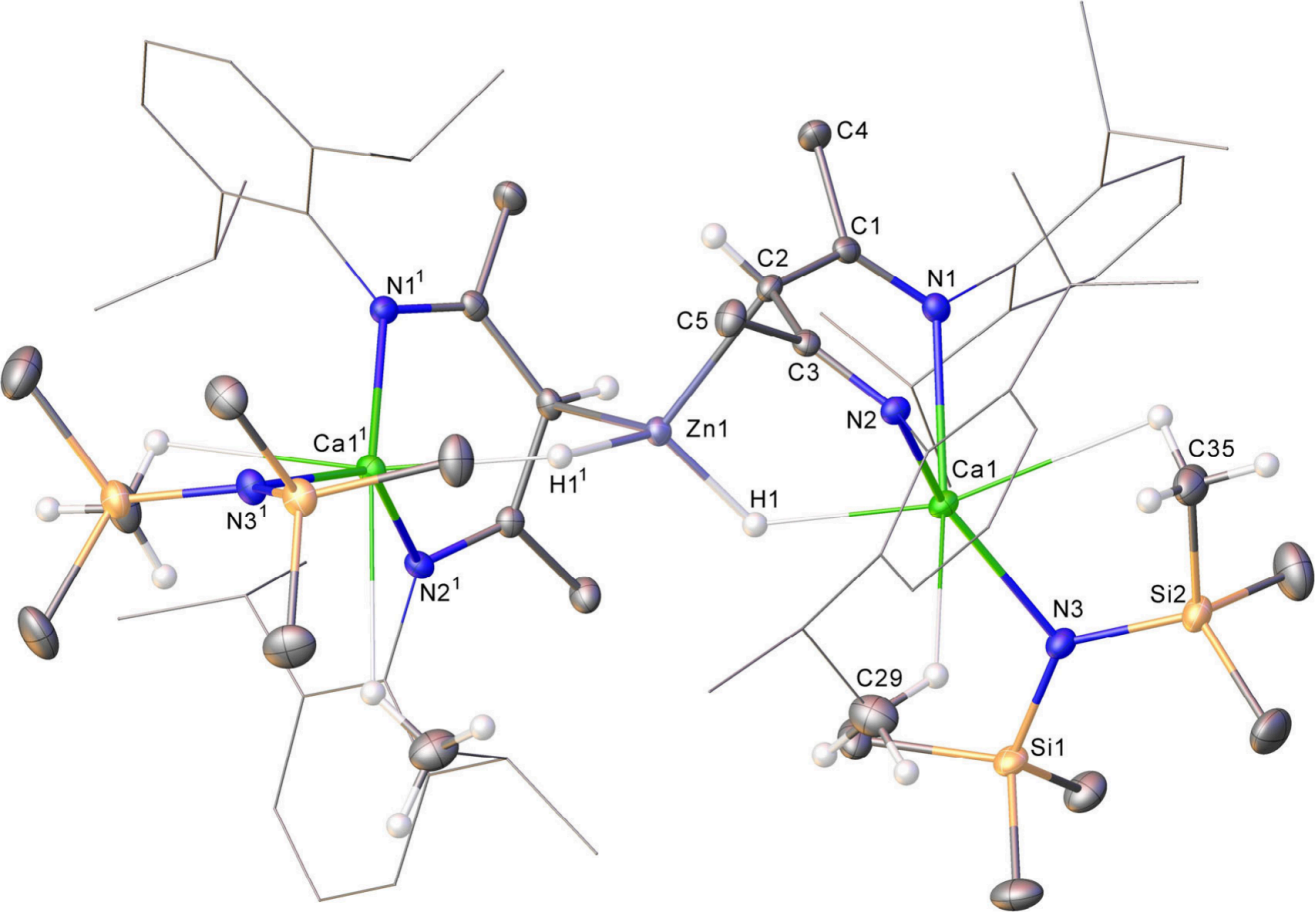
Molecular structure of compound **1** with displacement ellipsoids at 30%. For clarity, hydrogen atoms, apart from the bridging hydrides and those attached to C2, C29, C35, are omitted. Dipp groups are displayed as wireframe, also for visual ease. Selected bond lengths (Å) and angles (deg): Ca1–N1 2.3805(13), Ca1–N2 2.4078(14), Ca1–N3 2.2738(13), Ca1–Zn1 3.4290(6), Ca1–H1 2.25(2), Zn1–H1 1.603(18), Zn1–C2 2.2553(16). N1–Ca1–N2 78.85(4), N3–Ca1–N1 126.68(5), N3–Ca1–N2 140.77(5), Ca1–H1–Zn1 125.0(12), H1–Zn1–H1^1^ 122.2(13), C2–Zn1–C2^1^ 120.64(8). Symmetry operations to generate primed atoms: ^1^1–*x*, +*y*, 1/2–*z*.

Although the BDI γ-methine and Dipp *iso-*propyl methine resonances of **1** were clearly identifiable in the ^1^H NMR spectrum at δ_H_ 4.82 and 3.17 ppm, respectively, when redissolved in arene solvents, complete spectroscopic characterization was impeded by its immediate onward dismutation to [(BDI)­ZnH], even at −20 °C (Figure S6). The concurrent decrease in intensity of the signals assignable to **1**, however, occurred simultaneously with the immediate emergence of a steady state quantity of [(BDI)­Ca­{N­(SiMe_3_)_2_}] (**V**; δ_H_ 4.77, 3.12, and 0.06 ppm),^106^ alongside the growth of signals of [(BDI)­ZnH] as the ultimate β-diketiminate-containing product (Figures S1–S3). The complete process giving rise to these observations is summarized in Scheme 3.

**Scheme 3:**
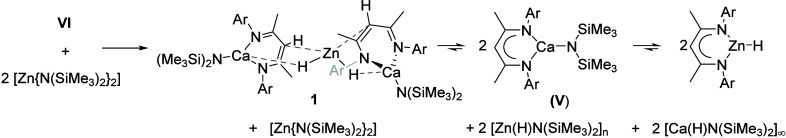
Reaction Pathway Arising from the Reaction of [Zn­{N­(SiMe_3_)_2_}_2_] and [(BDI)­CaH]_2_ (**VI**)

Encouraged by the isolation of compound **1** and the identification of the known species [(BDI)­Ca­{N­(SiMe_3_)_2_}], we sought to extend this hydride-for-amide exchange to the synthesis of its previously unreported 2,2,6,6-tetramethylpiperidide analogue. Two equivalents of [Zn­(TMP)_2_] were, thus, reacted with **VI** in C_6_D_6_ at room temperature (Scheme 4). A single BDI-containing species (**2**-***d***) was readily characterized through the emergence of a BDI γ-methine singlet at 5.06 ppm in the ^1^H NMR spectrum after 16 h, which appeared alongside a series of aliphatic C–H resonances consistent with the presence of TMP-D.^132^ Crystallization and single crystal X-ray diffraction analysis, confirmed **2-** ***d*** to be [(BDI)­Zn­(C_6_D_5_)], the deuterated analogue of the known β-diketiminato phenylzinc complex.^133^ Repetition of this reaction in protio-benzene provided a ^1^H NMR spectrum comprising not only an identical γ-BDI methine singlet (δ_H_ 5.06 ppm) but also the phenyl doublet and multiplet resonances (δ_H_ 6.57, 6.96 ppm, respectively) associated with the aromatic C_6_H_5_ unit of [(BDI)­Zn­(C_6_H_5_)] (**2**, Scheme 4). Notably, no evidence of H_2_ or H-D formation could be observed in the respective reactions in C_6_H_6_ or C_6_D_6_.

**Scheme 4:**
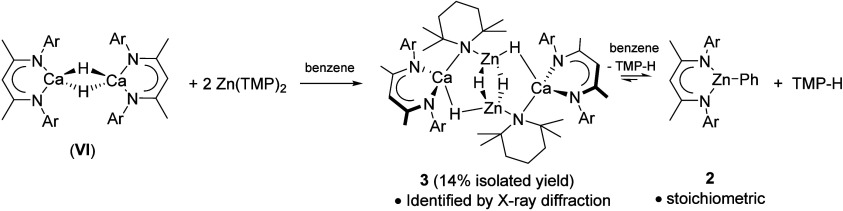
Formation of Compound **3** Leading to Compound **2** by Benzene Deprotonation^a^

a Stoichiometry and other potential products not shown.

In an attempt to isolate and identify the assumed heterobimetallic pretransmetalation intermediate formed prior to the generation of **2**, a further reaction between **VI** and [Zn­(TMP)_2_] was performed and immediately stored at low temperature. While this procedure provided a small number of colorless crystals (14% isolated yield) of a new species (**3**), its solution characterization was thwarted by both the small amount of material isolated and its continued transformation, even when the temperature was maintained at –40 °C. Irrespective of these issues, single-crystal X-ray diffraction analysis elucidated the solid-state structure of **3** as the calcium dihydrido­(amido)­zincate, [(BDI)­Ca­(μ-N­{C­(CH_3_)_2_CH_2_}_2_CH)­(μ-H)­Zn­(μ-H)]_2_ (Figure 2, Scheme 4).

**Figure 2:**
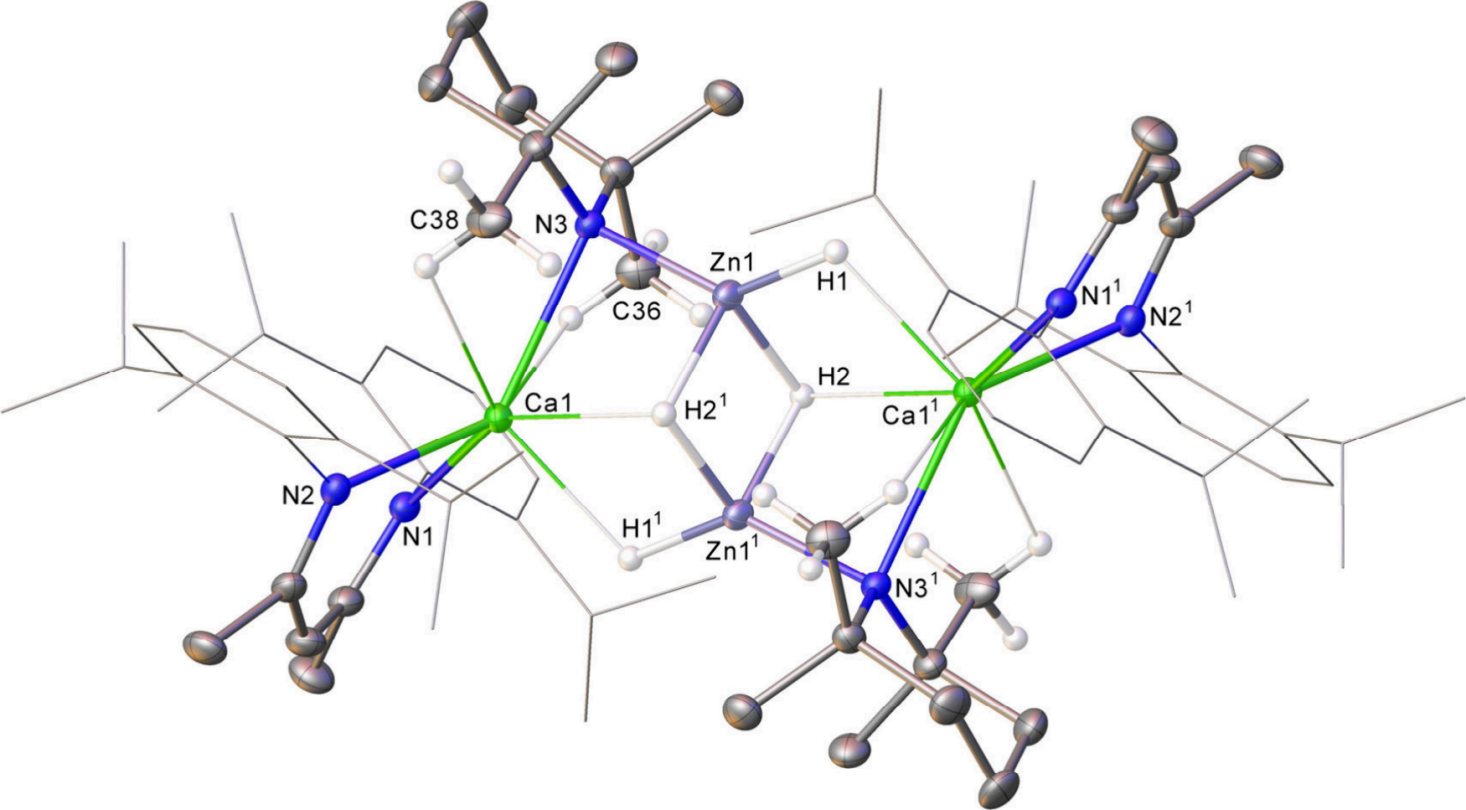
Molecular structure of compound **3** with displacement ellipsoids at 30%. For clarity, hydrogen atoms, apart from the bridging hydrides and those attached to C36, C38, C36^1^ and C38^1^, are omitted, as are the solvent molecules. Dipp groups are displayed as wireframe, also for visual ease. Selected bond lengths (Å): Ca1–N1 2.3963(15), Ca1–N2 2.3997(15), Ca1–N3 2.5271(14), Ca1–Zn1 3.1878(5), Ca1–Zn1^1^ 3.1242(5), Ca1–H1^1^ 2.28(3), Ca1–H2^1^ 2.57(2) Ca1–H2 3.60(2), Zn1–H1 1.59(3), Zn1–H2^1^ 1.79(2), Zn1–H2 1.79(2), Zn1–N3 2.0043(14). Zn1–Zn1^1^ 2.6015(6). Symmetry operations to generate primed atoms: ^1^1–*x*, 1–*y*, 1–*z*; ^2^1–*x*, 1–*y*, −*z*; ^3^2–*x*, 1–*y*, 1–*z*.

Although the centrosymmetric calcium zincate dimer (**3**) is again propagated by Ca-(μ-H)-Zn bridging interactions, its constitution differs significantly from both compound **1** and the previously described alkyl­(hydrido)­zincate species (Scheme 2).^124,125^ Compound **3** may be considered as a β-diketiminato calcium derivative of a tetramethyl­piperidinido­(dihydrido)­zincate, the anions of which dimerize via Zn1-(μ_2_-H1)_2_-Zn1^1^ bridging interactions. Although compound **3** could also be regarded as a molecular {ZnH_2_}_2_ dimer adducted by two equivalents of [(BDI)­Ca­(TMP)], this interpretation is not borne out by the most relevant metric data. Each zinc is connected to Ca1 via μ_2_-interactions with both H2 and the N3/N3^1^ atoms of a TMP anion. The resultant Zn1–N3 distance [2.0043(14) Å] is comparable to precedented zinc species in which the TMP anion adopts a μ_2_-bridging role between two dissimilar metals, for example [(TMEDA)­Na­(μ-R)­(μ-TMP)­Zn­(^t^Bu)] (R = ^t^Bu, Ph, pyrrolyl) (ca. 2.0 Å).^48,67^ Although no direct comparators exist for calcium-to-TMP bridging interactions, the Ca1–N3 distance [2.5271(14) Å] is significantly elongated in comparison to the terminal Ca–N^*TMP*^ bond distances described by Westerhausen and co-workers for [Ca­(TMP)_2_(TMEDA)] [2.275(2) Å] and [Ca­(TMP)­(TMEDA)­(μ-I)]_2_ [2.243(2) Å].^112,113^

### Calcium-Initiated Arene C–H Zincation

Equipped with these insights, we turned our attention to the potential generality of the arene activation manifested by the identification of compound **2**. Crimmin and co-workers have previously reported that complexes of the form [(BDI)­Zn–Ar] are effective sources of aryl nucleophile in Negishi cross coupling.^133^ Although the reactive species in this earlier work were accessed by palladium-catalyzed dehydrocoupling of [(BDI)­ZnH] and an arene C­(*sp* ^2^)-H bond, the reaction scope was largely limited to the acidic 2-positions of furan (p*K*_*a*_ *ca*. 35)^102^ and thiophene (p*K*_a_ *ca*. 33) derivatives, the 3-position of *N*-sulfonated indole (p*K*_a_ *ca*. 41) and polyfluorinated arenes, metalation of which declined significantly with decreasing levels of fluorination (Scheme 5). While this process was also reported to be applicable to the zincation of benzene (p*K*_a_ 43), the reaction was notably sluggish (ca. 60% conversion to **2** after days at 100 °C, Scheme 5) and required the application of a static vacuum to remove the H_2_ byproduct. In a similar vein, a subsequent study by Ingleson and co-workers demonstrated the zincation of a similar range of heteroarenes (i.e., furans, thiophenes and indoles) with a combination of [(BDI)­ZnH] in the presence of catalytic (10 mol %, 60 °C, 24 h) [Et_3_NH]^+^­[B­{3,5-C_6_H_3_(CF_3_)_2_}_4_]^−^.^134^

**Scheme 5:**
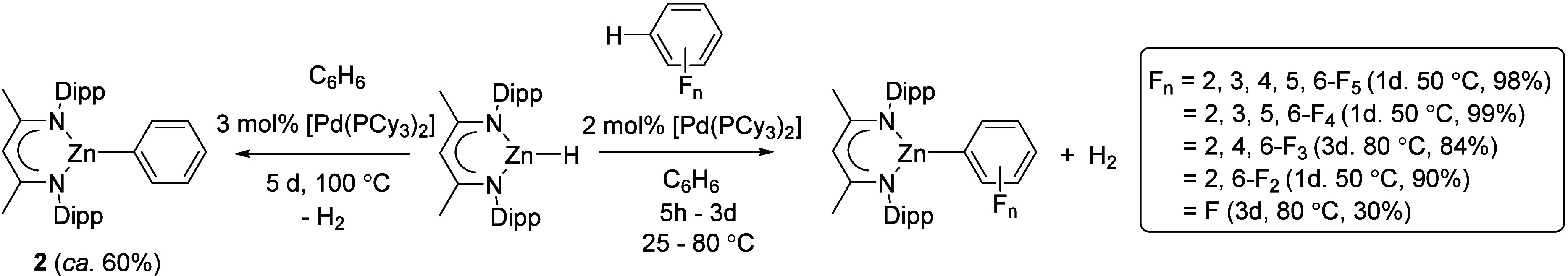
Crimmin and Co-Workers’ Pd-Catalyzed Zincation of Fluoroarenes and the Extension of This Protocol to Benzene^133^

These recent precedents, thus, bear significant commonality with the current work in remaining dependent on the use of stoichiometric equivalents of both the β-diketiminate and zinc constituents of the reaction. The current more ready formation of compound **2** (Scheme 4), however, suggests a greater potential facility for C­(*sp* ^2^)-H deprotonation may be achieved through the combination of **VI** and [Zn­(TMP)_2_], which, although noncatalytic, is derived from highly earth-abundant calcium and a commercially available zinc amide. To assess the broader potential of this system for the metalation of wholly hydrocarbon arene molecules, therefore, a series of further reactions were performed under conditions relevant to the synthesis of compound **2**.

The reaction of compound **VI** and [Zn­(TMP)_2_] was again first performed in benzene but, for synthetic expedience, at the mildly elevated temperature of 60 °C (Table 1, entry 1). This adjustment in the reaction conditions again resulted in the selective formation of compound **2** but now provided an improved isolated and crystallized yield of 89% (vs. 67% at room temperature). Subsequent experiments, therefore, replicated these conditions but employed toluene, mesitylene or 1,3-di-*tert*-butylbenzene as the arene solvent in place of benzene.

**Table 1:**
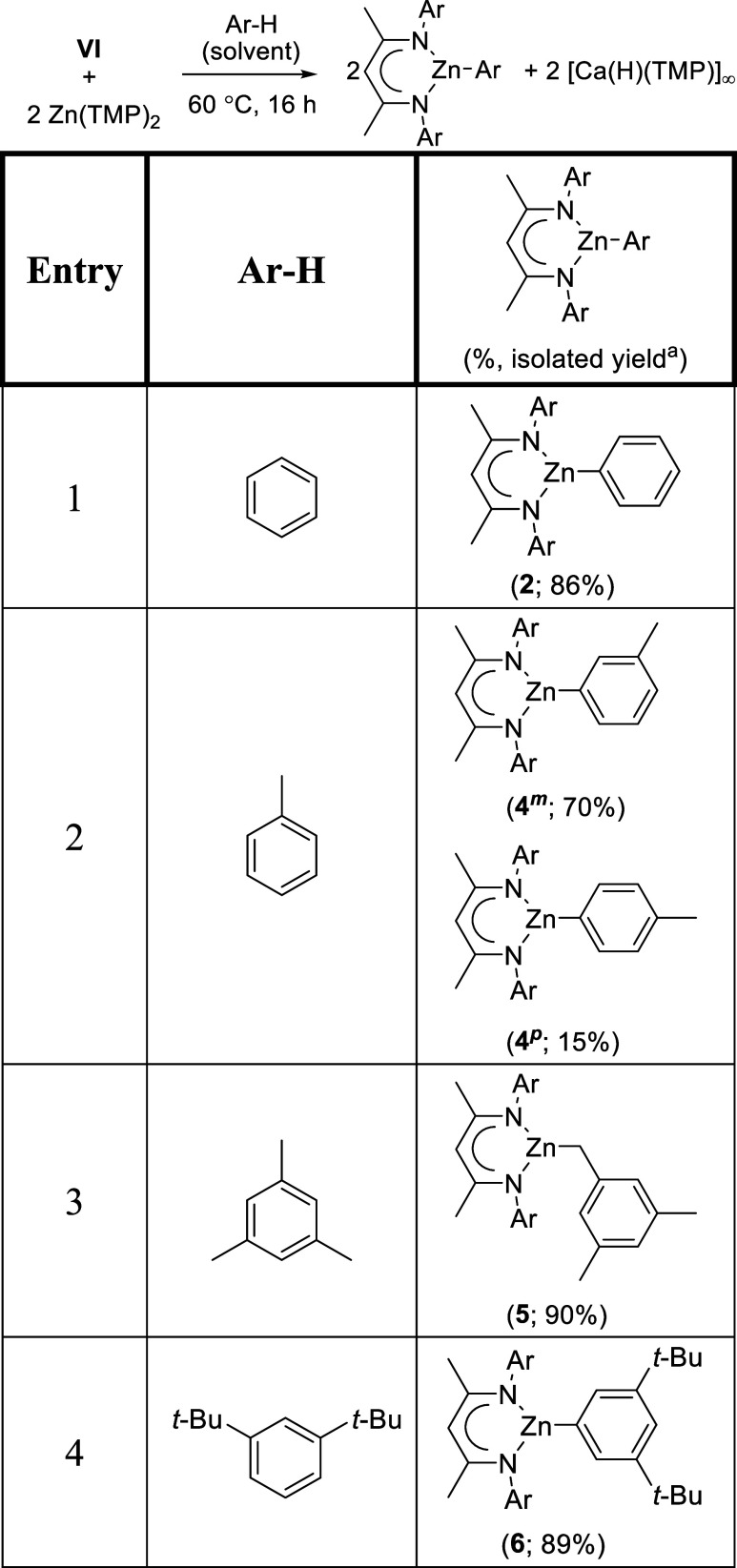
Zincation of Arene C–H Bonds Induced by a Combination of **VI** and [Zn­(TMP)_2_]­^a^

a Conversions deduced to be stoichiometric relative to **VI** by ^1^H NMR spectroscopy.

The results of this assay confirmed the generality of the one pot conversion to a BDI-supported organozinc species derived from C–H activation/deprotonation of the relevant arene solvent. The toluene-based reaction provided two isomeric products (Table 1, entry 2) derived by metalation of a methine C­(*sp* ^2^)-H bond, the 3-methylphenyl derivative (**4** ^***m***^, 70%) and small but significant quantities of its 4-methylphenyl isomer (**4** ^***p***^, 15%). This observation is reminiscent of, for example, Mulvey and co-workers’ elaboration of toluene *meta*-magnesiation by **II** (Scheme 1a), which also occurs in contradiction to the thermodynamic expectation of benzyl anion formation.^27^ The predominant generation of **4** ^***m***^ by the current system, and the absence of any indication of its 2-methylphenyl analogue (**4** ^***o***^) is, thus, similarly consistent with a reaction operating under kinetic control.

In contrast to this outcome, a reaction performed in mesitylene provided exclusive aliphatic C­(*sp* ^3^)-H bond activation and the benzylzinc derivative (**5**, Table 1, entry 3). Although we have no definitive rationale for this result, the formation of compound **5** is reminiscent of earlier observations of C–H activation/deprotonation of this substituted arene, where a statistical bias toward reaction at one of the nine more sterically accessible methyl hydrogens overrides any potential for activation of the three arene methine C–H bonds.^135,136^ Steric considerations also dictate that 1,3-di-*tert*-butylbenzene, while comprising three contiguous C–H methine bonds, undergoes exclusive zincation to provide compound **6** (89%, Table 1, entry 4) at the most kinetically accessible, albeit also most thermodynamically favored, 4-position of the arene ring.

Compounds **2** and **4** ^***m,p***^–**6** were isolated and fully characterized by ^1^H and ^13^C NMR spectroscopy and, for **4** ^***m,p***^–**6**, by single crystal X-ray diffraction analysis (Figure 3 and the Supporting Information). Although the resemblance of compounds **4** ^***m***^, **4** ^***p***^ and **6** to known β-diketiminate-supported zinc aryl complexes obviates any necessary comment,^133,137^ the ultimate utility of this arene zincation protocol lies in its significant potential in onward synthesis.^138^ In this regard, Crimmin and co-workers have shown that perfluoroarene species, [(BDI)­Zn­(C_6_F_5_)], when generated *in situ* and in the presence of other reaction products, may be applied in Negishi cross-coupling with C_6_H_5_Br in the presence of ca. 3 mol % [Pd­(PCy_3_)_2_].^133^ In an initial demonstration of the applicability of the current protocol to the elaboration of less activated arenes, therefore, the synthesis of compound **2** was repeated under the conditions summarized by Table 1, entry 1. Removal of volatiles at this juncture followed by addition of a stoichiometric equivalent of C_6_H_5_Br in the presence of 5 mol % [Pd­(PCy_3_)_2_] in benzene under the reaction conditions applied by Crimmin and co-workers (60 °C, 24 h) provided similarly quantitative biphenyl formation. Notably, this reaction was performed in the presence of the various implied but insoluble calcium- and hydride-containing reaction byproducts and, albeit of a very preliminary nature, confirms the potential of this protocol for the telescoped incorporation of unactivated arenes in Negishi-type cross-coupling,

**Figure 3:**
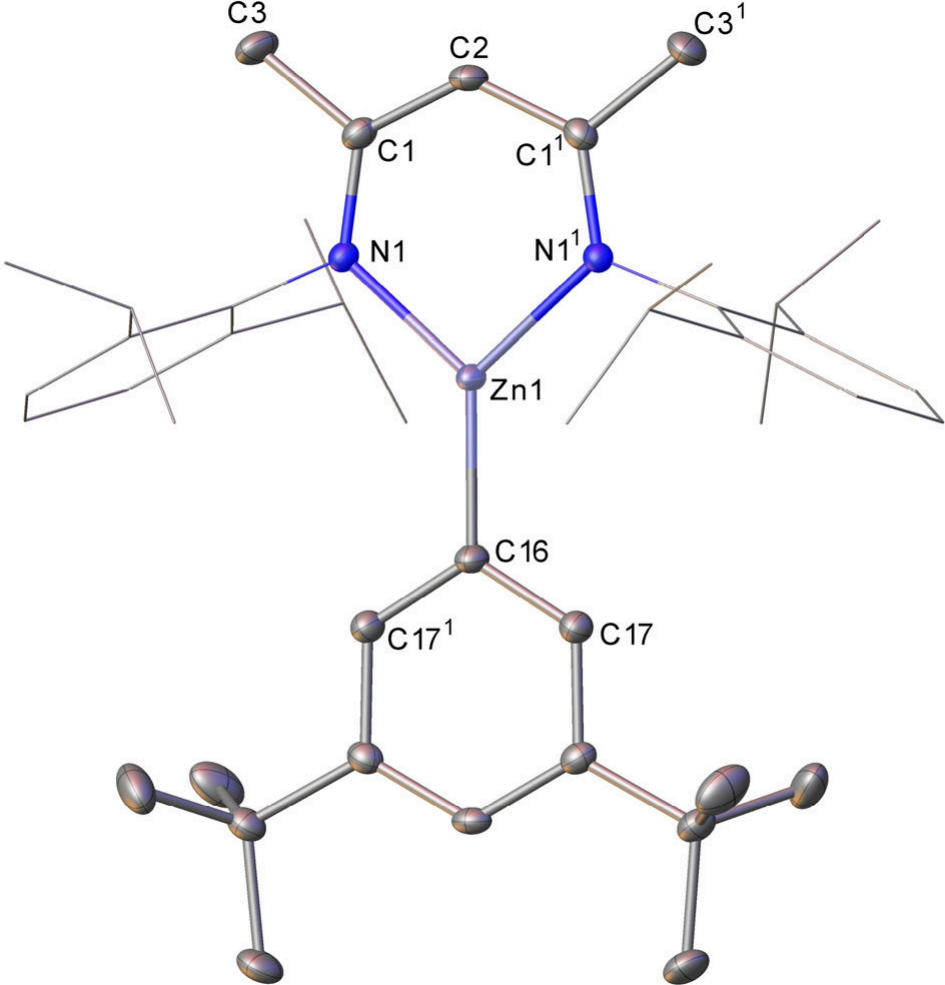
Molecular structure of **6**, displacement ellipsoids at 30%. For clarity, hydrogen atoms are omitted and the Dipp groups are displayed as wireframe. Selected bond lengths (Å) and angles (deg): Zn1–N1 1.9429(14), Zn1–C16 1.936(2), N1–Zn1–N1^1^ 97.09(8), C16–Zn1–N1^1^ 131.45(4), C16–Zn1–N1 131.45(4). Symmetry operations to generate primed atoms: ^1^1+*x*, 1/2–*y*, +*z*.

### Isolation of [(BDI)­Ca­(TMP)]

The equal number of constituent zinc atoms and {(BDI)­Ca} units presented by the structure of compound **3** are consistent with expectation provided by the reaction stoichiometry. The incorporation of two hydride ligands per zinc and calcium center, however, contradicts the applied reaction stoichiometry and implies the concurrent generation of further TMP-derived calcium and/or zinc products, which could conceivably play key roles in the arene zincation outlined above. In a further attempt to identify such species, therefore, the reaction between **VI** and [Zn­(TMP)_2_] was repeated in hexane at −35 °C. This adjustment to the reaction conditions readily afforded a crop of pale-yellow crystals of a further product (**7**) in moderate but reproducible yield (29%). A promptly acquired ^1^H NMR spectrum of an isolated sample of **7** in *d*_8_-toluene, necessarily recorded at 233 K to limit reaction with the solvent (*vide infra*), presented a characteristic BDI γ-methine singlet at 4.87 ppm, which integrated with terminal-CH_3_ (δ_H_ 0.96 ppm) and cyclic methylene (δ_H_ 1.83, 1.27 ppm) TMP resonances in a relative 2:4:12 ratio. The implied formulation of compound **7** as [(BDI)­Ca­(TMP)] was subsequently confirmed in the solid state by single-crystal X-ray diffraction analysis (Figure 4).

**Figure 4:**
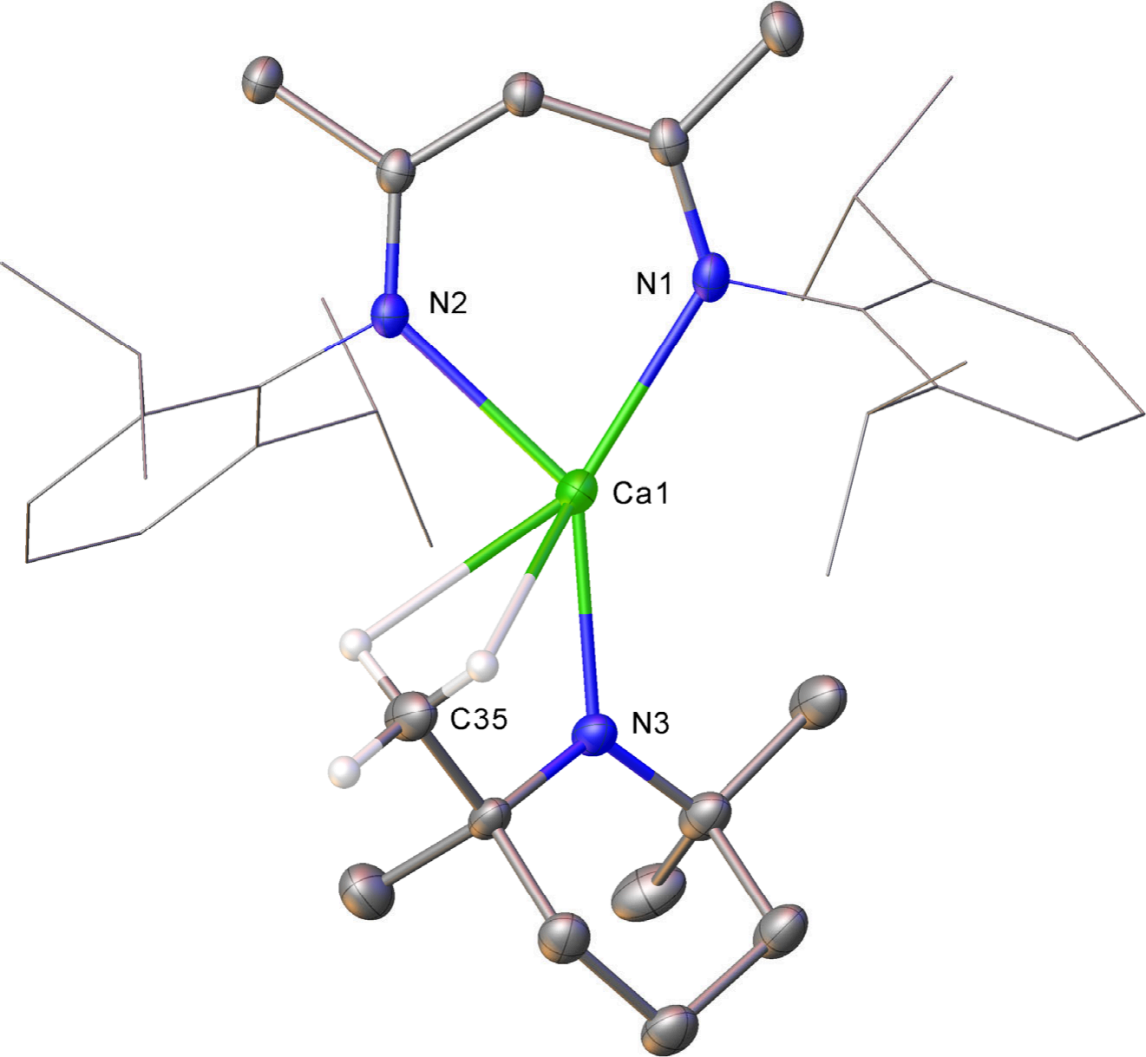
Molecular structure of **7**, displacement ellipsoids at 30%. For clarity, hydrogen atoms, apart from those attached to C35, are omitted and the Dipp groups are displayed as wireframe. Selected bond lengths (Å) and angles (deg): Ca1–N1 2.3347(11), Ca1–N2 2.3579(10), Ca1–N3 2.2372(19), Ca1–C35 2.945(3). N1–Ca1–N2 78.63(4), N3–Ca1–N1 143.90(7), N3–Ca1–N2 137.44(7), N3–Ca1–N1 143.90(7).

In a similar manner to its previously reported lighter congener, [(BDI)­Mg­(TMP)] (**IV**),^93^ compound **7** is a three-coordinate monomeric complex in which the alkaline earth (Ca1) and TMP nitrogen (N3) atoms are respectively situated ca. 0.148 and 0.277 Å above the plane defined by the β-diketiminate chelate. In contrast to the relevant distance presented by the bridging TMP anions of **3** [2.571(14) Å], the calcium-TMP interaction in **7** [Ca1–N3 2.2372(19) Å] is more closely comparable with those reported for the similarly terminal Ca–N^*TMP*^ bonds described by Westerhausen for [(TMEDA)­Ca­(TMP)_2_] [2.275(2) Å] and [Ca­(TMP)­(TMEDA)­(μ-I)]_2_ [2.243(2) Å].^112,113^

The identification of **7** provides a further plausible agent of solvent activation leading to [(BDI)­Zn­(C_6_H_5_)] (**2**). Although a variety of zincate systems, for example Kondo’s [(TMP)*t*-Bu_2_ZnLi] and Hevia’s [{Ph_2_Si­(NDipp)_2_­Zn­(TMP)}^−^{K­(THF)_6_}^+^] and [Zn­(TMP)_2_]/KO*t*Bu systems, have enabled the regioselective zincation of arenes equipped with electron-withdrawing directing groups or fluoroarenes,^72,74,77^ no reactivity of this class of species toward nonactivated arenes appears to have been described. Similarly, while [(BDI)­Zn­(TMP)], to the best our knowledge, has not yet been reported, we could observe no evidence of reaction throughout monitoring of a sample of [Zn­(TMP)_2_] stored in benzene for 3 days. Prompted by the implication that monometallic **7** may be the reagent responsible for solvent metalation prior to the formation of **2** or **4** ^***m,p***^–**6**, therefore, an isolated sample of **7** was dissolved in benzene at ambient temperature and monitored by ^1^H NMR spectroscopy. Although an intractable mixture of species was apparent after only 1 h (Figure S15), our previous observations of the marked solution instability of dimeric [(BDI)­Ca­(C_6_H_5_)]_2_,^122^ suggest that any putative phenylcalcium product of benzene deprotonation by **7** is also likely to be a highly labile species, the identity of which may only be inferred by its facile transmetalation to zinc to form **2**.

To further test this hypothesis, a sample of **7** dissolved in toluene led to the rapid deposition of yellow needle-like crystals of compound **8**. While isolated crystals of **8** were subsequently insoluble in noncoordinating hydrocarbon solvents, their prompt analysis in *d*_8_-THF by ^1^H NMR spectroscopy (before complete decomposition) identified a series of benzylic resonances (δ_H_ 6.41, 5.95, 5.75 ppm; Figure 5a), which integrated in a 2:2:1 ratio against the BDI γ-methine (δ_H_ 4.73 ppm, 1H) and Dipp-methine (δ_H_ 3.24 ppm, 4H; Figure S16) signals of the β-diketiminate ligand. The implied constitution of **8** as [(BDI)­Ca­{CH_2_C_6_H_5_}] was further supported by microanalysis and ultimately confirmed by X-ray diffraction analysis (Figure 5b). Although the rapidly deposited crystals of **8** were invariably very small and weakly diffracting, recourse to a synchrotron radiation source provided data, which, although of insufficient quality to allow anisotropic refinement, were adequate to confirm connectivity. Compound **8** clearly describes a tetrameric aggregate with each calcium equipped with a β-diketiminate ligand and a σ-bonded benzylic substituent, which also engages via a sequence of η^6^-π interactions with an adjacent calcium center to propagate the oligomeric structure.

**Figure 5:**
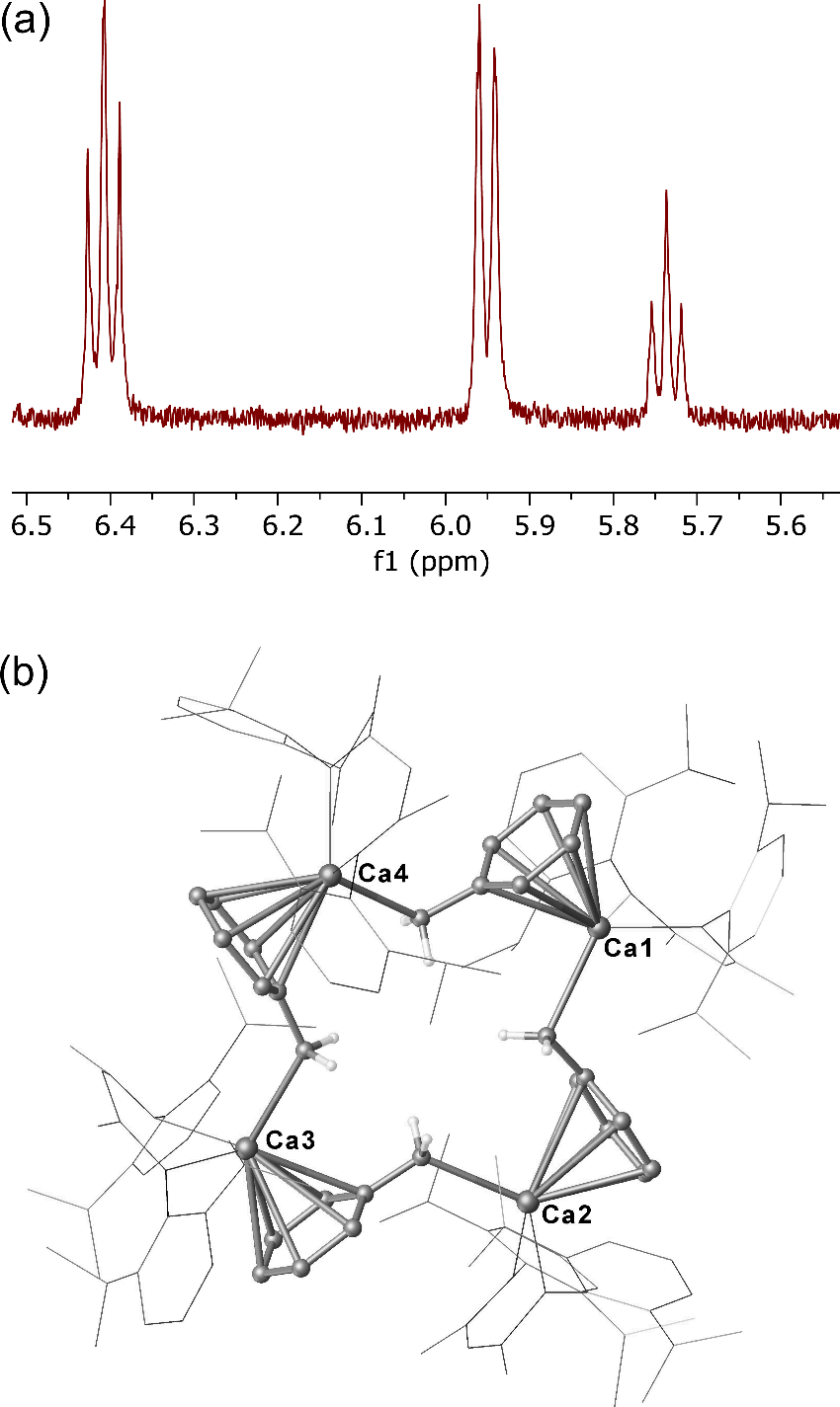
(a) Benzylic region from the ^1^H NMR spectrum for [(BDI)­Ca­{CH_2_C_6_H_5_}] (**8**) (*d*_8_-THF, 298 K, 400.13 MHz). (b) Ball and stick representation of the tetrameric connectivity of compound **8** with BDI ligands shown as wireframe for clarity.

### DFT Study

While the experiments summarized in Table 1 provide definitive evidence for the viability of arene zincation provided by **VI** and [Zn­(TMP)_2_], our previous observations of **VI** and **VI·2THF** as reagents in a wide variety of processes have emphasized the complexity of equilibria exemplified by Schemes 3 and 4, and a common feature that unidentifiable species often provide the origin of observed catalysis.^105,139–142^ Although compounds **3** and **7** are undoubted components in the reaction mixtures and their significance cannot be discounted, their relevance to the generation of **2** and **4** ^***m,p***^–**6**, and, more specifically, their potential roles during arene deprotonation are less readily ascribed. The heterobimetallic complexity of compound **3** led us to scrutinize the monomolecular calcium TMP derivative (**7**) as a more tractable system for theoretical consideration. While the reactivity of **7** toward toluene clearly demonstrates an exceptional basicity in comparison to its silazido analogue (**V**), the benzylic constitution of **8** presents a contradictory outcome with regard to the formation of the tolylzinc derivatives, **4** ^***m***^ and **4** ^***p***^.

The reactivity of **7** toward the various C­(*sp* ^2^)-H bonds of benzene and toluene was, thus, assessed by density functional theory (DFT) calculations. These results are summarized in Table 2 with free energies quoted relative to a zero provided by the initial complex of **7** and the relevant associated arene, [(BDI)­Ca­(TMP)·(Arene)], (henceforth denoted as **A** when Arene = C_6_H_6_ or **C** when Arene = C_7_H_8_). Similarly accessible transition states (Δ*G* ^‡^ = *ca*. 20 kcal mol^–1^) representing the direct deprotonation of benzene to provide **B**, [(BDI)­Ca­(C_6_H_5_)·TMPH], and both the 3- and 4-methine positions of toluene yielding **D** ^***m***^, [(BDI)­Ca­(3-CH_3_C_6_H_4_)·TMPH], and **D** ^***p***^, [(BDI)­Ca­(4-CH_3_C_6_H_4_)·TMPH], were successfully characterized. In contrast, *ortho*-activation of toluene to provide **D** ^***o***^, [(BDI)­Ca­(2-CH_3_C_6_H_4_)·TMPH] (i.e., potentially leading to the unobserved 2-methylphenyl aryl zinc isomer) was identified to be some 5 kcal mol^–1^ more energetically demanding. Although the relative kinetic viability of these processes is, thus, consistent with the constitutions of the experimentally observed phenyl- and tolylzinc species, the formation of all four arylcalcium products was calculated to be endergonic (Δ*G* = *ca*. +14 kcal mol^–1^). This clear contradiction to the role of **7** as the sole agent of arene activation was underscored by a further assessment of toluene methyl deprotonation to provide **E**, [(BDI)­Ca­(CH_2_C_6_H_4_)·TMPH]. Formation of this species, observed experimentally as **8**, was calculated to be contrastingly exergonic (Δ*G* = −8.2 kcal mol^–1^) with a barrier height that is, at worst, competitive (Δ*G* ^‡^ = 18 kcal mol^–1^) with arene proton removal. These theoretical results, therefore, strongly infer that the experimentally identified benzylcalcium species (**8**) is not relevant to the formation of **4** ^***m***^ and **4** ^***p***^ and, by extension, that the zincated products listed in Table 1 do not arise through the intermediacy of compound **7**.

**Table 2:**
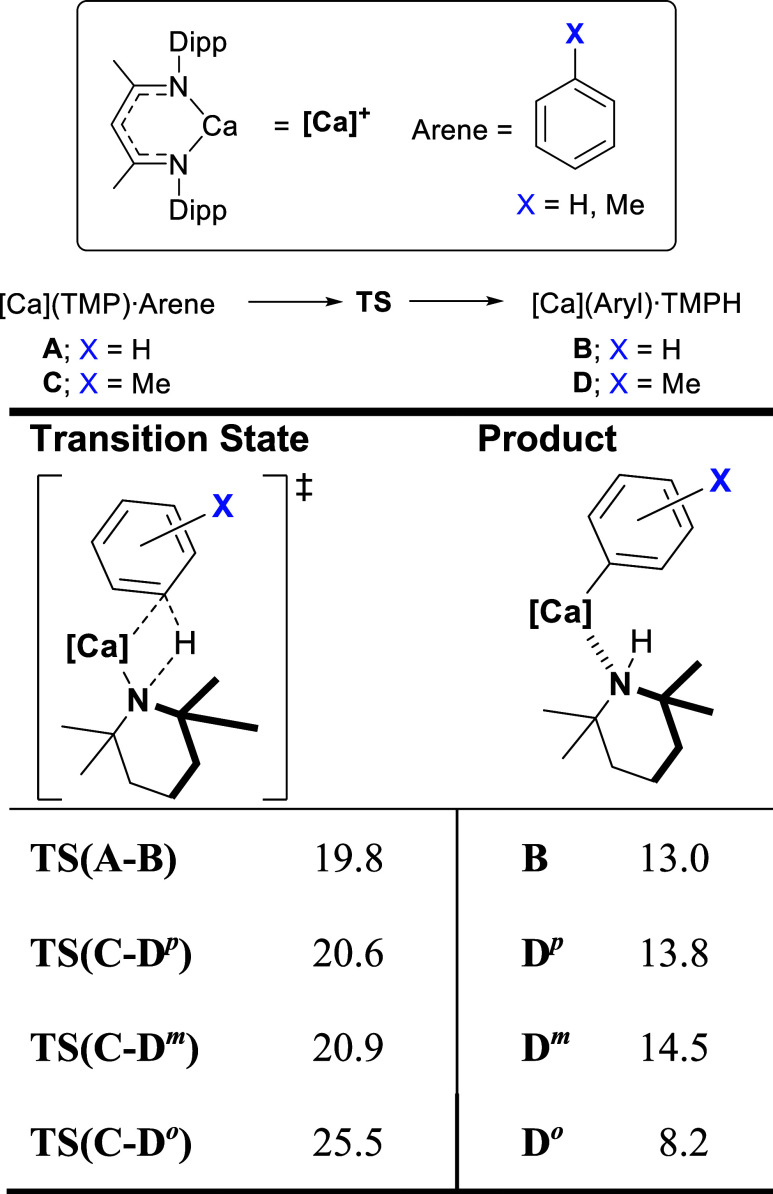
DFT-Calculated Free Energies (BP86-D3^BJ^(SMD = Toluene)/def2-TZVPP//BP86/def2-SVP level, in kcal mol^–1^) for the Formation of **B** and **D** through the C­(sp^2^)-H Deprotonation of Benzene and Toluene by **A** and **C**, respectively

## Conclusion

Reactions of [(BDI)­CaH]_2_ with dialkylzinc reagents occur in a 1:2 ratio via a defined sequence of dialkyl­(hydrido)­zincate formation, calcium alkyl extrusion and further transmetalation of both the organyl and spectator BDI ligands to zinc.^124,125^ The current work demonstrates that similar equilibria are established from treatment of [(BDI)­CaH]_2_ with zinc amides. Reaction with [Zn­{N­(SiMe_3_)_2_}_2_] provides a zincate species, [(BDI)­Ca­{N­(SiMe_3_)_2_}­(μ-H)­Zn]_2_, arising from the 1:1 reaction of the calcium and zinc reagents. While this heterobimetallic compound can be isolated, and [(BDI)­Ca­{N­(SiMe_3_)_2_}] is also identified in the initially formed solution, the system is ultimately perturbed toward exchange of both the hydride and BDI ligands from calcium to zinc to afford [(BDI)­ZnH]. A further calcium amidozincate, [(BDI)­Ca­(μ-N­{C­(CH_3_)_2_CH_2_}_2_­CH)­(μ-H)­Zn­(μ-H)]_2_, and the calcium amide, [(BDI)­Ca­(TMP)], result from the reaction of [(BDI)­CaH]_2_ and [Zn­(TMP)_2_]. More significantly, when performed in nonactivated arene solvents, this system results in a cascade of C­(*sp* ^2^)-H deprotonation and ligand transmetalation to afford the corresponding β-diketiminato zinc aryls. Although the viability of this process will be necessarily influenced by the lattice enthalpy of byproducts such as saline CaH_2_ (Δ*H*_latt_ = −2172 kJ mol^–1^),^143^ even when formed *in situ*, the resultant zinc aryl may be utilized in biaryl formation via a telescoped Negishi coupling. Irrespective of the relevance of [(BDI)­Ca­(μ-N­{C­(CH_3_)_2_CH_2_}_2_CH)­(μ-H)­Zn­(μ-H)]_2_ and [(BDI)­Ca­(TMP)] to initial solution speciation, the complexity of this system dictates that the actual mode of arene metalation remains to be identified. While [(BDI)­Ca­(TMP)] readily deprotonates toluene, calculations identify the resultant benzyl product of C­(*sp* ^3^)-H activation as both the kinetic and thermodynamic reaction product. While it is, thus, appealing to ascribe greater significance to [(BDI)­Ca­(μ-N­{C­(CH_3_)_2_CH_2_}_2_CH)­(μ-H)­Zn­(μ-H)]_2_, the lability of this species mitigates against confident assessment of its reactivity. Consequently, aryl anion formation remains dependent either on further, as yet unidentified, bimetallic species or the synergic cooperation of equilibrating mono- or bimetallic components. Although it remains something of a “black box”,^144,145^ therefore, this system provides some commonality with Mulvey and Hevia’s articulation of “trans metal trapping”, whereby a high basicity arises from the introduction of the heavier alkaline earth reagent but with the zinc center performing as the instrument of aryl anion trapping.^146^ We are coninuing to study this reactivity and to elaborate further bimetallic systems derived from alternative heteroanions.

## Supplementary Material



## Notes

Experimental details, NMR spectra, X-ray crystallography, computational details, and atomic coordinates for the optimized geometries of the compounds (PDF)
