# Supplementary material for: Arene Activation by Calcium Hydride/Zinc Amide Equilibration
Source: J Am Chem Soc. 2025 Aug 2;147(32):29554–67. doi: 10.1021/jacs.5c10735 (PMC12356595; doi:10.1021/jacs.5c10735)
Supplement: Supplementary file 1 [file ja5c10735_si_001.pdf]

Supplementary Information for

Arene Activation by Calcium Hydride/Zinc Amide Equilibration

Kyle G. Pearce,* Agustín Morales, Michael S. Hill,* Mary F. Mahon and Claire L. McMullin*

Department of Chemistry, University of Bath, Claverton Down, Bath, BA2 7AY

Synthetic Details S2

General Considerations S2

Reaction Between [(BDI)CaH]$_2$ and [ZnHMDS$_2$] S2

Synthesis of [(BDI)Ca(N(SiMe$_3$)$_2$)(μ-H)Zn]$_2$ (1) S2

Reaction Between [(BDI)CaH]$_2$ and [Zn(TMP)$_2$] S3

Synthesis of [(BDI)Ca(μ-N{C(CH$_3$)$_2$CH$_2$}$_2$CH)(μ-H)Zn(μ-H)]$_2$ (3) S3

Synthesis of [(BDI)Ca(TMP)] (7) S3

Reaction Between [(BDI)Ca(TMP)] (7) and C$_6$D$_6$ S4

Synthesis of [(BDI)Ca(CH$_2$C$_6$H$_5$)] (8) S4

General Procedure to Prepare [(BDI)ZnAr] S4

[(BDI)ZnPh] (2) S5

[(BDI)Zn-3-MePh] (4^m) and [(BDI)Zn-4-MePh] (4^p) S5

[(BDI)Zn-CH$_2$-Ph-3,5-Me] (5) S5

[(BDI)Zn-(3,5-^{t}Bu)$_2$Ph] (6) S5

NMR Spectra S6

Crystallographic Data S25

Computational Details S31

References S36

Synthetic Details

General Considerations

All manipulations were carried out using standard Schlenk line and glovebox techniques under an inert atmosphere of argon. NMR experiments were conducted in J-Young's NMR tubes and prepared in a glovebox. NMR spectra were recorded on a Bruker BioSpin GmbH spectrometer operating at 400.13 MHz (^{1}H) and 100.62 MHz (^{13}C). Elemental analyses were performed at Elemental Microanalysis Ltd., Okehampton, Devon, UK or by the Elemental Analysis Services Team at London Metropolitan University. Solvents were dried by passage through a commercially available solvent purification system and stored under argon in ampoules over 4 Å molecular sieves. C_6D_6, C_7D_8, mesitylene and 1,3-*tert*-butylbenzene were purchased from Merck, dried over potassium, distilled and stored over molecular sieves. $ZnHMDS_2$ (HMDS = $N(SiMe_3)_2$) was purchased from Merck, transferred to a J-Young's ampoule and used without further purification. $Pd(PCy_3)_2$ (cy = $(CH_2)_5C$), was purchased from Fluorochem and used without purification.TMP-H (TMP = $N\{C(CH_3)_2CH_2\}_2CH$) and bromobenzene were dried over CaH_2 and distilled prior to use. $[(BDI)CaH]_2$ (BDI = $HC\{(Me)CN\text{-}2,6\text{-}i\text{-}Pr_2C_6H_3\}_2$),[1] and $[Zn(TMP)_2]$ were synthesised according to literature procedures.[2]

Reaction Between $[(BDI)CaH]_2$ and $[ZnHMDS_2]$

$ZnHMDS_2$ (13.16 µl, 0.033 mmol) was added to a J. Young's NMR tube containing a C_6D_6 (*ca.* 0.6 cm^3) solution of $[(BDI)CaH]_2$ (15 mg, 0.016 mmol). The solution was monitored over the next 8 hours, observing the formation and consumption of **$[(BDI)Ca(N(SiMe_3)_2)(\mu\text{-}H)Zn(\mu\text{-}H)]_2$** (**1**) and [(BDI)CaHMDS]. After 16 hours only [(BDI)ZnH] was observed spectroscopically (**Figure S4**), alongside a small amount of precipitate within the NMR tube, assumed to be $[Ca(HMDS)H]_\infty$. The reaction mixture was decanted away from the precipitate and concentrated under reduced pressure to afford [(BDI)ZnH] as a colourless solid. Yield: 13.4 mg, 84%.

This reaction has been performed in both benzene and toluene and observed to provide the same reaction outcome. The spectroscopic signatures of [(BDI)CaHMDS] and [(BDI)ZnH] are consistent with the literature.[3,4]

Synthesis of $[(BDI)Ca(N(SiMe_3)_2)(\mu\text{-}H)Zn]_2$ (1)

$[(BDI)CaH]_2$ (30 mg, 0.033 mmol) and $[ZnHMDS_2]$ (26.32 µl, 0.065 mmol) were introduced into a vial and dissolved in toluene (*ca.* 1 cm^3). Once all solids were dissolved, the reaction vial was placed in the freezer (–35 °C), affording colourless crystals after 16 hours. The supernatant was decanted, and the crystals were crushed and dried *in vacuo*. Yield: 36 mg, 40%. Compound **1** continues to react, even at –20 °C, therefore, only the ^{1}H NMR spectrum is tentatively assigned.

^1H NMR (C_7D_8): δ = 4.82 (s, H̲CCN, 2H), 3.17 (br m, H̲C(CH_3)$_2$, 8H) 1.71 (s, NCCH̲$_3$, 6H), 1.65 (s, , 6H) 1.33-1.16 (overlapping, HC(CH̲$_3$)$_2$, 48H), 0.21 (s, NSiM̲e̲$_3$, 9H), 0.12 (s, NSiM̲e̲$_3$, 9H), 0.07 (s, NSiM̲e̲$_3$, 18H).

Anal. Calc. for $C_{70}H_{120}N_6Si_4Zn_2Ca_2$: C, 61.41; H, 8.84; N, 6.14. Found: C, 63.51; H, 9.08; N, 5.94.

Reaction Between [(BDI)CaH]$_2$ and [Zn(TMP)$_2$]

[Zn(TMP)$_2$] (16.2 mg, 0.047 mmol) and [(BDI)CaH]$_2$ (22.1 mg, 0.024 mmol) were introduced into a J. Young's NMR tube and dissolved in C_6D_6 (*ca.* 0.6 cm^3). After 16 hours only [(BDI)ZnPh] and TMP-D were observed spectroscopically (**Figure S7**), alongside a small amount of precipitate within the NMR tube, assumed to be [Ca(TMP)H]$_\infty$.

This reaction was also performed with C_6H_6. After 16 hours the reaction mixture was decanted away from the small amount of precipitate present and concentrated under reduced pressure. The crude solid was washed with cold hexane to afford [(BDI)ZnPh] (**2**) as a colourless solid. Yield: 17.6 mg, 67% (**Figure S9**).

The spectroscopic signatures of **2** and TMP-D are consistent with the literature.[5,6]

Synthesis of [(BDI)Ca(μ-N{C(CH$_3$)$_2$CH$_2$}$_2$CH)(μ-H)Zn(μ-H)]$_2$ (3)

[(BDI)CaH]$_2$ (24 mg, 0.026 mmol) and [Zn(TMP)$_2$] (18.1 mg, 0.052 mmol) were combined in a vial, dissolved in benzene (*ca.* 1 cm^3) and placed on top of the freezer. A small number of colourless crystals started to form, away from which the supernatant was decanted and washed with cold (–35 °C) hexane and dried under reduced pressure. Yield: 5 mg, 14%.

Unlike most of the other calcium zincate complexes, **3** does not crystallise from toluene at –35 °C. Compound **3** continues to react in solution even at –40 °C, therefore only a ^1H NMR is reported and the characteristic resonances tentatively assigned.

^1H NMR (C_7D_8): δ = 5.01 (s, Ca-μ-H̲-Zn, 2H), 4.68 (s, H̲CCN, 2H), 3.43 & 3.37 (overlapping multiplets, H̲C(CH_3)$_2$, 8H).

Synthesis of [(BDI)Ca(TMP)] (7)

Hexane (1 cm^3) was added to a vial containing [(BDI)CaH]$_2$ (43.87 mg, 0.048 mmol) and [Zn(TMP)$_2$] (33 mg, 0.095 mmol). The reaction mixture was agitated until all solids dissolved, observing a pale-yellow colouration and the vial was placed in the freezer (–35 °C), affording pale-yellow crystals after 16 hours. The supernatant was decanted away from the crystals and **4** was washed with cold hexane (–35 °C, *ca.* 3 x 0.25 cm^3). Yield: 16.8 mg, 29%.

^1H NMR (C_7D_8): δ = 7.34-7.17 (m, Ar-H, 6H), 4.87 (s, H̲CCN, 1H), 3.22 (hept, H̲C(CH_3)$_2$, $^3J_{HH}$ = 6.87 Hz, 4H), 1.83 (br t, N{(CCH$_3$)$_2$CH$_2$}$_2$CH̲$_2$, 2H), 1.71 (s, NCCH̲$_3$, 6H), 1.34 (d, HC(CH̲$_3$)$_2$, $^3J_{HH}$ = 6.87

Hz, 12 H), 1.27 (br s, $N\{(CCH_3)_2C\underline{H}_2\}_2CH_2$, 2H), 1.21 (d, $HC(C\underline{H}_3)_2$, $^3J_{HH}$ = 6.87 Hz, 12 H), 0.96 (br s, $N\{(CC\underline{H}_3)_2CH_2\}_2CH_2$,12H).

$^{13}C\{^1H\}$ NMR (C_7D_8): δ = 165.6 ($N\underline{C}CH_3$), 145.7 (*i*-Ar-C), 140.9 (Ar-C), 124.3 (Ar-C), 94.2 ($H\underline{C}CN$), 51.1 ($N\{(\underline{C}CH_3)_2CH_2\}_2CH_2$), 39.9 ($N\{(CCH_3)_2\underline{C}H_2\}_2CH_2$), 34.4 ($N\{(C\underline{C}H_3)_2CH_2\}_2CH_2$), 28.5 ($H\underline{C}(CH_3)$), 25.0 ($HC(\underline{C}H_3)$), 24.6 ($HC(\underline{C}H_3)$), 23.3 ($NC\underline{C}H_3$), 20.0 ($N\{(CCH_3)_2CH_2\}_2\underline{C}H_2$, under d_8-tol peak, identified by HSQC.

Note: NMR analyses in aromatic solvents must be performed at or below –40 °C to limit reaction with the solvent.

Anal. Calc. for $C_{38}H_{59}N_3Ca_1$: C, 76.33; H, 9.95; N, 7.03. Found: C, 75.33; H, 9.92; N, 6.63.

Reaction Between [(BDI)Ca(TMP)] (7) and C_6D_6

[(BDI)CaTMP] (**7**) (23 mg, 0.04 mmol) was dissolved in C_6D_6 (*ca.* 1 cm^3) and monitored by ^1H NMR spectroscopy. Compound **7** readily reacts with C_6D_6, albeit an intractable mixture of unidentifiable products were formed (**Figure S15**).

Synthesis of [(BDI)Ca($CH_2C_6H_5$)] (8)

[(BDI)Ca(TMP)] (**7**) (11 mg, 0.018 mmol) was dissolved in protio-toluene (*ca.* 1 cm^3) and placed on top of the freezer, affording yellow needle-like crystals of **8** over 8 hours. The contents of the vial were decanted into a filter pipette, the supernatant was filtered, and the yellow needle-like crystals of **8** were washed with hexane (3 x 1 cm^3). Yield: 19.5 mg, 89%.

This reaction still occurs at –35 °C, although no better crystals could be grown at this temperature. Dissolution of **8** in d_8-THF and immediate spectroscopic analysis, allows for the observation of the characteristic benzyl resonances by ^1H NMR spectroscopy, although decomposition is also immediately observed.

^1H NMR (C_4D_8O): δ = 6.41 (t, *o*-Ar-H, $^3J_{HH}$ = 8.01 Hz, 2H), 5.95 (d,*m*-Ar-H, $^3J_{HH}$ = 8.01 Hz, 2H), 5.74 (t, p-Ar-H, $^3J_{HH}$ = 8.01 Hz, 1H), 4.73 (s, $\underline{H}CCN$, 1H), 3.24 (hept, $\underline{H}C(CH_3)_2$, $^3J_{HH}$ = 6.84 Hz, 4H) 2.31 (s, Ca-CH_2), 1.64 (s, $NCC\underline{H}_3$, 6H), 1.26 (d, $HC(C\underline{H}_3)_2$, $^3J_{HH}$ = 6.89 Hz, 12 H), 1.18 (d, $HC(C\underline{H}_3)_2$, $^3J_{HH}$ = 6.89 Hz, 12 H).

Anal. Calc. for $C_{36}H_{48}N_2Ca_1$: C, 78.78; H, 8.82; N, 5.10. Found: C, 77.02; H, 6.37; N, 4.98.

General Procedure to Prepare [(BDI)ZnAr]

$[(BDI)CaH]_2$ (1 equiv.) and $[Zn(TMP)_2]$ (2 equiv.) were introduced into a J-Young's NMR tube and the aromatic solvent of choice (toluene, benzene, mesitylene or 1,3-tert-butylbenzene) was added (0.6 cm^3) and the reaction mixture was heated at 60 °C for 16 hours. The volatiles were removed under reduced pressure and the crude oil was washed with cold hexane (–35 °C), affording the corresponding [(BDI)ZnAr] complex as a colourless solid in each case.

[(BDI)ZnPh] (2)

Yield: 28 mg, 86%. ^{1}H NMR (C_6D_6): δ = 7.25-7.15 (m, ^{Dipp}Ar-H, 6H), 6.96 (m, ^{Ph}Ar-H, 3H), 6.59-6.56 (m, ^{Ph}Ar-H, 2H), 5.06 (s, $\underline{H}$CCN,1H), 3.24 (hept., $\underline{H}C(CH_3)_2$, $^{3}J_{HH}$ = 6.9 Hz, 4H), 1.75 (s, NCC$\underline{H}_3$, 6H), 1.20 (d, HC(C$\underline{H}_3)_2$, $^{3}J_{HH}$ = 6.9 Hz, 12H), 1.16 (d, HC(C$\underline{H}_3)_2$, $^{3}J_{HH}$ = 6.9 Hz, 12H). In agreement with literature values.[5]

[(BDI)Zn-3-MePh] (4*^{m}*) and [(BDI)Zn-4-MePh] (4*^{p}*)

Yield: 29.2 mg, 85%. **Note**: These compounds could not be separated from one another but were independently identified by mechanical selection of individual single crystals. **4*^{m}*** was identified as the major component through screening experiments, whilst **4*^{p}*** is the minor component, determined to be present in a respective 82:18 ratio by ^{1}H NMR spectroscopy.

[(BDI)Zn-3-MePh] (**4*^{m}***) (^{1}H NMR (C_6D_6): δ = 5.06 (s, $\underline{H}$CCN, 1H), 3.25 (hept, $\underline{H}C(CH_3)_2$, $^{3}J_{HH}$ = 6.88 Hz, 4H), 2.03 (s, 3-$\underline{Me}$Ph, 3H) 1.77 (s, NCC$\underline{H}_3$, 6H). ^{13}C{^{1}H} NMR (C_7D_8): δ = 167.6 (N$\underline{C}CH_3$), 95.1 (H$\underline{C}$CN).

[(BDI)Zn-4-MePh] (**4*^{p}***) ^{1}H NMR (C_6D_6): δ = 4.97 (s, $\underline{H}$CCN, 1H), 3.09 (hept, $\underline{H}C(CH_3)_2$, $^{3}J_{HH}$ = 6.85 Hz, 4H), 1.96 (s, 3-$\underline{Me}$Ph, 3H) 1.68 (s, NCC$\underline{H}_3$, 6H). ^{13}C{^{1}H} NMR (C_7D_8): δ = 167.5 (N$\underline{C}CH_3$), 95.07 (H$\underline{C}$CN).

[(BDI)Zn-CH_2-Ph-3,5-Me] (5)

Yield: 32.9 mg, 90%. ^{1}H NMR (C_6D_6): δ = 7.20-7.14 (m, ^{Dipp}Ar-H, 6H), 6.45 (s, ^{Mes}*p*-ArH, 1H), 6.11 (s, ^{Mes}*m*-ArH, 2H), 4.99 (s, $\underline{H}$CCN, 1H), 3.12 (hept, $\underline{H}C(CH_3)_2$, $^{3}J_{HH}$ = 6.84 Hz, 4H), 2.10 (s, Ar-*m*-$(CH_3)_2$, 6H), 1.81 (s, Zn-C$\underline{H}_2$-Ar, 2H), 1.70 (s, NCC$\underline{H}_3$, 6H), 1.14 (overlapping d, HC(C$\underline{H}_3)_2$, 24 H).
^{13}C{^{1}H} NMR (C_6D_6): δ = 167.9 (N$\underline{C}CH_3$), 147.4 (^{Mes}*i*-Ar-C), 144.7 (^{Dipp}*i*-Ar-C), 141.6 (Ar-C), 136.8 (Ar-C), 126.2 (Ar-c), 124.9 (Ar-C), 123.9 (^{Mes}*p*-ArC), 123.1 (^{Mes}*m*-ArH), 95.5 (H$\underline{C}$CN), 28.6 (H$\underline{C}(CH_3)_2$), 23.9 (H$\underline{C}(CH_3)_2$), 23.6 (H$\underline{C}(CH_3)_2$), 23.3 (NC$\underline{C}H_3$), 21.7 (Mes-$\underline{C}H_3$), 17.0 (Zn-$\underline{C}H_2$-Ar).
Anal. Calc. for $C_{70}H_{112}N_4Zn_2Ca_2$: C, 75.79; H, 8.70; N, 4.65. Found: C, 74.54; H, 8.69; N, 4.56.

[(BDI)Zn-(3,5-^{t}Bu)$_2$Ph] (6)

Yield: 41.9 mg, 89%. ^{1}H NMR (C_6D_6): δ = 7.54 (s, ^{Dipp}Ar-H, 2H), 7.29 (br t, *p*-(^{t}Bu)$_2$Ar-H, $^{3}J_{HH}$ =1.88 Hz, 1H), 7.24 – 7.19 (m, ^{Dipp}Ar-H, 4H), 6.38 (d, *m*-(^{t}Bu)$_2$Ar-H, $^{3}J_{HH}$ =1.88 Hz, 2H), 5.05 (s, $\underline{H}$CCN, 1H), 3.25 (hept, $\underline{H}C(CH_3)_2$, $^{3}J_{HH}$ = 6.93 Hz, 4H), 1.76 (s, NCC$\underline{H}_3$, 6H), 1.28 (s, $C(CH_3)_3$, 18H), 1.19 (overlapping dd, HC(C$\underline{H}_3)_2$, $^{3}J_{HH}$ = 6.93 Hz, 24 H).
^{13}C{^{1}H} NMR (C_6D_6): δ = 167.9 (N$\underline{C}CH_3$), 150.8 (Ar-C), 147.7 (*i*-Ar-C), 147.4 (*i*-Ar-C), 145.2 (Ar-C), 141.9 (Ar-C), 134.6 (*m*-(^{t}Bu)$_2$Ar-C), 126.4 (Ar-C), 124.3 (Ar-C), 122.9 (Ar-C), 122.2 (Ar-C), 120.1 (*p*-(^{t}Bu)$_2$Ar-C), 95.4 (H$\underline{C}$CN), 34.9 ($\underline{C}(CH_3)_3$), 31.6 (C($\underline{C}H_3)_3$), 28.6 (H$\underline{C}(CH_3)_2$), 24.8 (HC($\underline{C}H_3)_2$), 23.5 (HC($\underline{C}H_3)_2$), 23.2 (NC$\underline{C}H_3$).

Telescoped Negishi Cross-Coupling

$[(BDI)CaH]_2$ (6.65 mg, 0.007 mmol) and $[Zn(TMP)_2]$ (5 mg, 0.0145 mmol) were introduced into a J-Young's NMR tube, protio-benzene was added (0.6 cm^3) and the reaction mixture was heated was heated at 60 °C for 16 hours, observing the formation of [(BDI)ZnPh] (**Figure S34**). The volatiles were removed under reduced pressure, PhBr (1.5 µl, 0.0145 mmol), $Pd(PCy_3)_2$ (0.05 equiv., 0.5 mg, 0.0007 mmol) and C_6D_6 (0.6 cm^3) were added and the reaction mixture was heated at 80 °C for 2 days. Hexamethylbenzene (4.5 mg) was added, and the amount of biphenyl formation was quantified. Yield: 99%, determined by ^{1}H NMR spectroscopy, compared against hexamethylbenzene.

NMR Spectra

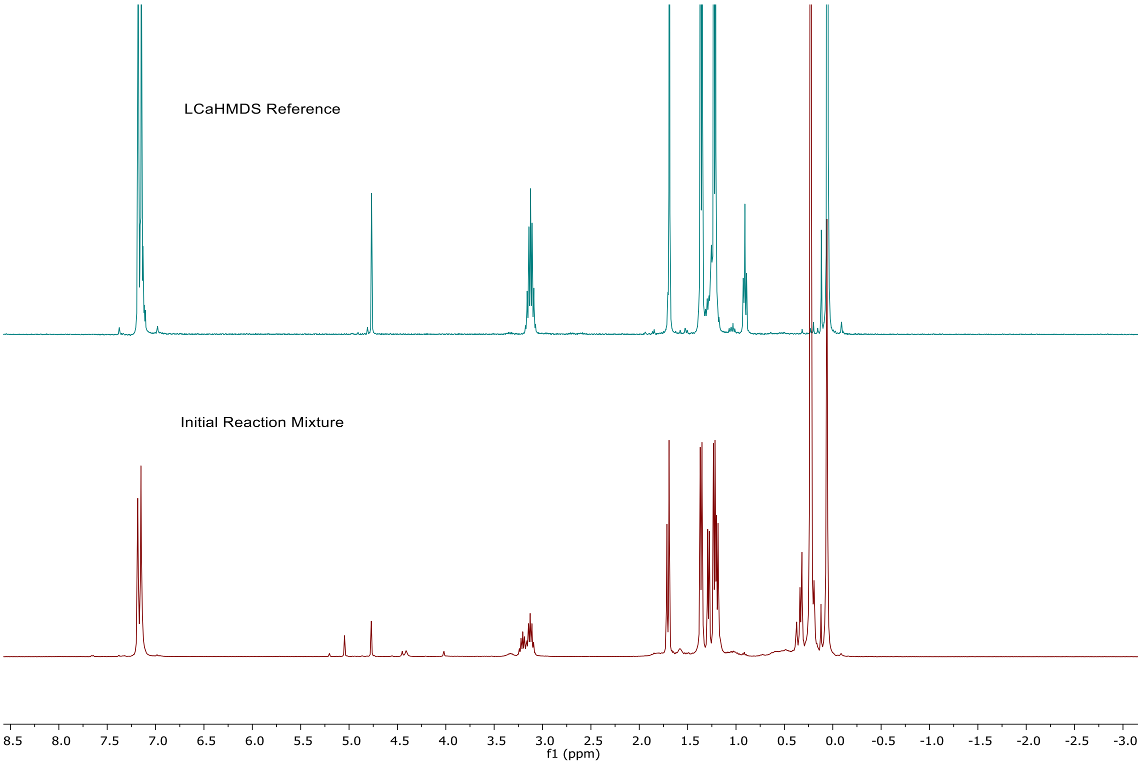

Figure S1. *Overlayed ^{1}H NMR Spectra (C_7D_8, 298 K, 400.13 MHz) from adding [$ZnHMDS_2$] to $[(BDI)CaH]_2$, demonstrating the formation of [(BDI)CaHMDS] and [(BDI)ZnH].*

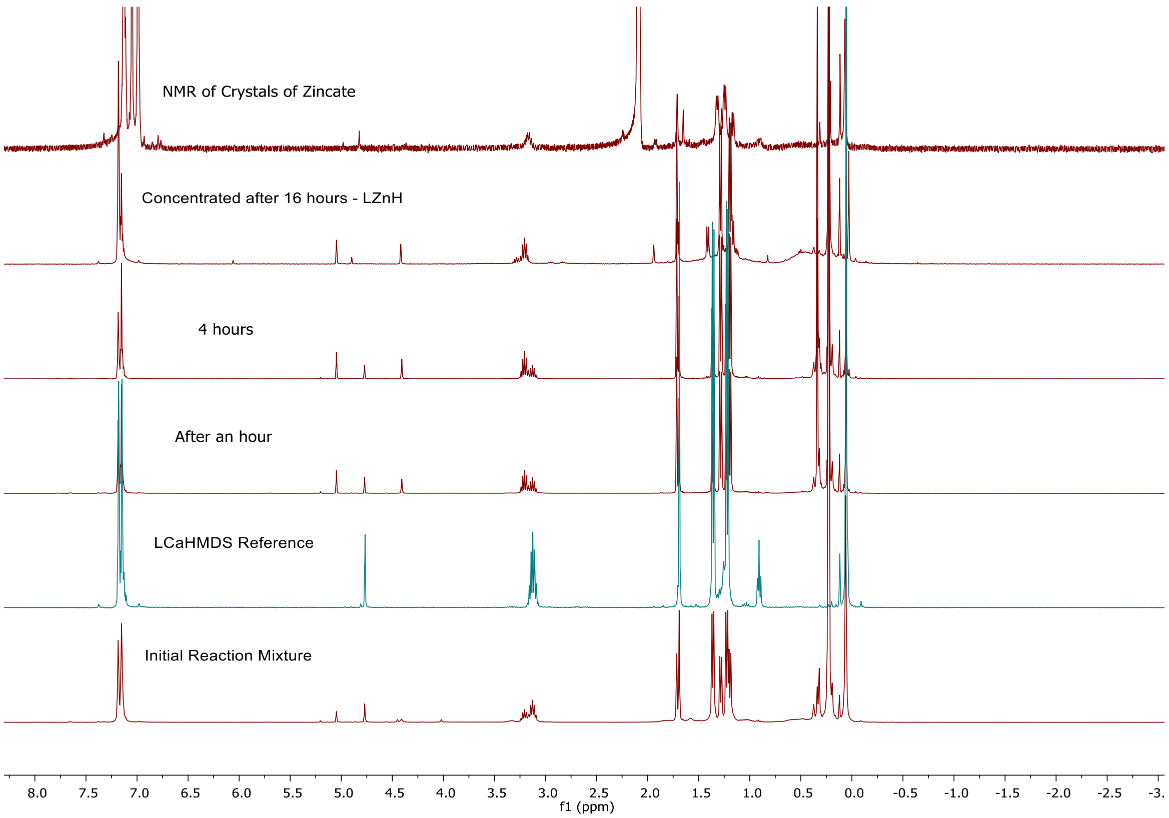

Figure S2. *Overlayed* ^{1}H *NMR Spectra (*C_7D_8*, 298 K, 400.13 MHz) from adding [*$ZnHMDS_2$*] to [(BDI)CaH]*$_2$*, demonstrating the formation of [(BDI)CaHMDS] and [(BDI)ZnH].*

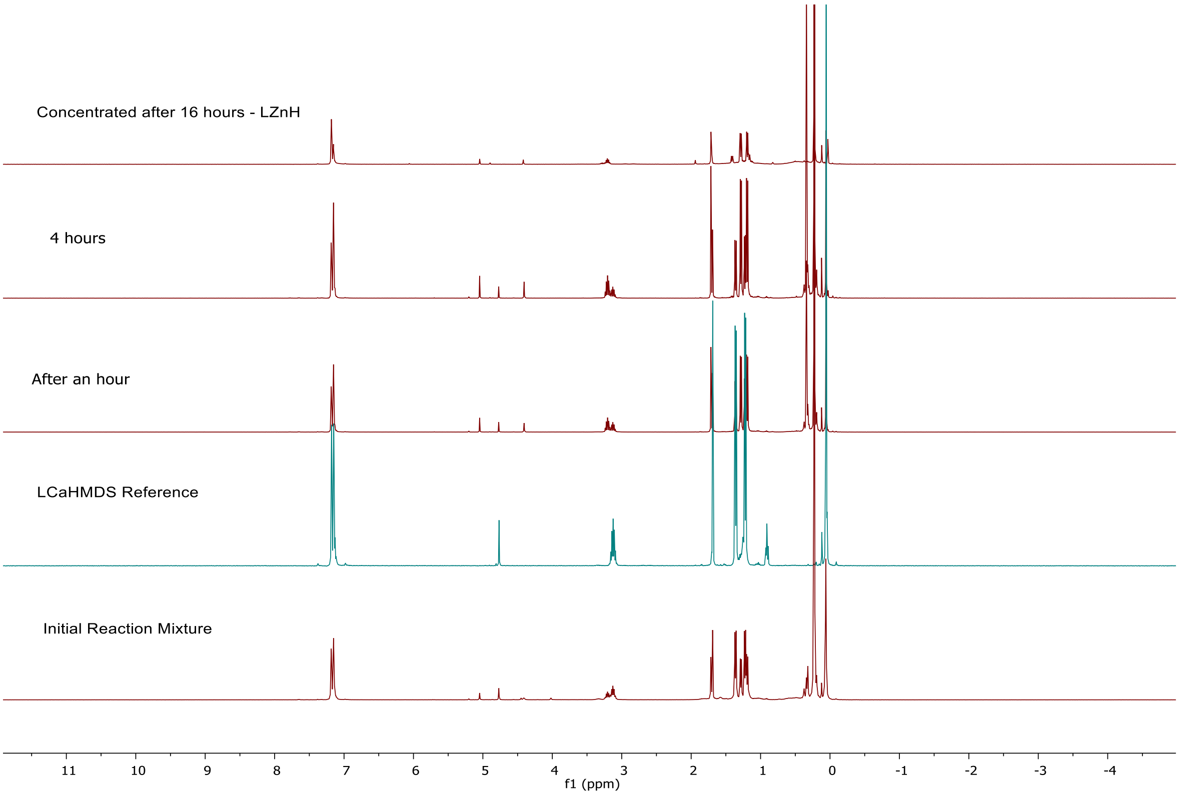

Figure S3. *Overlayed* ^{1}H *NMR Spectra (*C_7D_8*, 298 K, 400.13 MHz) from adding [*$ZnHMDS_2$*] to [(BDI)CaH]*$_2$*, demonstrating the formation of [(BDI)CaHMDS] and [(BDI)ZnH].*

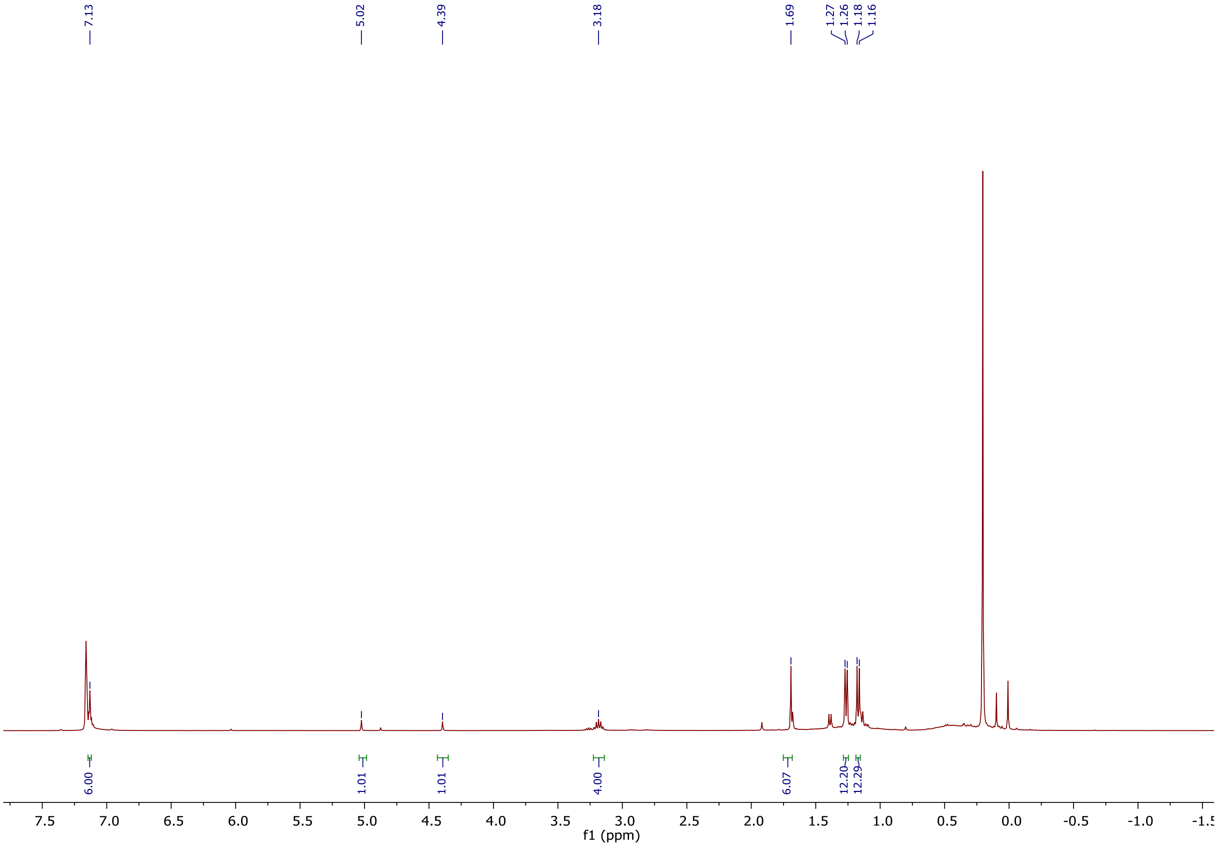

Figure S4. *^{1}H NMR Spectrum (C_7D_8, 298 K, 400.13 MHz) for* ***[(BDI)ZnH]*** *in reaction mixture, formed after 16 hours.*

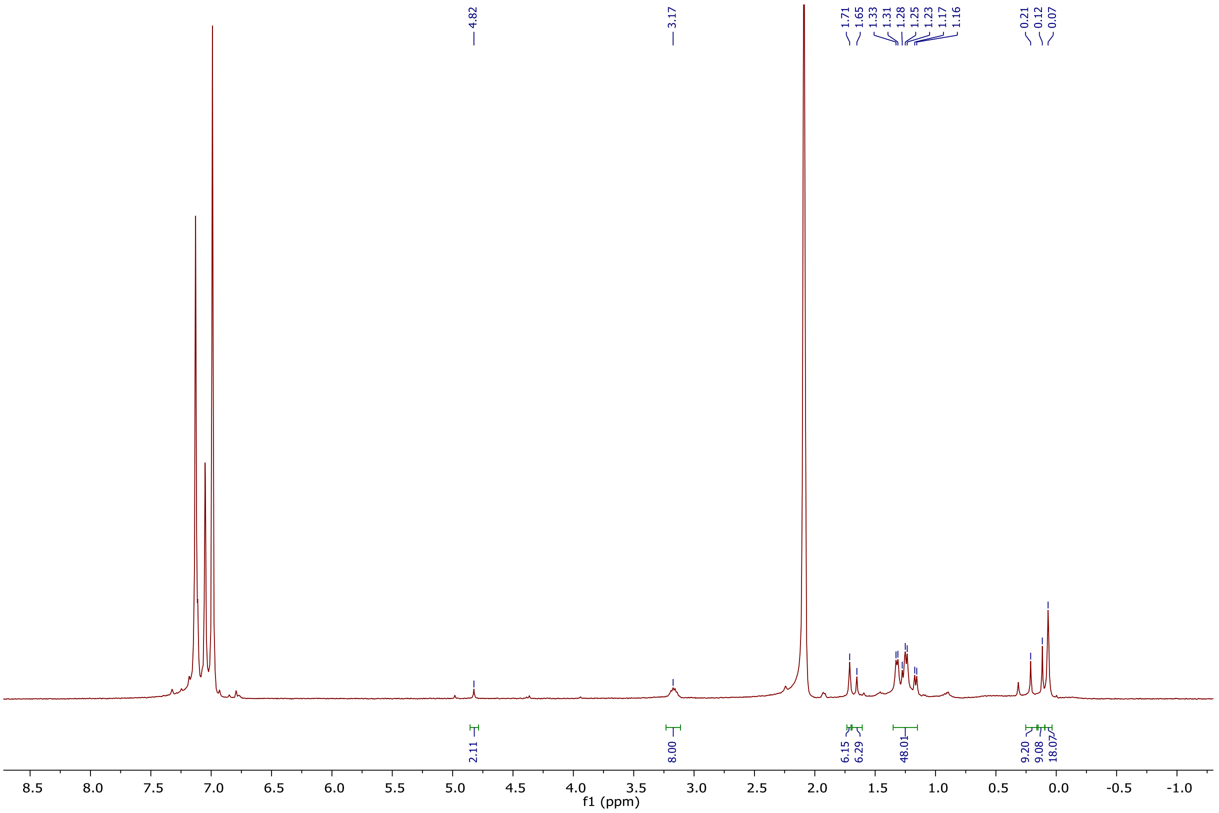

Figure S5. *^{1}H NMR Spectrum (C_7D_8, 253 K, 400.13 MHz) for* ***[(BDI)Ca(N(SiMe$_3$)$_2$)(μ-H)Zn]$_2$ (1).***

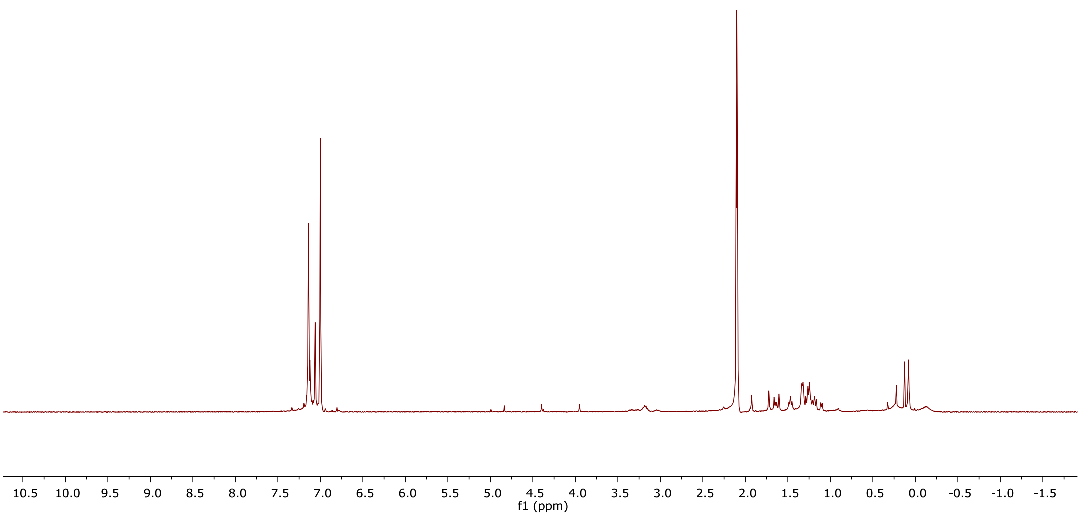

Figure S6. *^{1}H NMR Spectrum (C_7D_8, 253 K, 400.13 MHz) after leaving* ***[(BDI)Ca(N(SiMe$_3$)$_2$)(μ-H)Zn]$_2$ (1)*** *at 253k for 16 hours.*

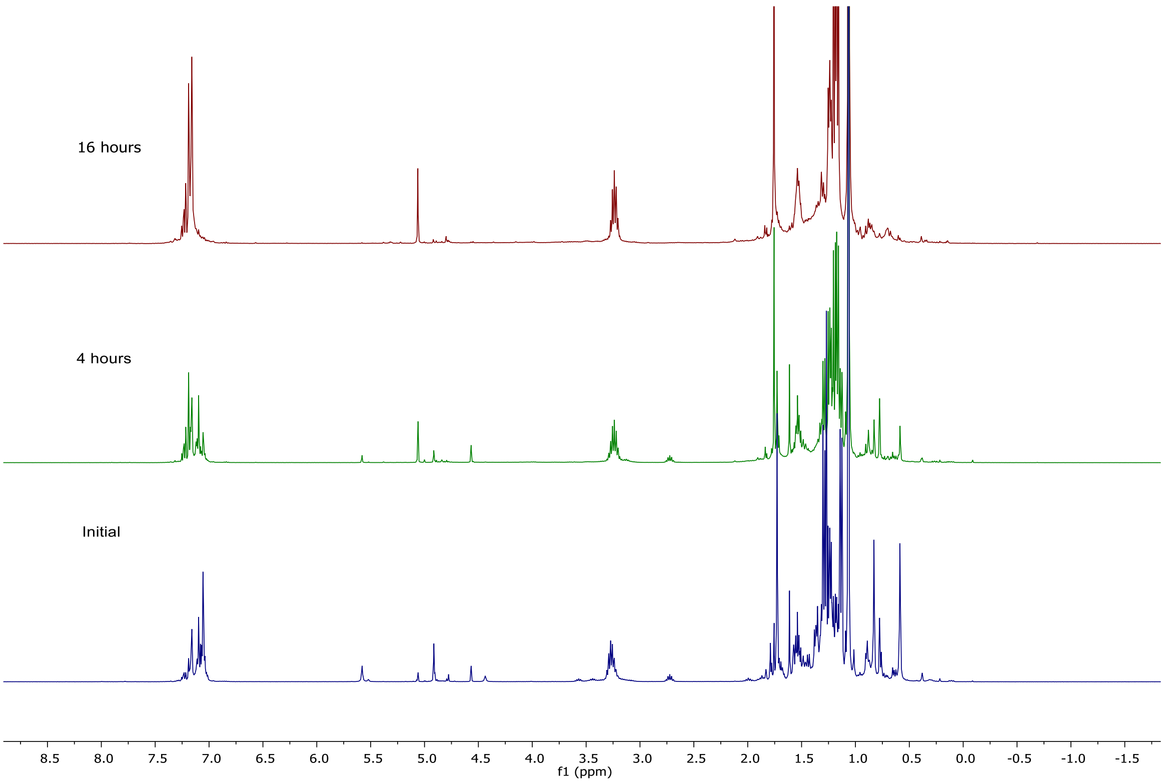

Figure S7. *Overlayed ^{1}H NMR Spectra (C_6D_6, 298 K, 400.13 MHz) from adding [Zn(TMP)$_2$] to [(BDI)CaH]$_2$, with [(BDI)ZnPh]* ***(2)*** *effectively produced immediately.*

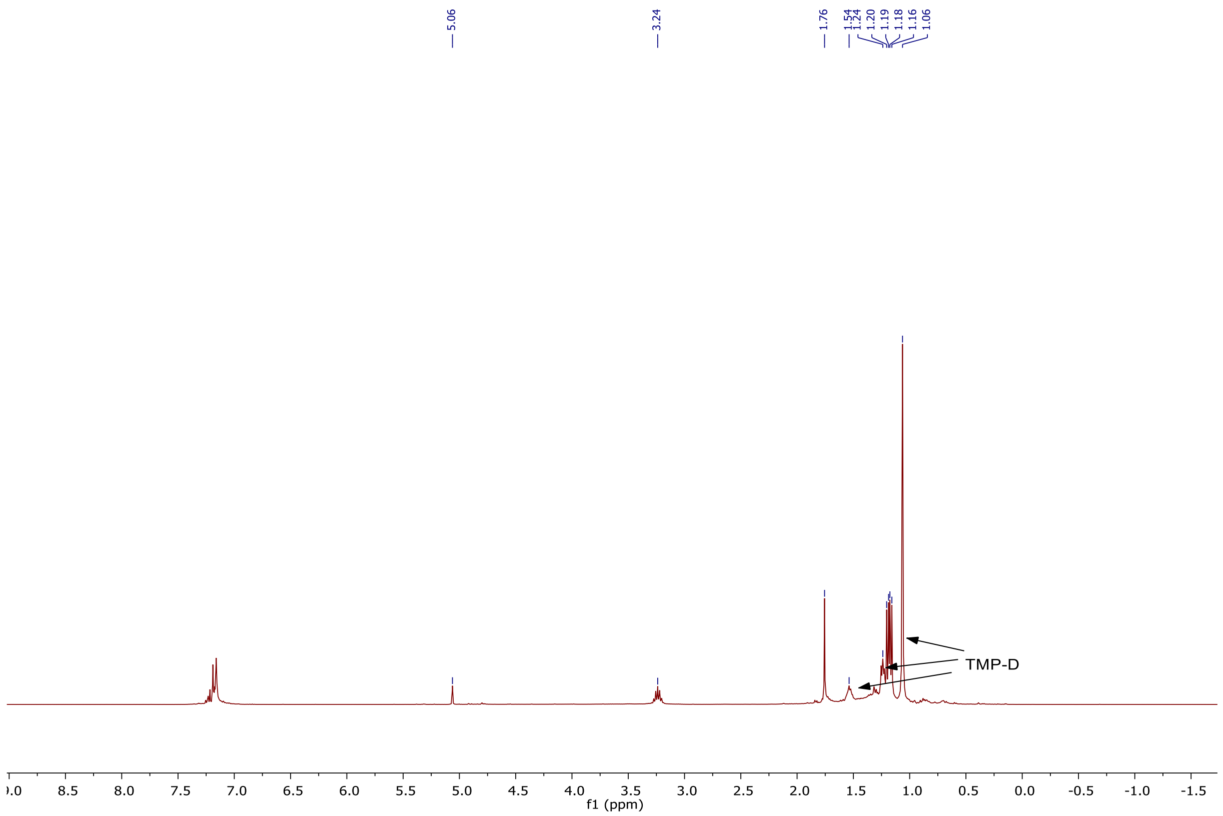

Figure S8. *^{1}H NMR Spectrum (C_6D_6, 298 K, 400.13 MHz) for* ***[(BDI)ZnC₆D₅] (2-d)*** *and TMP-D from reaction mixture.*

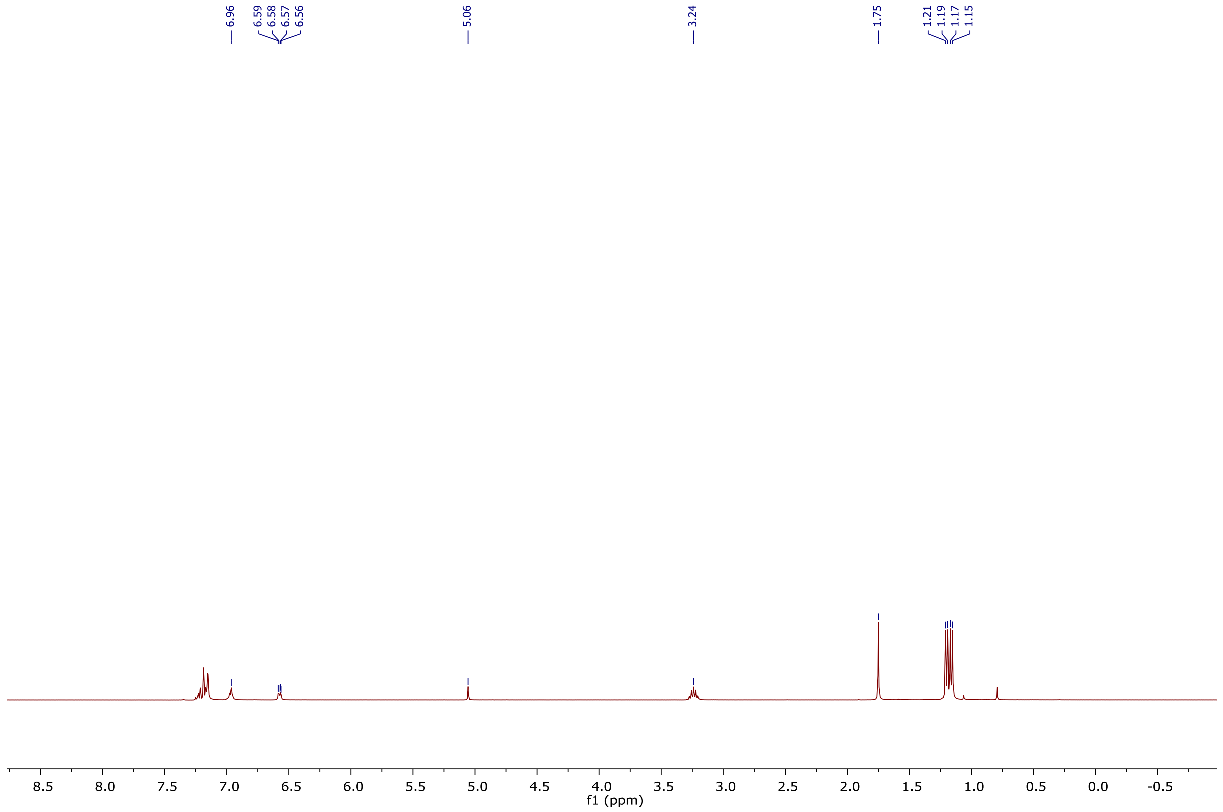

Figure S9. *^{1}H NMR Spectrum (C_7D_8, 298 K, 400.13 MHz) for* ***[(BDI)ZnPh] (2)****, after hexane wash.*

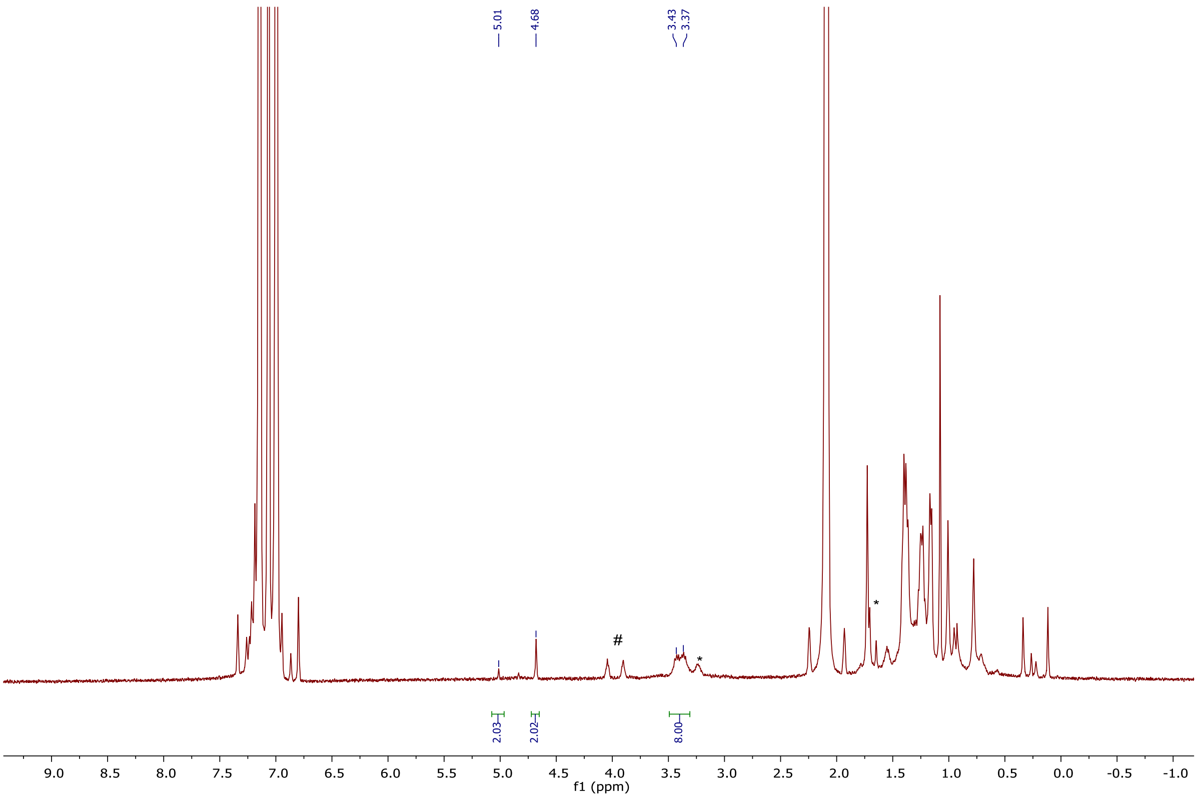

Figure S10. *^{1}H NMR Spectrum (C_7D_8, 253 K, 400.13 MHz) for* ***[(BDI)Ca(μ-N{C(CH$_3$)$_2$CH$_2$}$_2$CH)(μ-H)Zn(μ-H)]$_2$ (3)****. # = impurity in d_8-tol. * = [(BDI)CaTMP].*

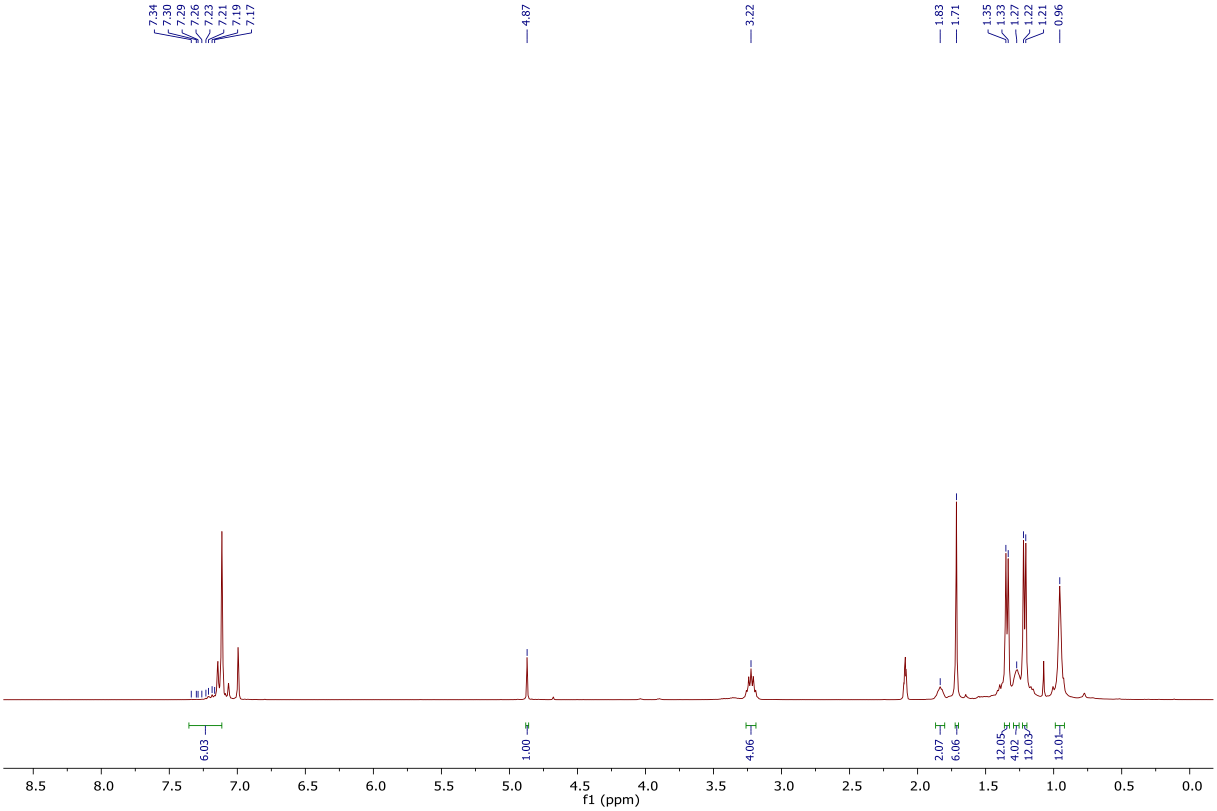

Figure S11. *^{1}H NMR Spectrum (C_7D_8, 233 K, 400.13 MHz) for* ***[(BDI)Ca(TMP)] (7)****.*

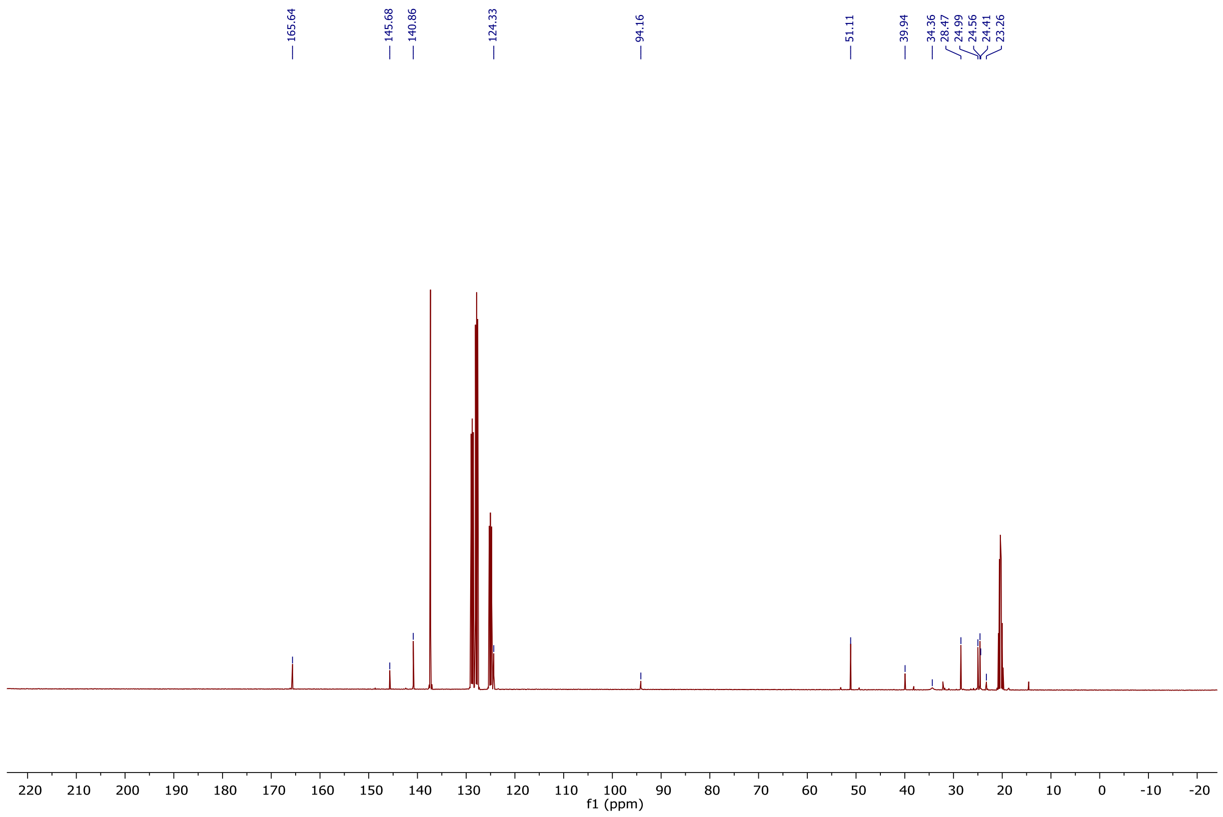

Figure S12. *$^{13}C\{^{1}H\}$ NMR Spectrum (C_7D_8, 233 K, 100.62 MHz) for* ***[(BDI)Ca(TMP)] (7)***.

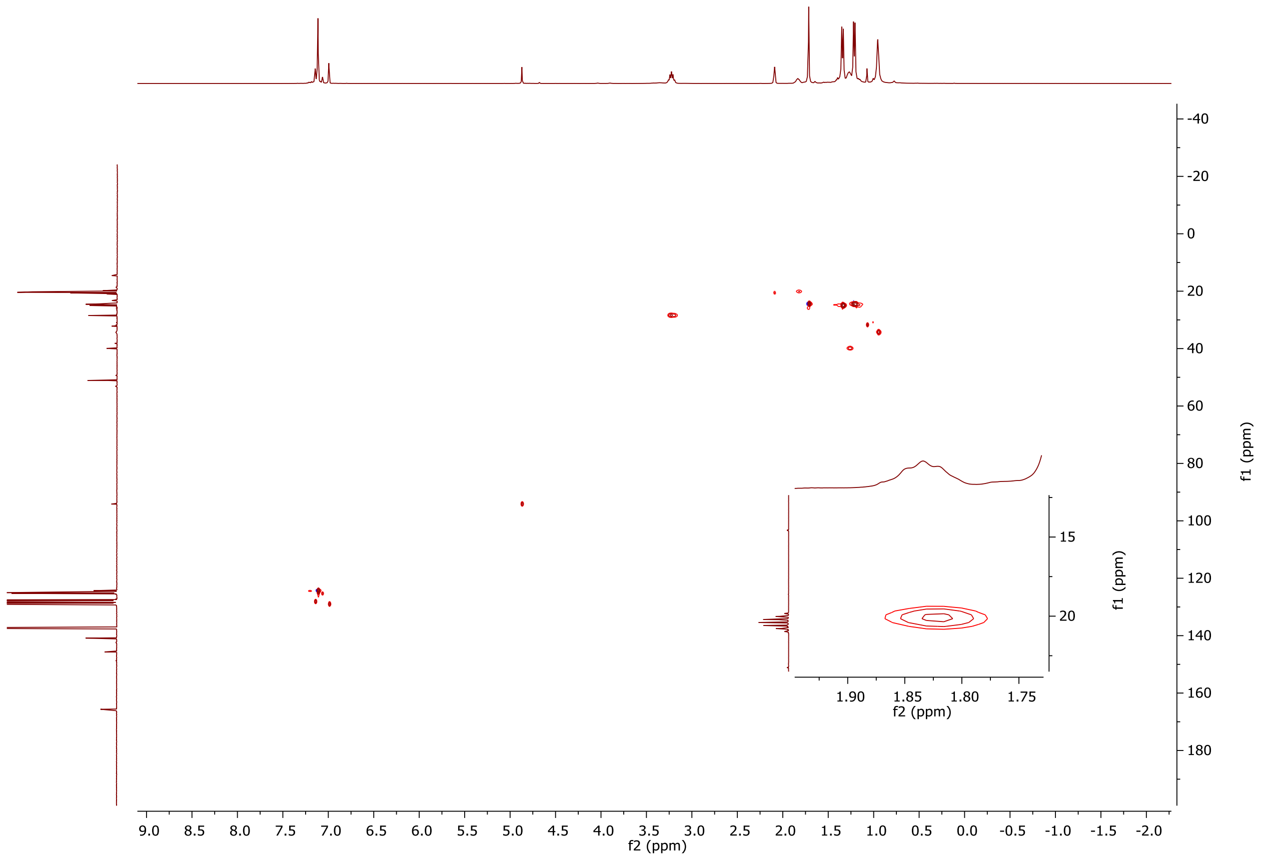

Figure S13. *^{1}H-^{13}C HSQC trace (C_7D_8, 233 K, 400.13, 100.62 MHz) for* ***[(BDI)Ca(TMP)] (7)***.

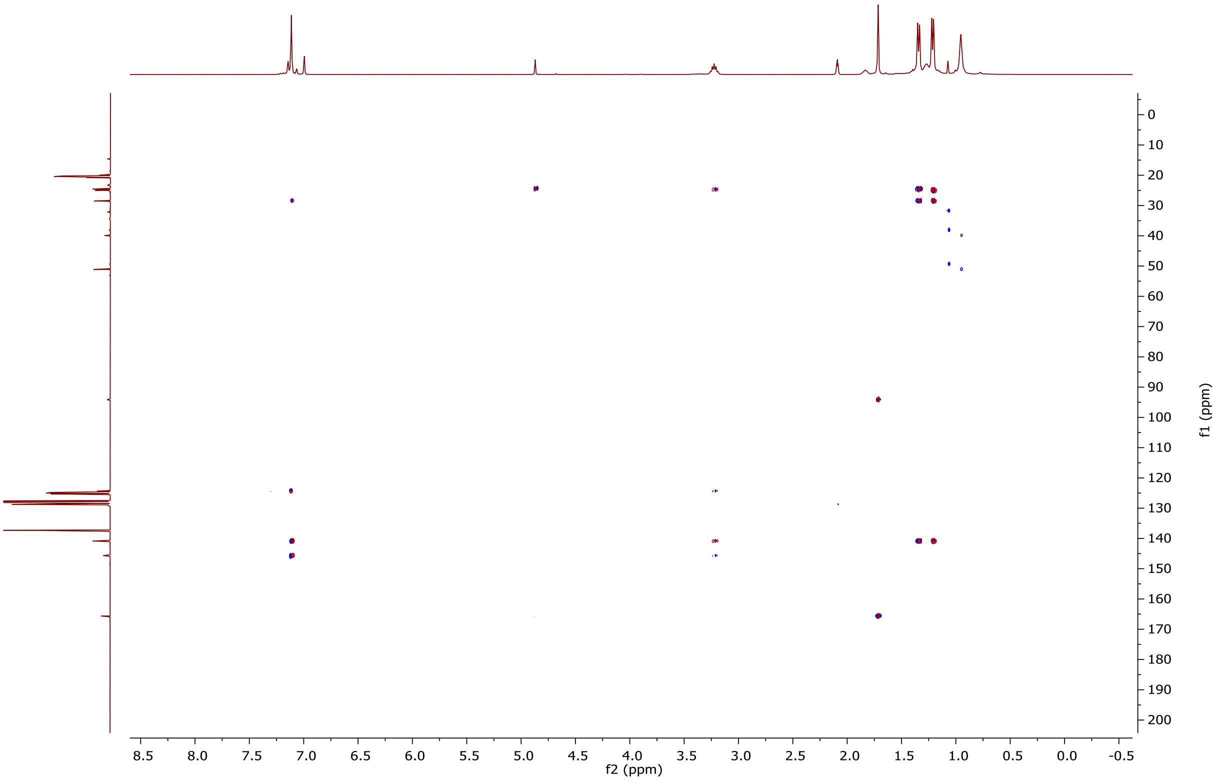

Figure S14. *^{1}H-^{13}C HMBC trace (C_7D_8, 233 K, 400.13, 100.62 MHz) for* ***[(BDI)Ca(TMP)] (7)****.*

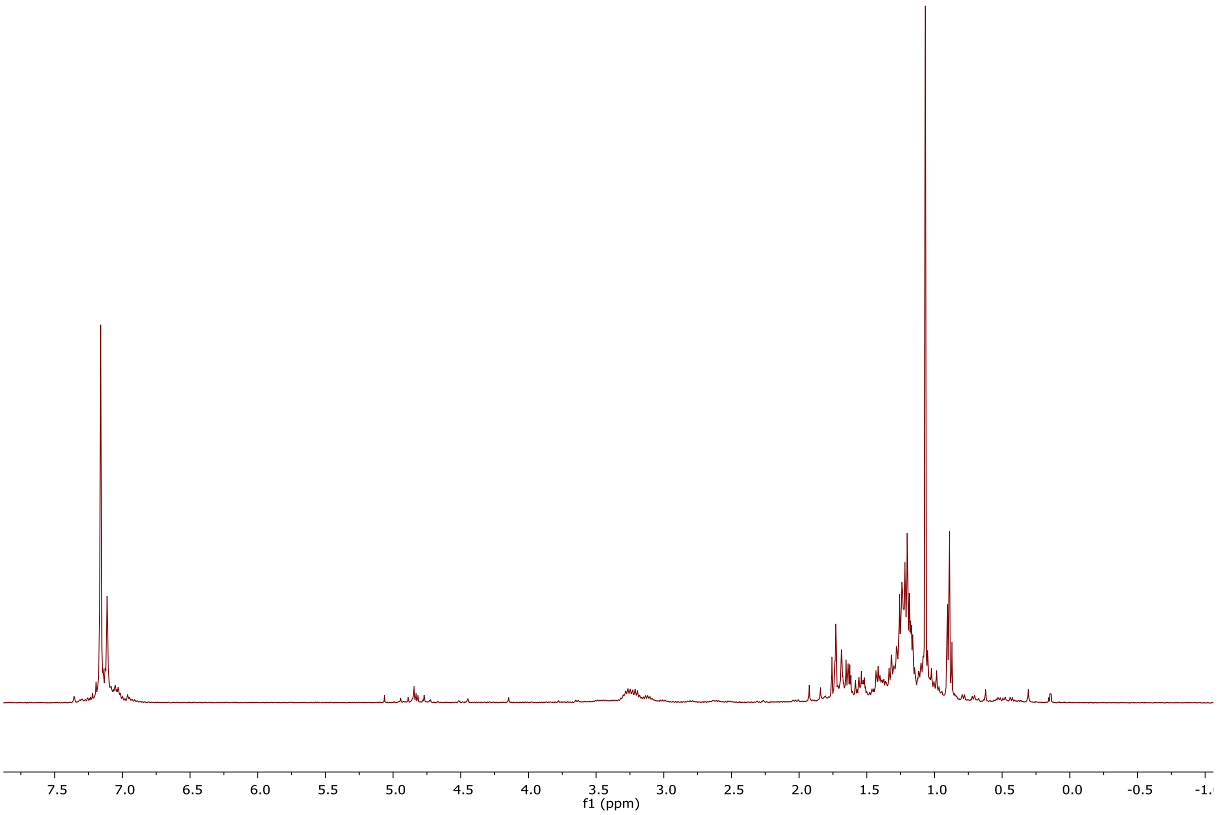

Figure S15. *^{1}H NMR Spectrum (C_6D_6, 298 K, 400.13 MHz) from dissolving* ***[(BDI)Ca(TMP)]*** *in C_6D_6 at room-temperature.*

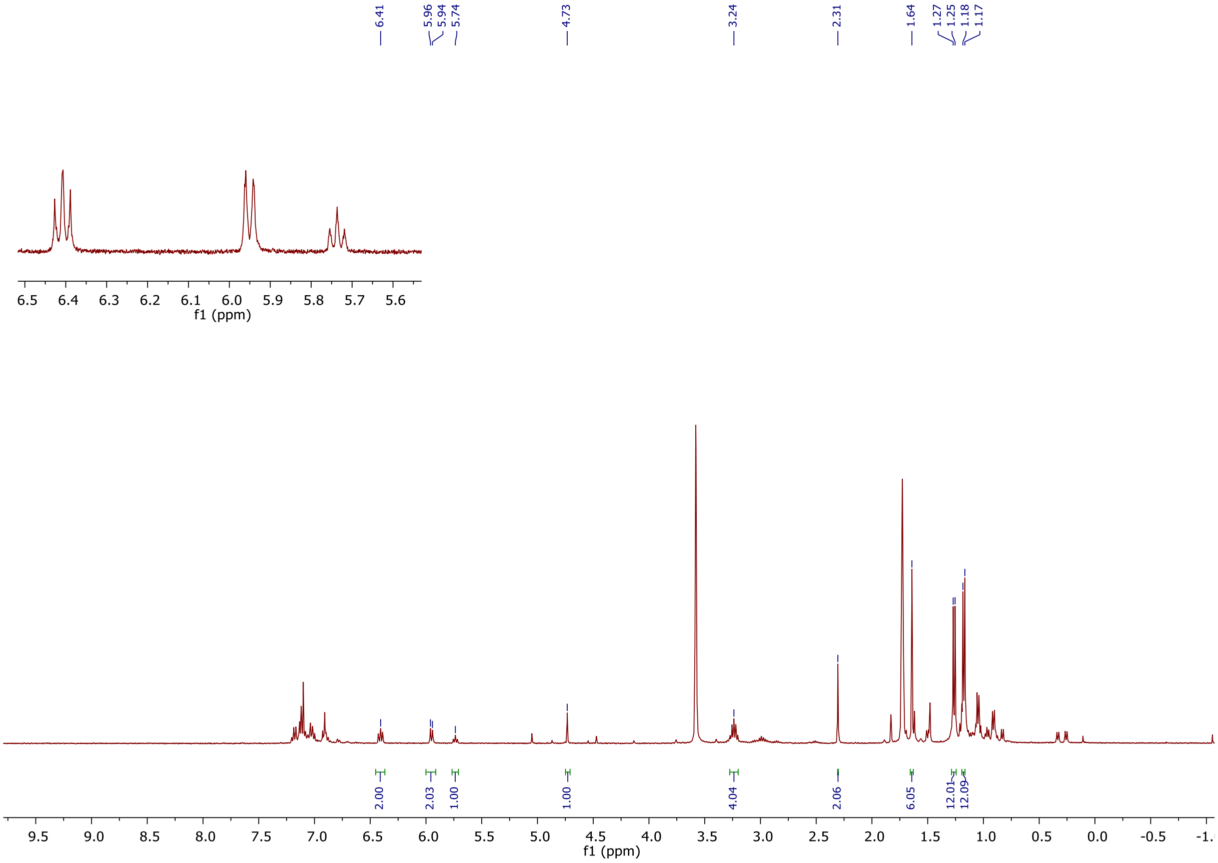

Figure S16. *^{1}H NMR Spectrum (C_4D_8O, 298 K, 400.13 MHz) for* ***[(BDI)Ca($CH_2C_6H_5$)] (8)***.

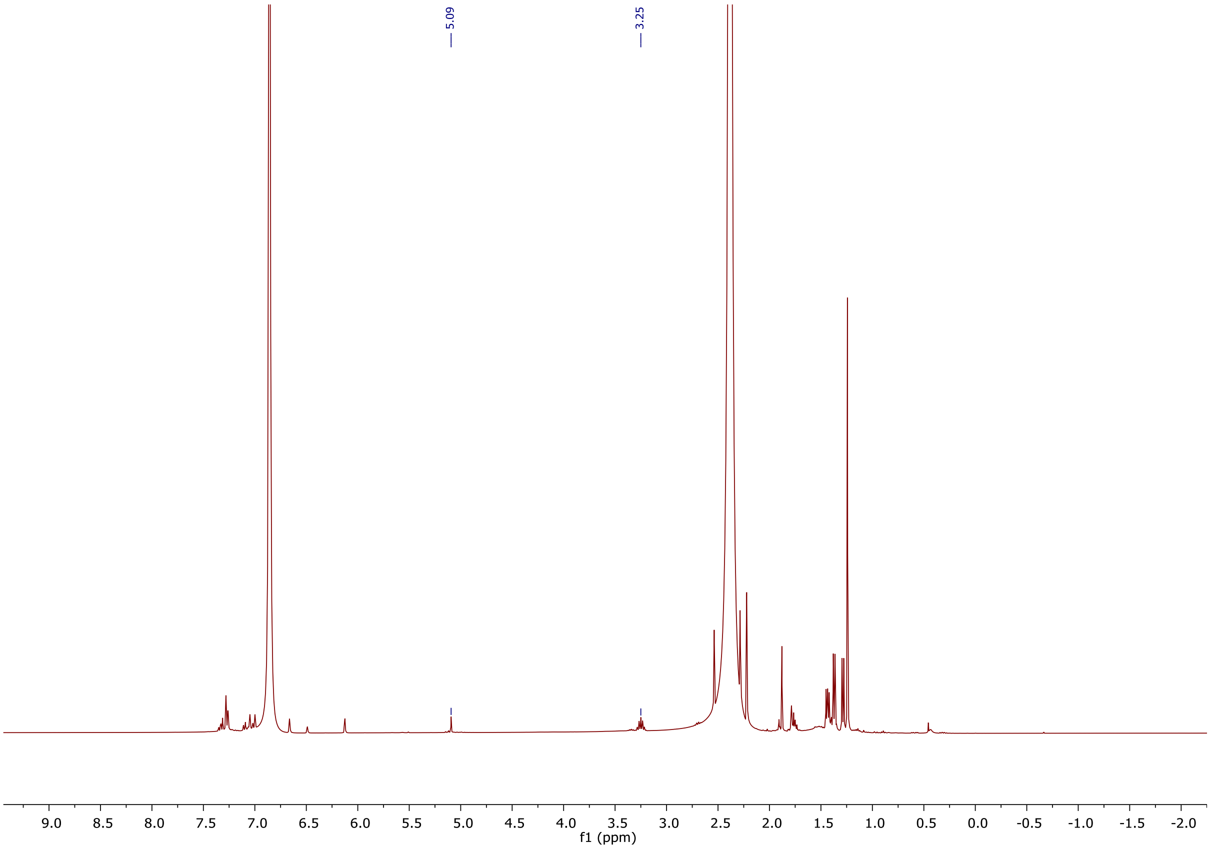

Figure S17. *^{1}H NMR Spectrum (protio-1,3-^{t}BuPh, 298 K, 400.13 MHz) for in-situ generated* ***[(BDI)Zn(3,5-^{t}Bu)Ph] (6)***.

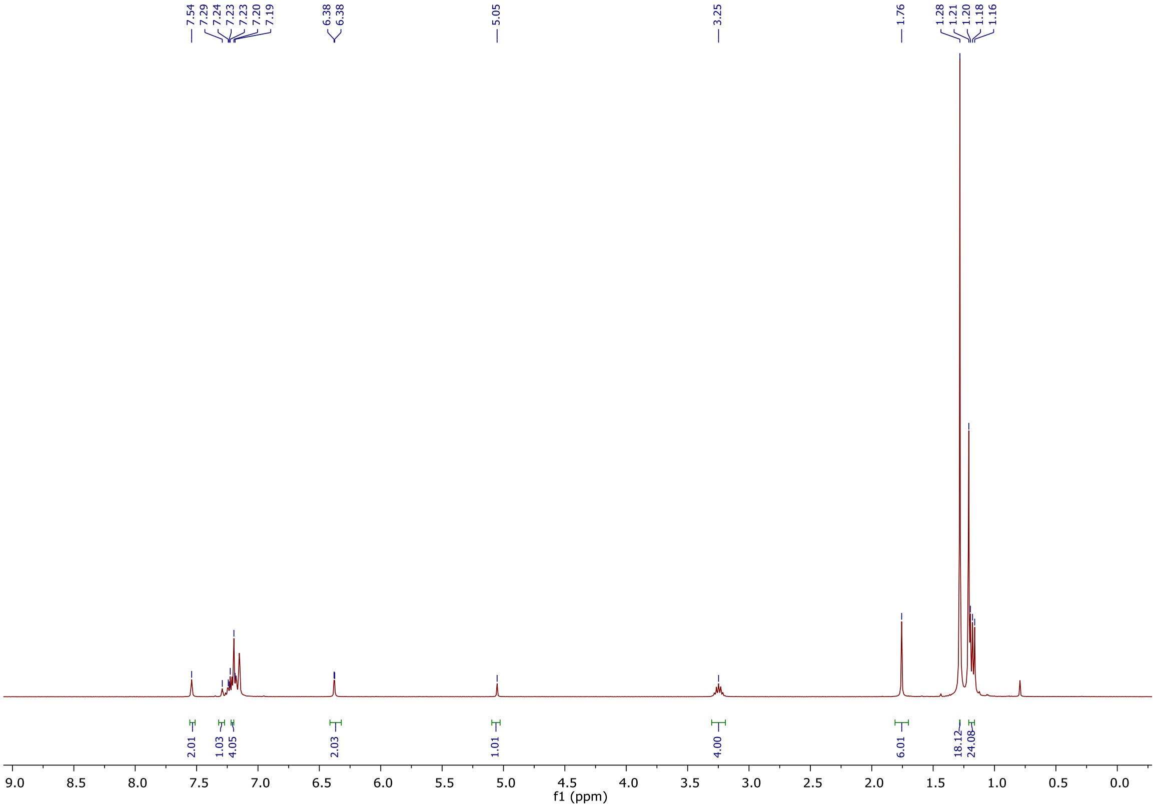

Figure S18. *^{1}H NMR Spectrum (C_6D_6, 298 K, 400.13 MHz) for* ***[(BDI)Zn(3,5-^{t}Bu)Ph] (6)***.

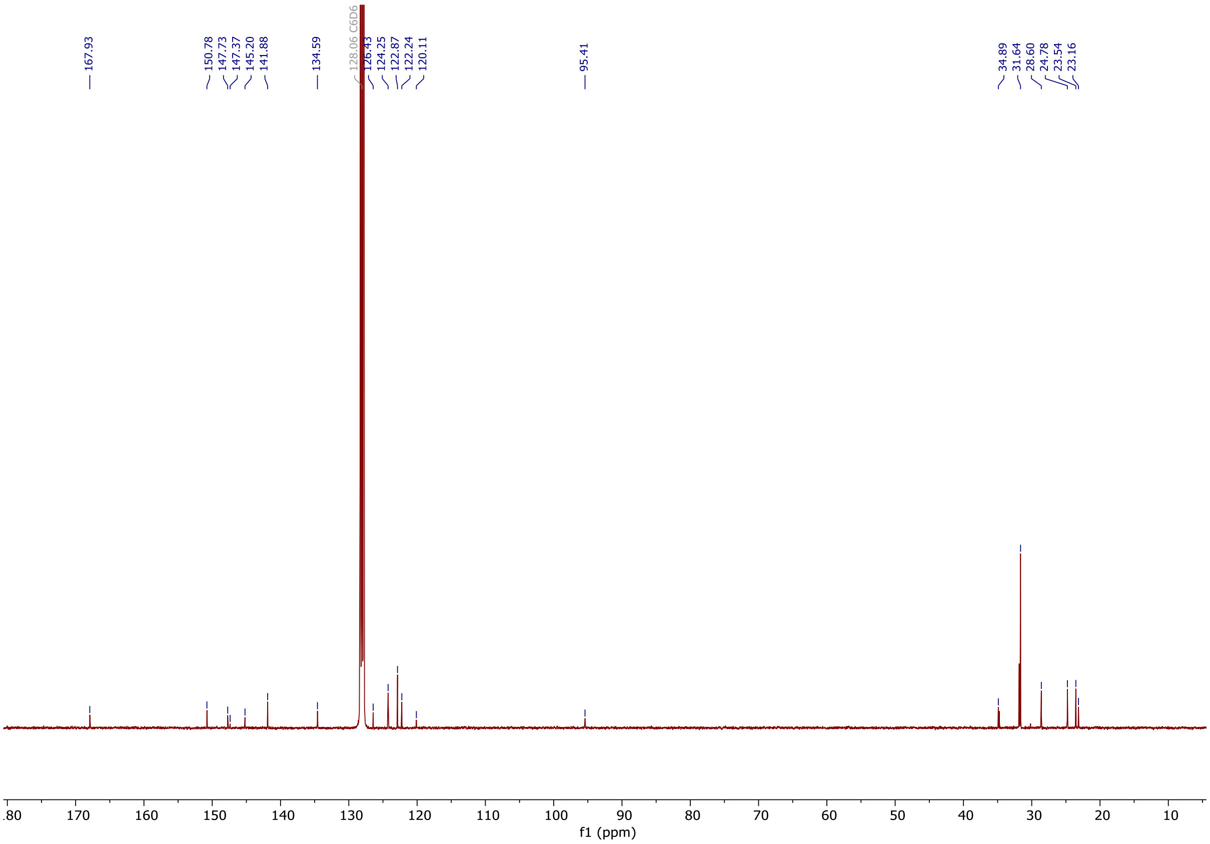

Figure S19. *$^{13}C\{^1H\}$ NMR Spectrum (C_6D_6, 298 K, 100.62 MHz) for* ***[(BDI)Zn(3,5-^{t}Bu)Ph] (6)***.

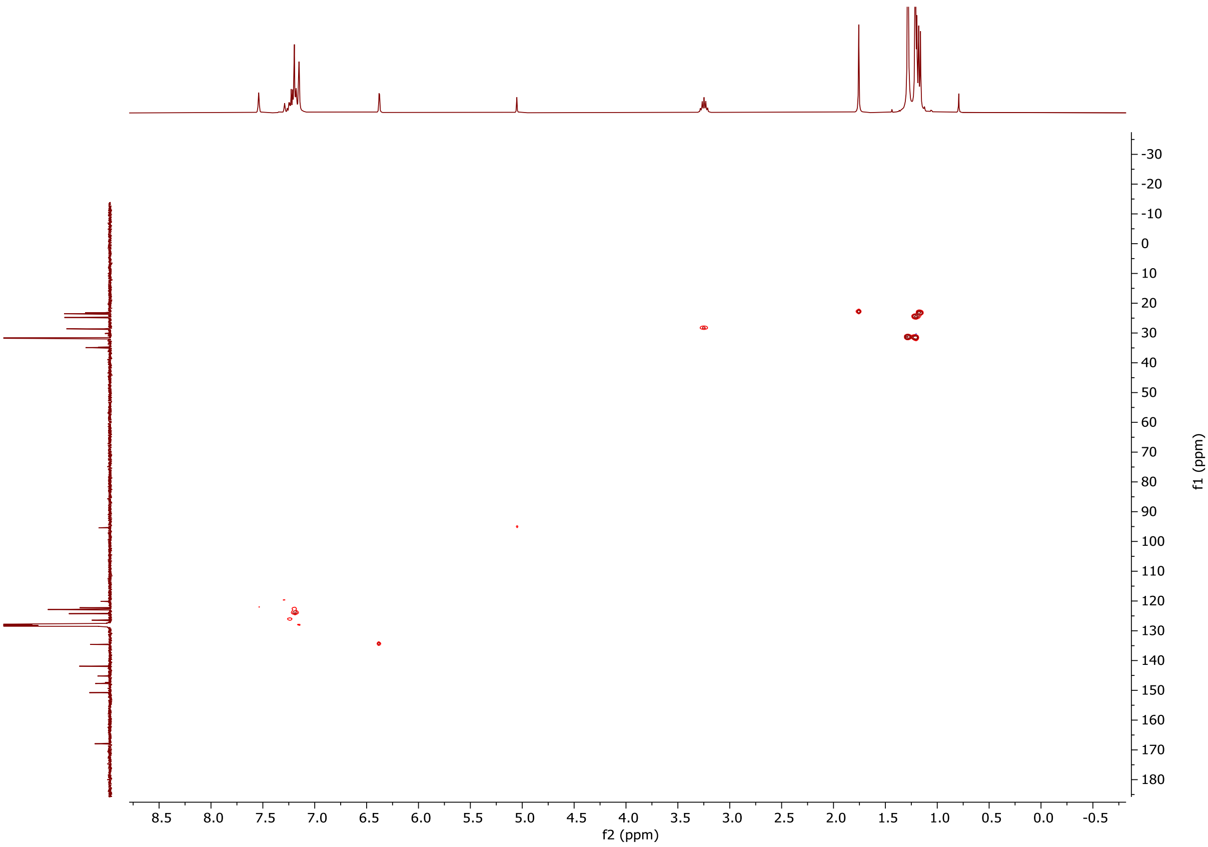

Figure S20. *^{1}H-^{13}C HSQC trace (C_6D_6, 298 K, 400.13, 100.62 MHz) for* ***[(BDI)Zn(3,5-[t]Bu)Ph] (6)***.

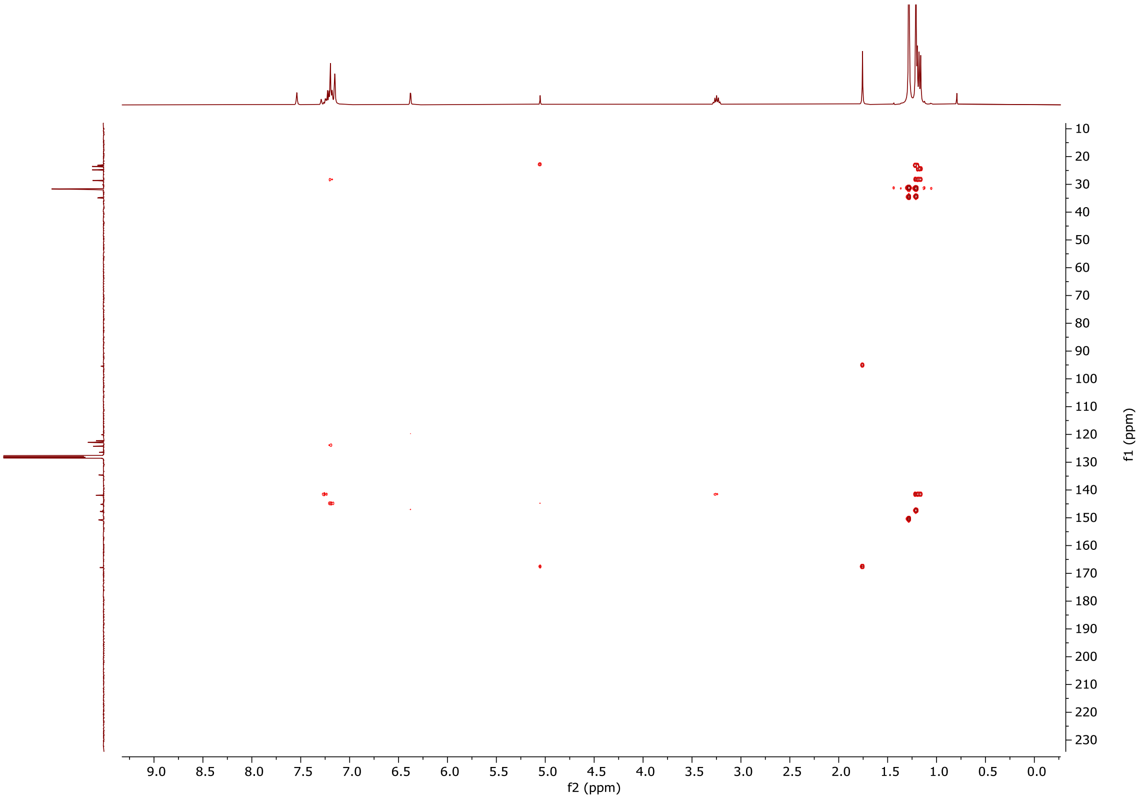

Figure S21. *^{1}H-^{13}C HMBC trace (C_6D_6, 298 K, 400.13, 100.62 MHz) for* ***[(BDI)Zn(3,5-[t]Bu)Ph] (6)***

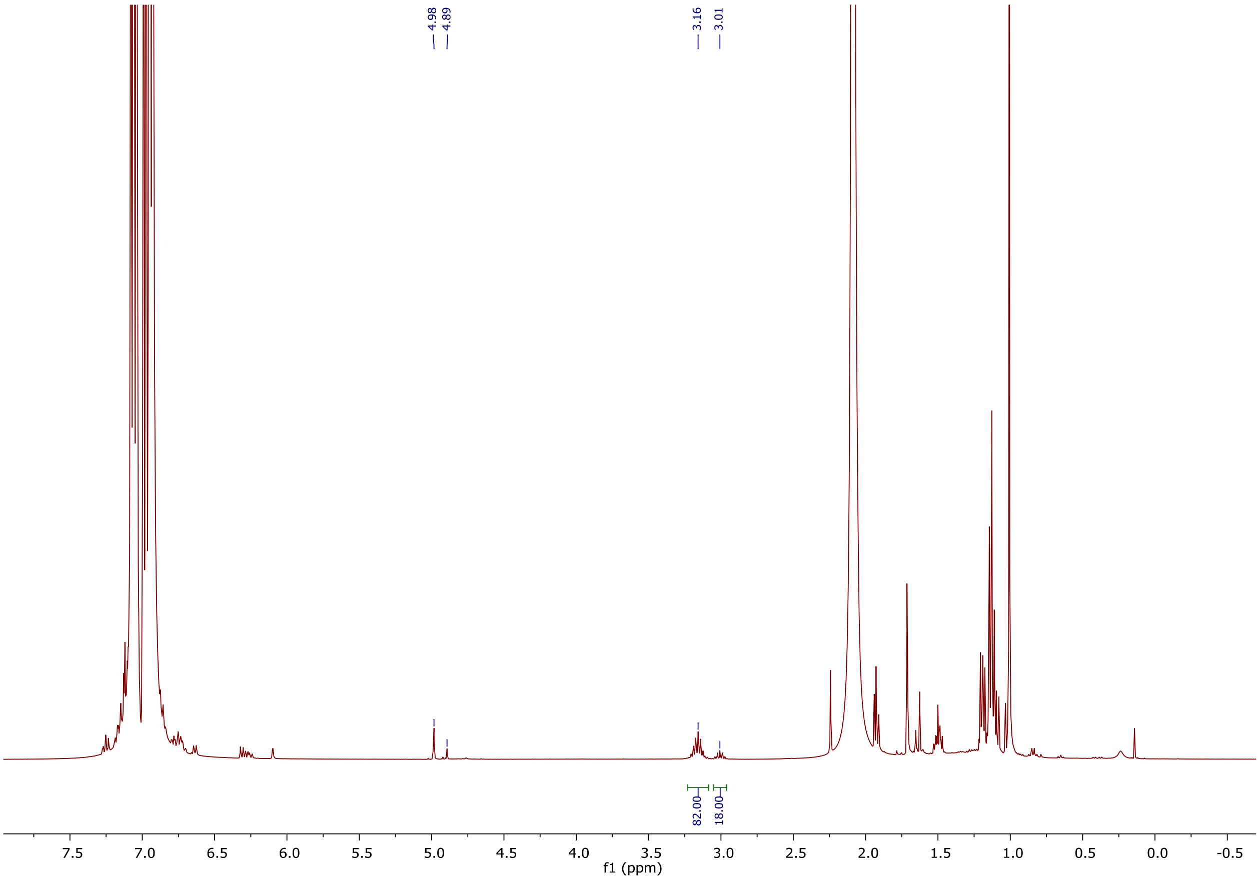

Figure S22. [1]*H NMR Spectrum (protio-toluene, 298 K, 400.13 MHz) for in-situ generated* ***[(BDI)Zn-3-MePh] (4^m)*** *and* ***[(BDI)Zn-4-MePh] (4^m).***

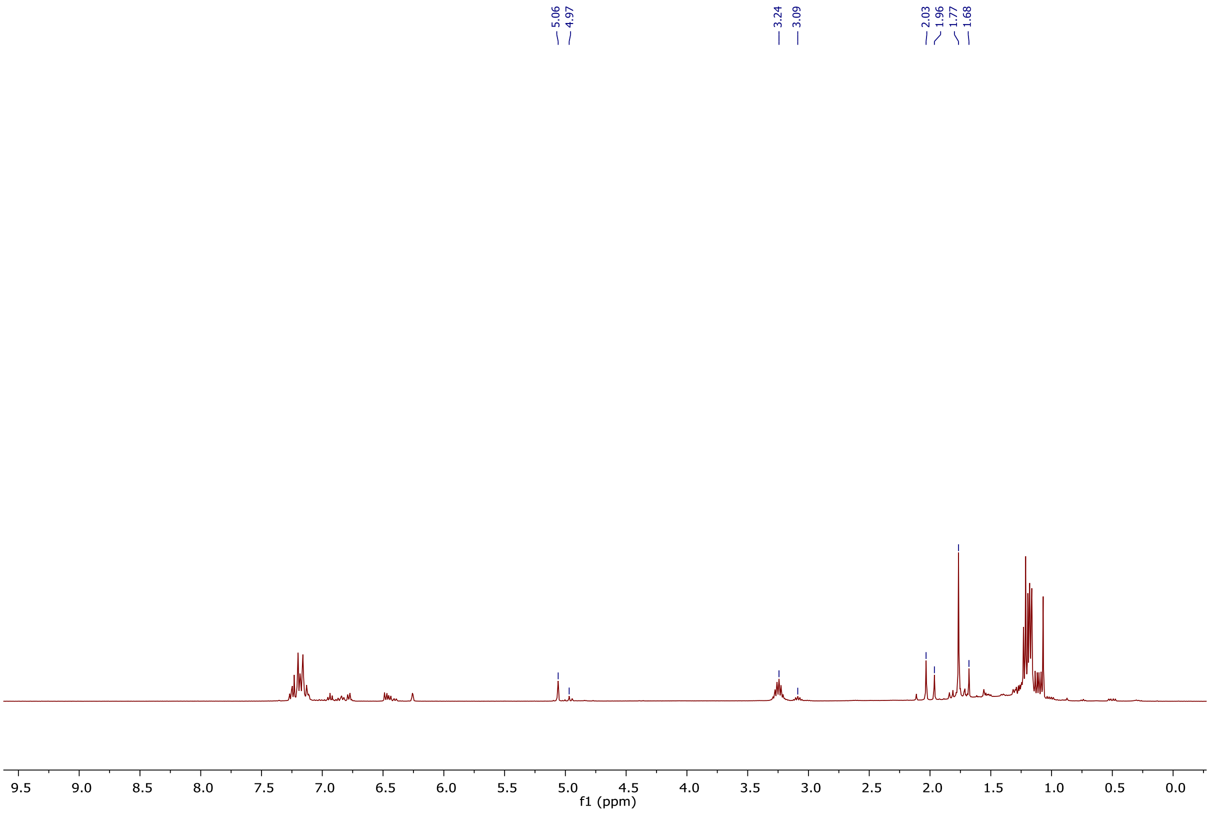

Figure S23. [1]*H NMR Spectrum (C_6D_6, 298 K, 400.13 MHz) for* ***[(BDI)Zn-3-MePh] (4^m)*** *and* ***[(BDI)Zn-4-MePh] (4^p).***

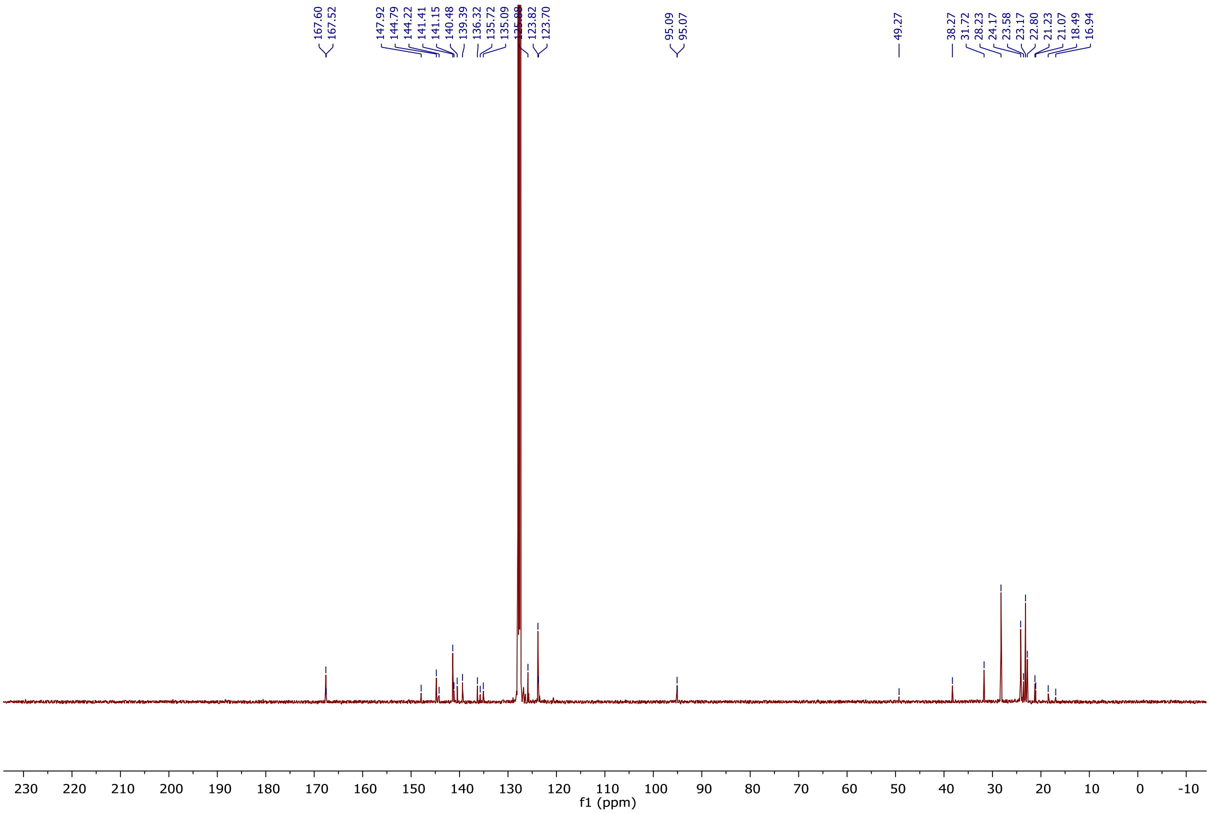

Figure S24. *^{13}C{^{1}H} NMR Spectrum (C_6D_6, 298 K, 400.13 MHz) for **[(BDI)Zn-3-MePh] (4^m)** and **[(BDI)Zn-4-MePh] (4^p).***

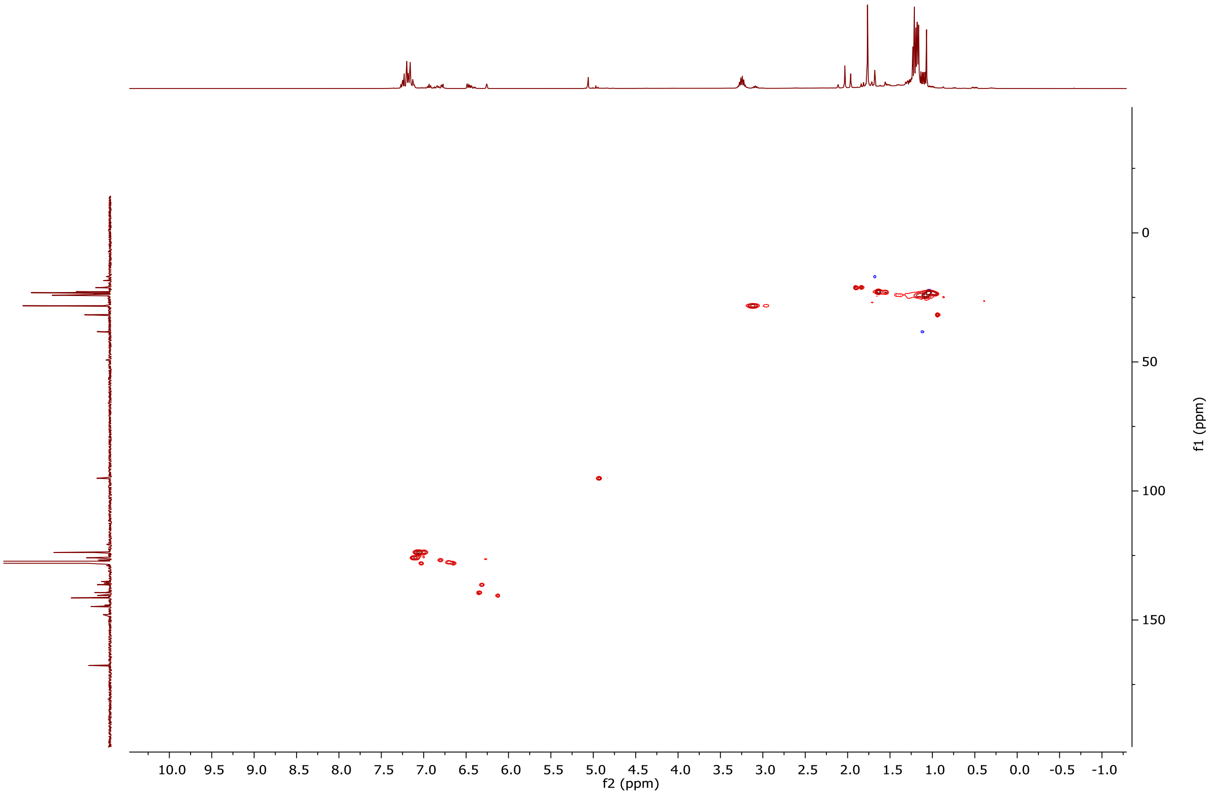

Figure S25**. ^{1}H-^{13}C HSQC trace (C_6D_6, 298 K, 400.13, 100.62 MHz) for **[(BDI)Zn-3-MePh] (4^m)** and **[(BDI)Zn-4-MePh] (4^p).

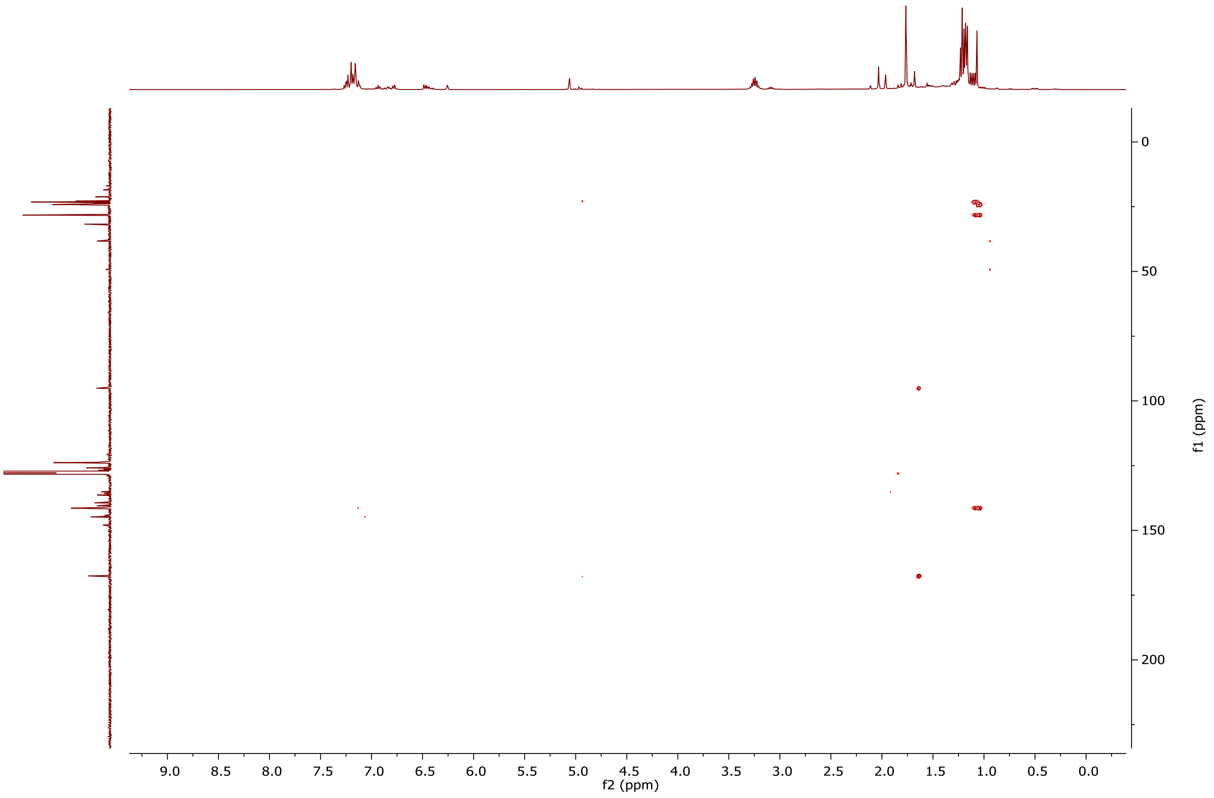

Figure S26. *^{1}H-^{13}C HMBC trace (C_6D_6, 298 K, 400.13, 100.62 MHz) for* ***[(BDI)Zn-3-MePh] (4ᵐ)*** *and* ***[(BDI)Zn-4-MePh] (4ᵖ).***

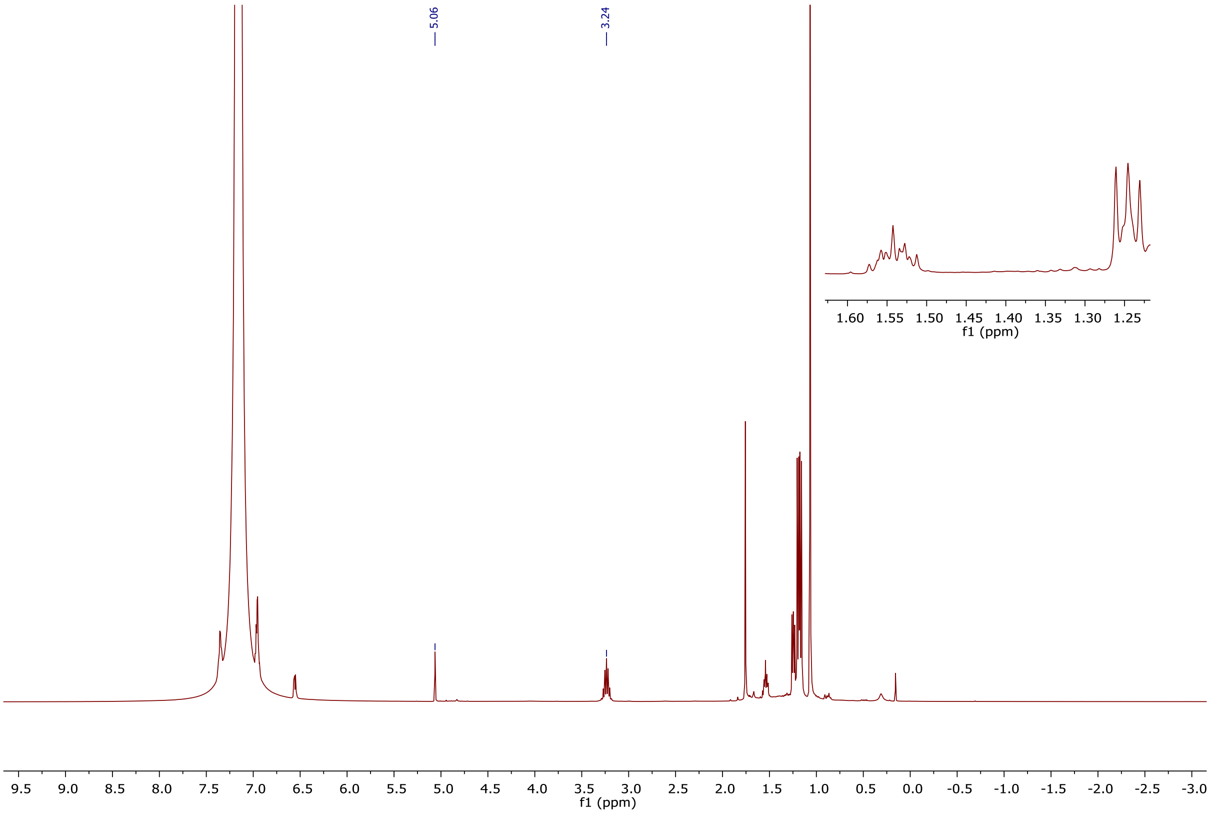

Figure S27. *^{1}H NMR Spectrum (C_6H_6, 298 K, 400.13 MHz) for in-situ generated* ***[(BDI)ZnPh] (2)***.

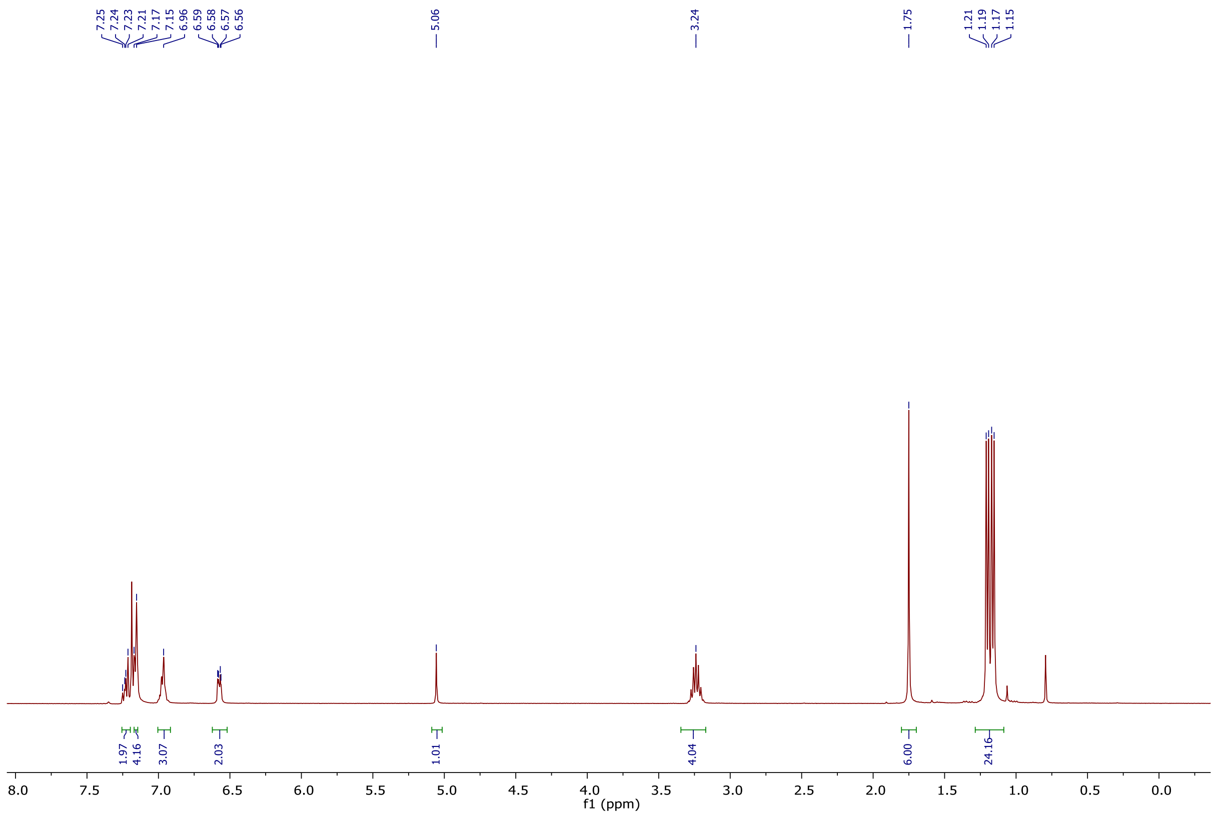

Figure S28. *^{1}H NMR Spectrum (C_6D_6, 298 K, 400.13 MHz) for* ***[(BDI)ZnPh] (2)***.

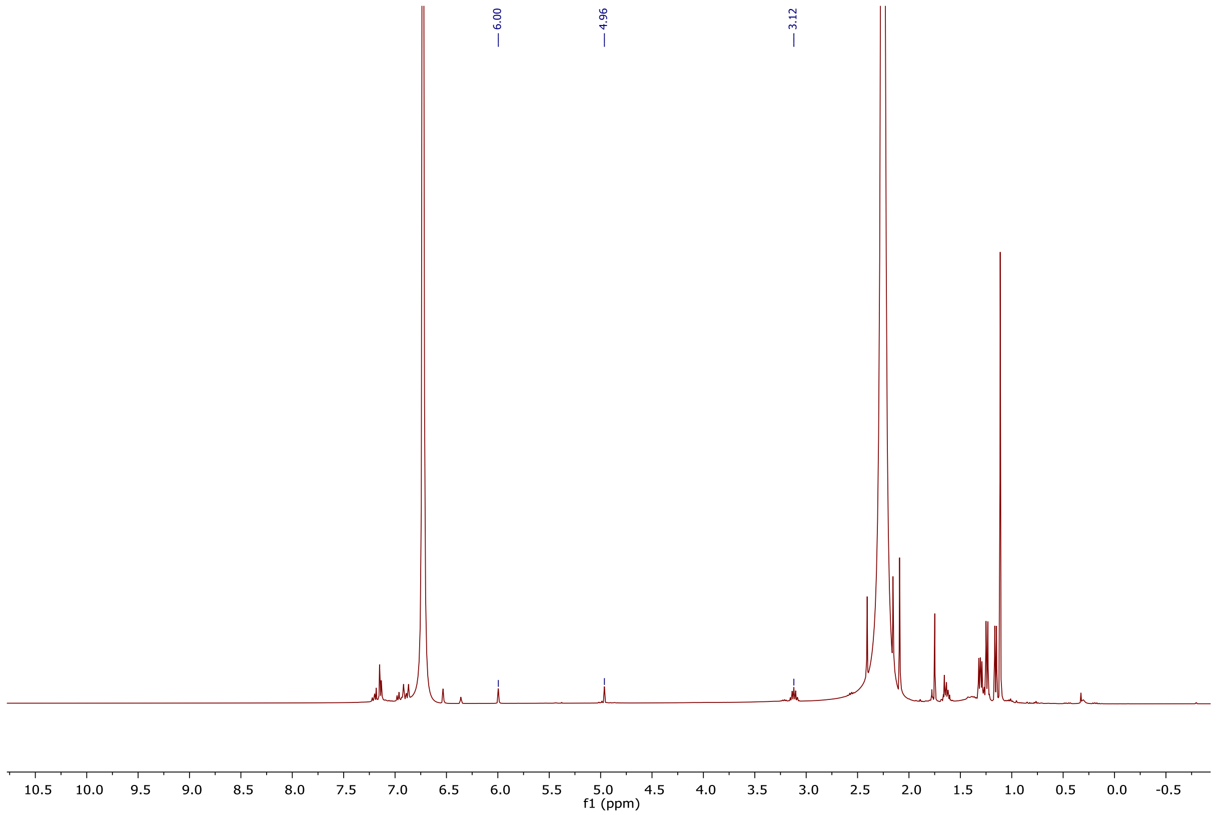

Figure S29. *^{1}H NMR Spectrum (mesitylene, 298 K, 400.13 MHz) for in-situ generated* ***[(BDI)Zn-CH$_2$-Ph-3,5-Me] (5)***.

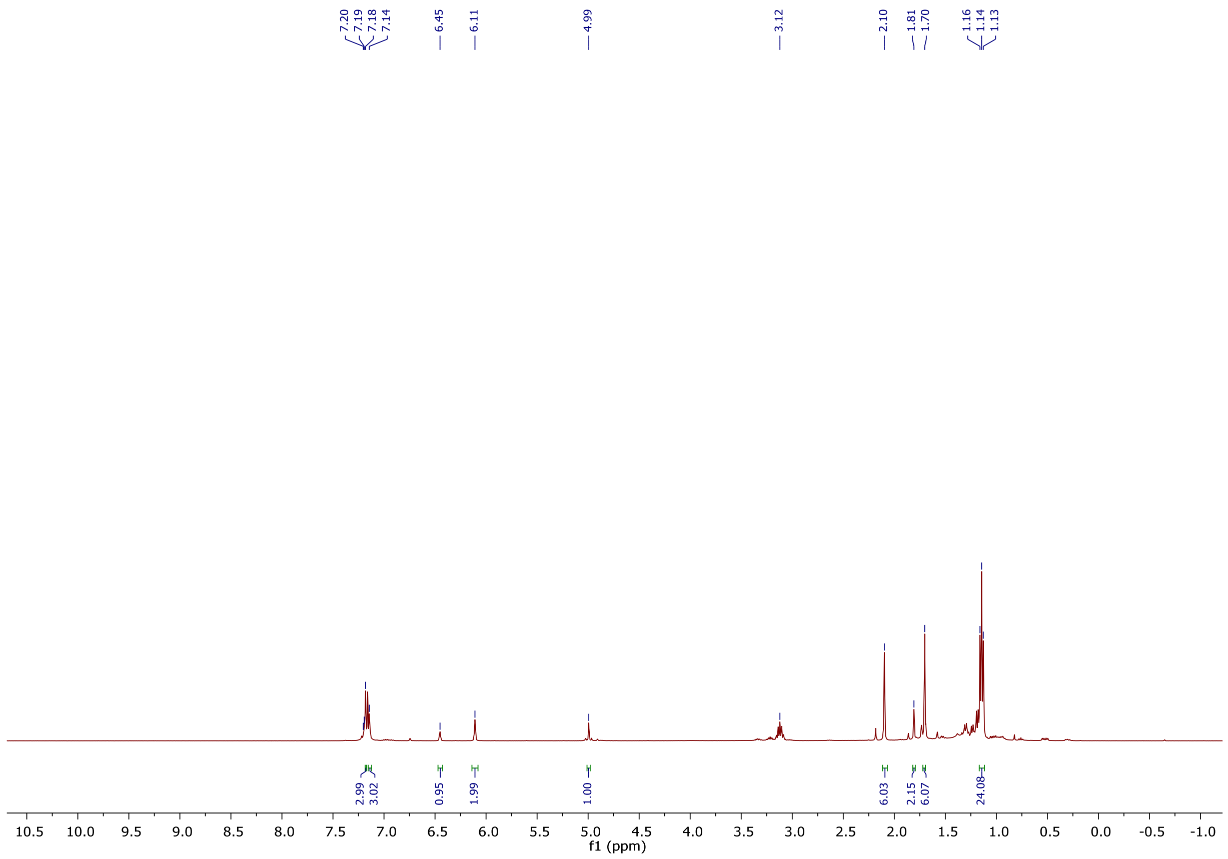

Figure S30. *^{1}H NMR Spectrum (C_6D_6, 298 K, 400.13 MHz) for* ***[(BDI)Zn-CH$_2$-Ph-3,5-Me] (5)***.

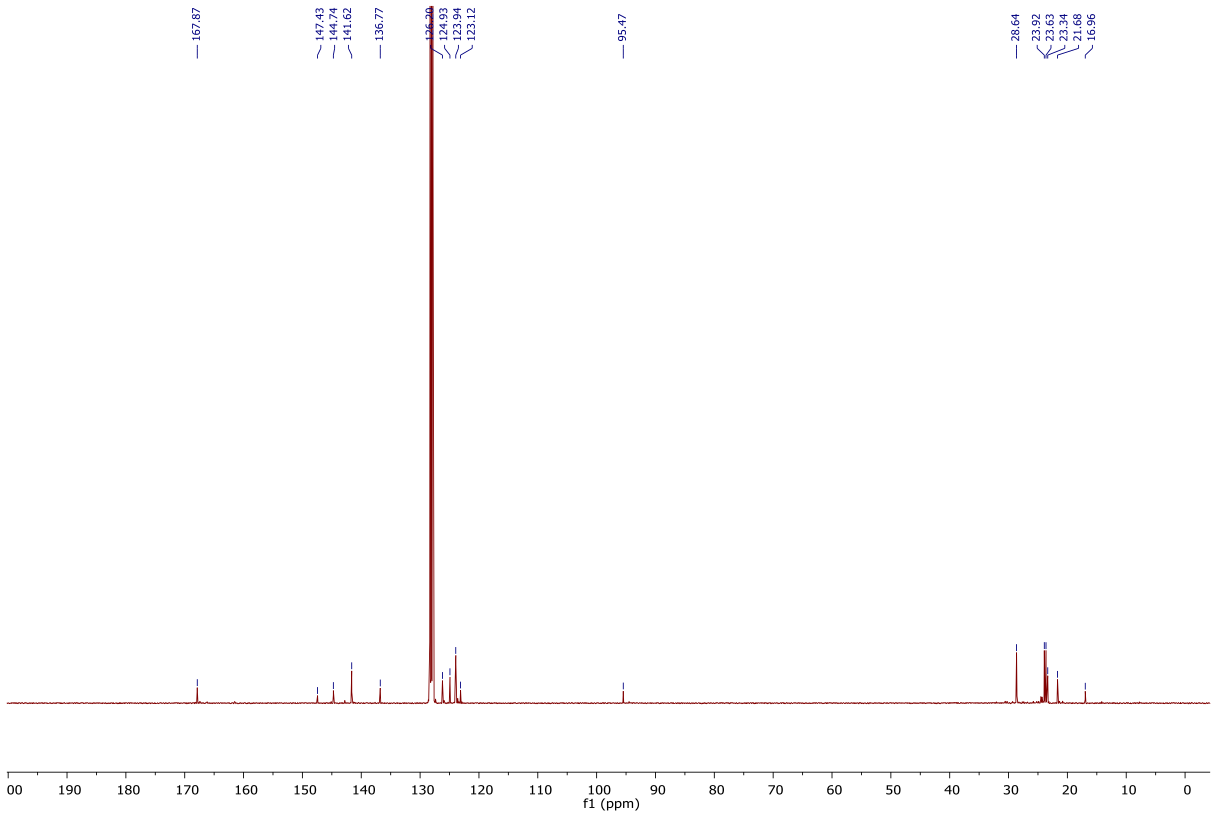

Figure S31. *^{13}C{^{1}H} NMR Spectrum (C_6D_6, 298 K, 400.13 MHz) for* ***[(BDI)Zn-CH$_2$-Ph-3,5-Me] (5)***.

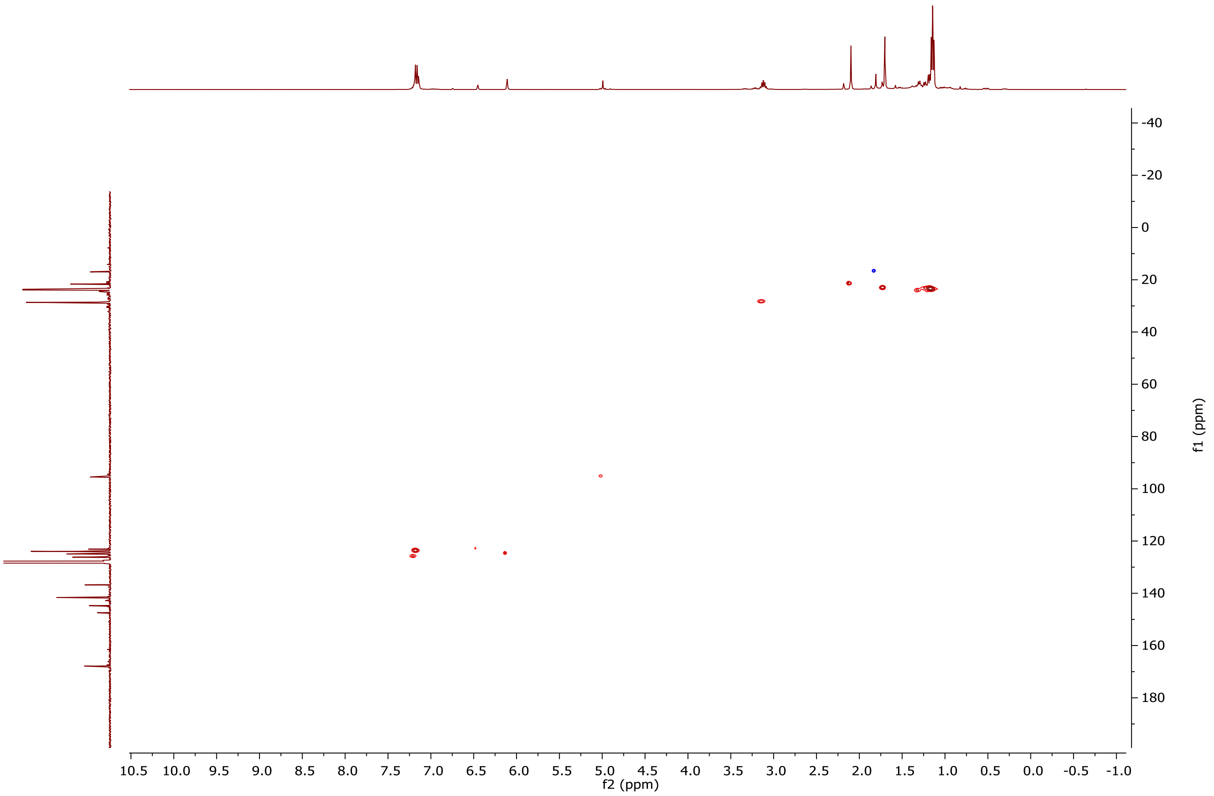

Figure S32. *^{1}H-^{13}C HSQC trace (C_6D_6, 298 K, 400.13, 100.62 MHz) for* ***[(BDI)Zn-CH$_2$-Ph-3,5-Me] (5).***

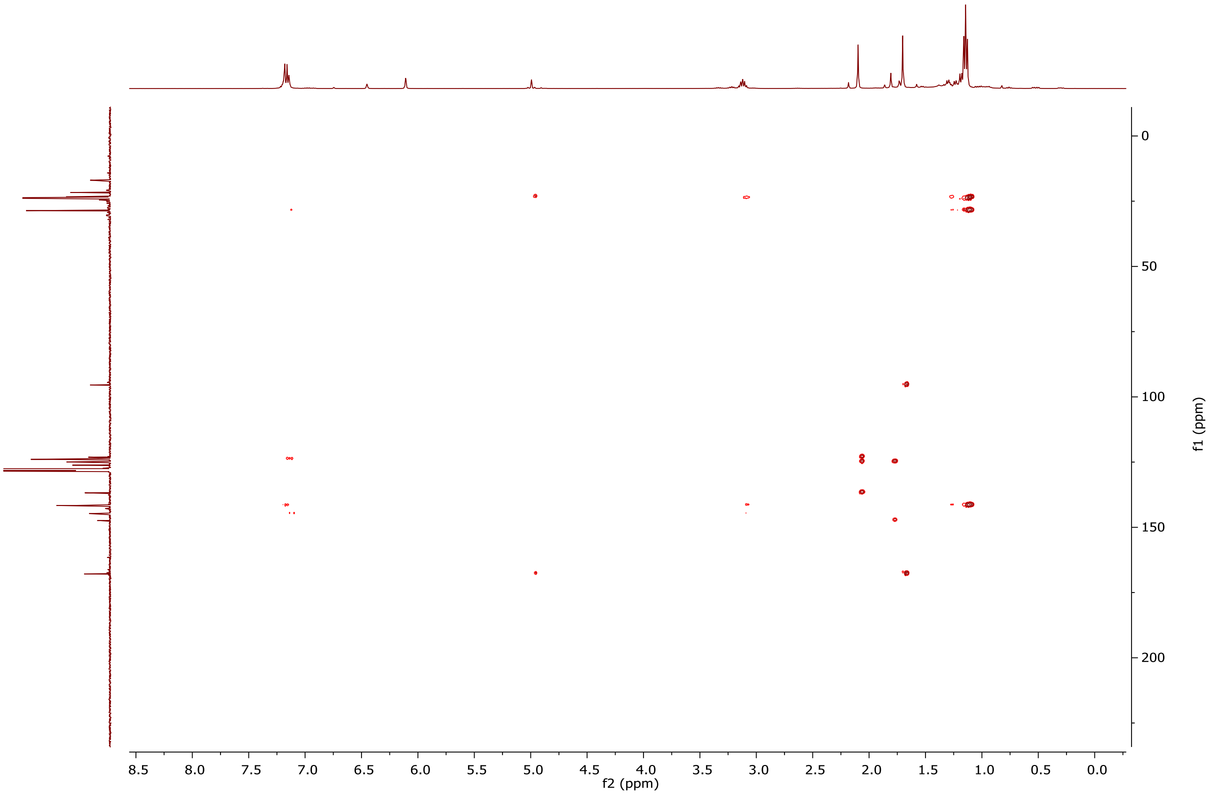

Figure S33. *^{1}H-^{13}C HMBC trace (C_6D_6, 298 K, 400.13, 100.62 MHz) for* ***[(BDI)Zn-CH$_2$-Ph-3,5-Me] (5)****.*

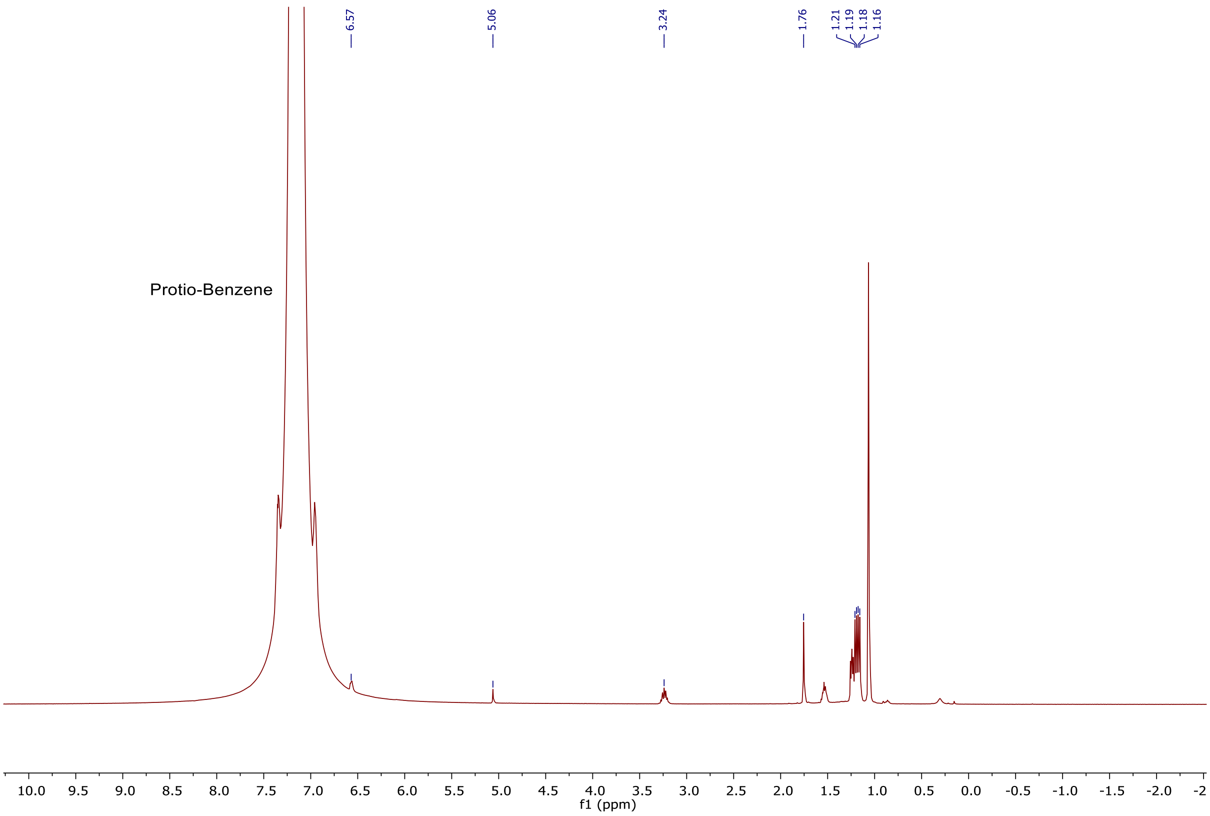

Figure S34. *^{1}H NMR Spectrum (C_6H_6, 298 K, 400.13 MHz) for in-situ generated* ***[(BDI)ZnPh] (2)*** *for telescoped Negishi coupling.*

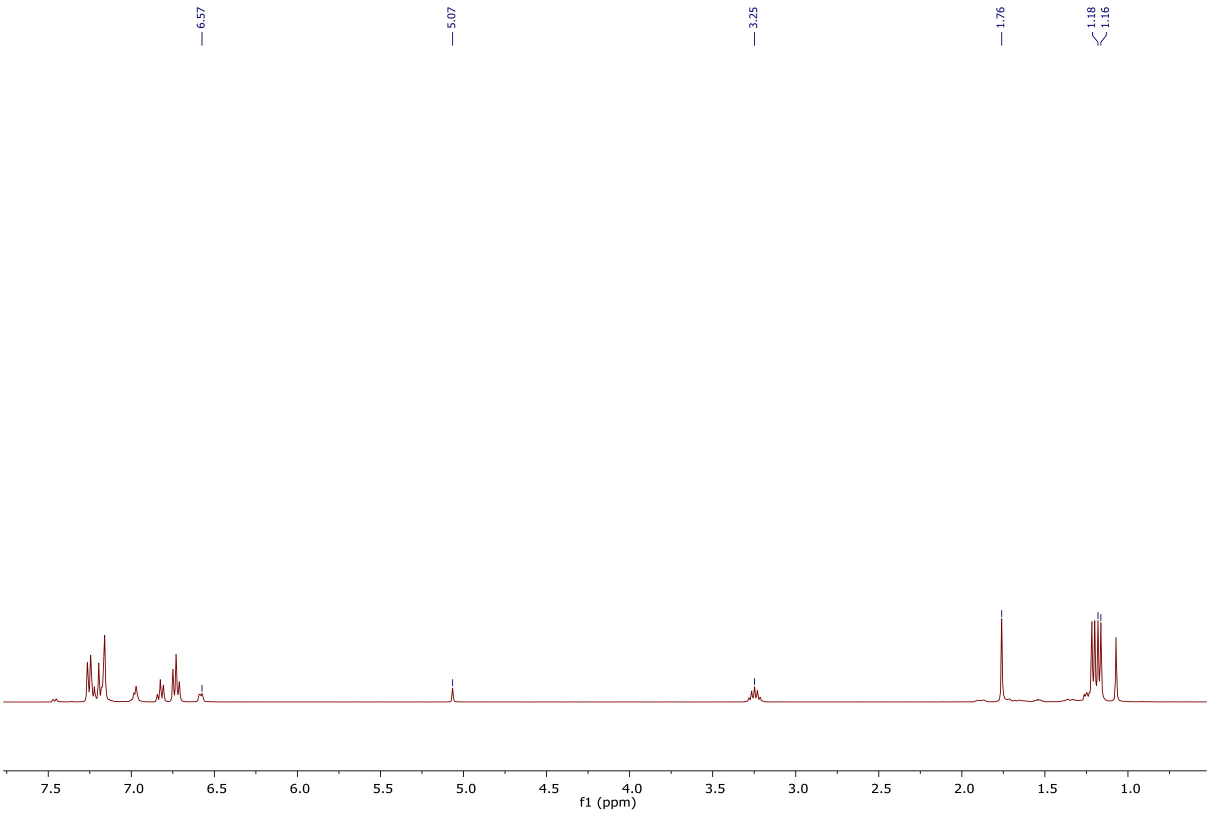

Figure S35*. ^{1}H NMR Spectrum (C_6D_6, 298 K, 400.13 MHz) of reagents before heating the telescoped Negishi reaction.*

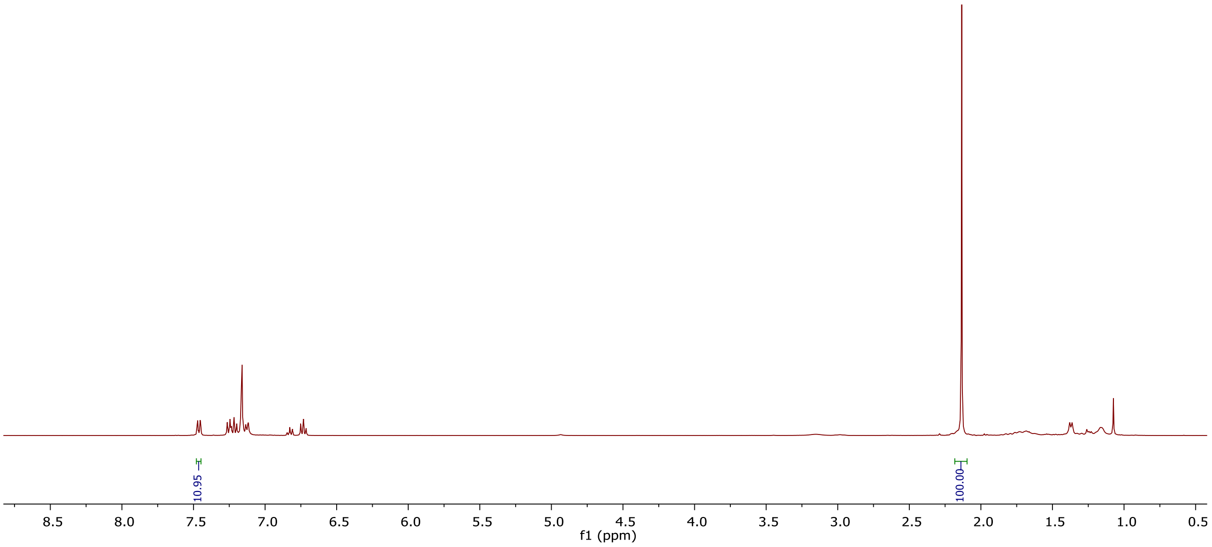

Figure S36. *^{1}H NMR Spectrum (C_6H_6, 298 K, 400.13 MHz) for telescoped Negishi reaction, demonstrating Biphenyl formation and hexamethylbenzene.*

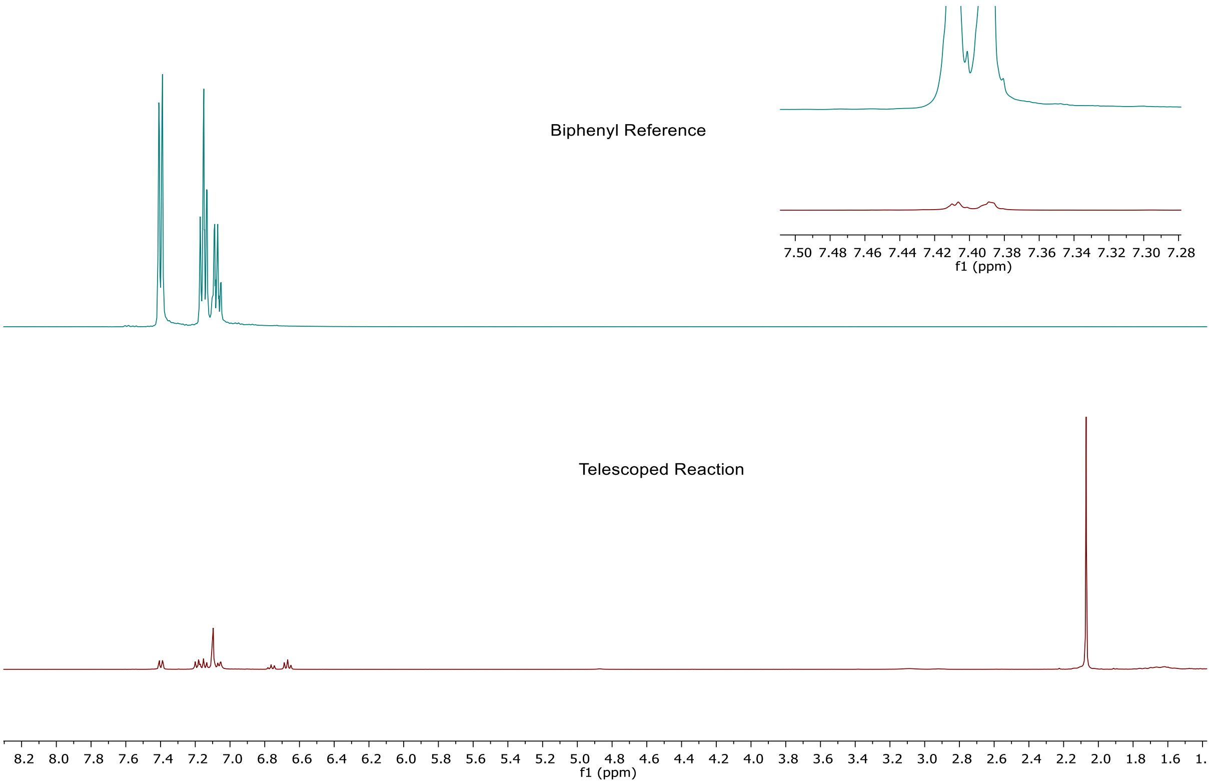

Figure S37. *^{1}H NMR Stacked Spectra (C_6H_6, 298 K, 400.13 MHz) for telescoped Negishi reaction overlayed with Biphenyl reference spectrum with expanded biphenyl peak.*

Crystallographic Data

Single Crystal X-ray diffraction data for compound **1** were collected on an Agilent Xcalibur diffractometer using Mo-Kα radiation (λ = 0.71073 Å), compounds **3**, ***4^p^***, **5** and **7** were collected on an Agilent SuperNova EosS2 diffractometer using Cu-Kα (1.54184 Å) radiation, whilst compounds **4^m** and **6** were collected on an XtaLAB Synergy, Dualflex, HyPix-Arc 100 diffractometer using Cu-Kα (1.54184 Å) radiation. The connectivity of compound **8** was confirmed by data collected at Diamond Light Source with the I19 Beamline, using silicon double crystal monochromated synchrotron radiation (λ = 0.6889 Å, Pilatus 2M detector). In each case, the crystals were maintained at 150 K during data collection. Using Olex2,[7] the structures were solved with the olex2.solve[8] structure solution program or ShelXT and refined with the ShelXL[9] refinement package using Least−Squares minimisation.

The asymmetric unit in **1** constitutes half of a dimer, the remainder of which is generated *via* the crystallographic, 2-fold rotation axis that is co-incident with Zn1. The hydride (H1), plus H2, were located and refined freely. Hydrogens attached to C29 and C35 were included at calculated positions but the U_{iso} values, therein, were allowed to refine freely as some of these atoms are implicated in contacts to Ca1. The refined U_{iso} quantities lend credibility to the positions of said hydrogens.

Half of a dimer plus two independent benzene halves (the latter disordered in a 65:35 ratio) comprise the asymmetric unit in compound **3**. All moieties are completed by virtue of space-group inversion symmetry. H1 and H2 were located and refined without restraints, while the hydrogens attached to C2, C36 and C38 were located and refined at a distance of 0.98 Å from the relevant parent atom. Distance and ADP restraints were employed, on merit, in the disordered solvent regions to assist convergence.

The asymmetric unit in **7** comprises one molecule of monomeric (BDI)CaTMP. Disorder prevailed on the TMP unit (N3;C30-C38), readily modelled as a 75:25 split over two sites. Appropriate distance and ADP restraints were implemented on the TMP unit to assist convergence. All hydrogens in the structure, were included at calculated positions, including those attached to C35.

The asymmetric unit in **6** constitutes half of a zinc-based complex (in which Zn1, C2, H2, C16, C19 and H19 are co-located with a crystallographic mirror plane) and half of a neutral 1,3-di-tert-butylbenzene species. All ring atoms in the latter, plus C30, C31 and C33 also reside on a mirror plane intrinsic to the space group. The electron-density pertaining to the C33-bound methyl groups is smeared, and this was modelled in the asymmetric unit as two disordered components for C34-36 (27% occupancy) and C34A-36A (23% occupancy). Distance and ADP restraints were employed, on merit, in the disordered region to assist convergence.

Two monomeric molecules are present in the asymmetric unit of **4*m***. Two of the Dipp-*iso*propyl CH_3 units (C64-C65) display positional disorder which is readily modelled in a 65:35 ratio. Appropriate distance and ADP restraints were employed to assist convergence.

The asymmetric unit for **4*p*** comprises half of the monomeric (BDI)Zn-4-MePh complex. The small size of the crystal led to weaker data and a higher R_{int} value. Disorder was identified on one of the Dipp iso-propyl units (C13-C15; 65:35 ratio) which was readily modelled. Appropriate distance and ADP restraints were employed in these disordered regions, to assist convergence.

The asymmetric unit in **5** comprises two independent monomeric molecules. Disorder was observed on one of the Dipp-*iso*propyl units (C53-55), which was readily modelled as a 50:50 split over two disordered sites. Appropriate distance and ADP restraints were employed to assist convergence.

Crystallographic data for all compounds have been deposited with the Cambridge Crystallographic Data Centre as supplementary publications CCDC 2430074-2430076 for **1**, **3** and **7**, respectively and 2465584-2465587 for **6**, **4*m***, **4*p*** and **5**, respectively. Copies of these data can be obtained free of charge on application to CCDC, 12 Union Road, Cambridge CB2 1EZ, UK [fax(+44) 1223 336033], e-mail: deposit@ccdc.cam.ac.uk.

Table S1. *Crystal Data and Structure Refinement for Compounds* ***1****,* ***3*** *and* ***7****.*

Identification code	e24msh13 (**1**)	y24msh01 (**3**)	y24msh17 (**7**)
Empirical formula	$C_{70}H_{120}Ca_2N_6Si_4Zn$	$C_{88}H_{134}Ca_2N_6Zn_2$	$C_{38}H_{59}CaN_3$
Formula weight	1303.60	1486.90	597.96
Crystal system	monoclinic	monoclinic	monoclinic
Space group	$C2/c$	$P2_1/n$	$P2_1/n$
a/ Å	21.3479(4)	14.3100(2)	9.01360(10)
b/ Å	11.4869(2)	18.1050(3)	20.42790(10)
c/ Å	31.6282(6)	16.3708(3)	19.5210(2)
α/ °	90	90	90
β/ °	100.777(2)	90.756(2)	90.9540(10)
γ/ °	90	90	90
U/ Å^3	7619.1(2)	4241.02(12)	3593.88(6)
Z	4	2	4
ρ_{calc}/ g cm^{-3}	1.136	1.164	1.105
μ/ mm^{-1}	0.561	2.085	1.700
F(000)	2832.0	1608.0	1312.0
Crystal size/ mm^3	0.543 × 0.261 × 0.217	0.105 × 0.029 × 0.029	0.17 × 0.16 × 0.07
2θ range for data collection/°	5.89 to 60.714	7.28 to 150.574	6.264 to 160.688
Index ranges	-29 ≤ h ≤ 30<br>-15 ≤ k ≤ 13<br>-40 ≤ l ≤ 43	-17 ≤ h ≤ 17<br>-17 ≤ k ≤ 22<br>-20 ≤ l ≤ 20	-11 ≤ h ≤ 11<br>-25 ≤ k ≤ 26<br>-24 ≤ l ≤ 22
Reflections collected	36648	43551	45635
Independent reflections, R_{int}	10056, 0.0329	8630, 0.0403	7707, 0.0297
Data/restraints/parameters	10056/0/413	8630/250/544	7707/363/487
Goodness–of–fit on F^2	1.037	1.037	1.044
Final R1, wR2 [I>=2σ(I)]	0.0392, 0.0850	0.0363, 0.0856	0.0402, 0.1049
Final R1, wR2 [all data]	0.0559, 0.0920	0.0448, 0.0896	0.0439, 0.1076
Largest diff. peak/hole/ e Å^{-3}	0.42/-0.38	0.47/-0.72	0.33/-0.36

Table S2. *Crystal Data and Structure Refinement for Compounds* ***4^m*, *4^p***

Identification code	s24msh61 (**4^m**)	y24msh34 (**4^p**)	y24msh27 (**5**)	s24msh39 (**6**)
Empirical formula	$C_{36}H_{48}N_2Zn$	$C_{36}H_{51}N_2Zn$	$C_{76}H_{104}N_4Zn_2$	$C_{57}H_{87}N_2Zn$
Formula weight	574.13	577.15	1204.37	865.65
Crystal system	monoclinic	orthorhombic	triclinic	monoclinic
Space group	$P2_1/c$	*Pnma*	*P*-1	$P2_1/m$
a/ Å	17.8171(6)	16.2048(10)	9.3864(2)	10.7608(2)
b/ Å	8.9039(2)	20.9090(8)	20.3547(3)	18.0935(3)
c/ Å	41.3857(13)	9.4511(6)	20.6455(3)	13.5593(2)
α/ °	90	90	63.852(2)	90
β/ °	97.154(3)	90	89.459(2)	93.810(2)
γ/ °	90	90	82.758(2)	90
U/ $Å^3$	6514.4(3)	3202.3(3)	3507.83(12)	2634.17(8)
Z	8	4	2	2
ρ_{calc}/ g cm^{-3}	1.171	1.197	1.140	1.091
μ/ mm^{-1}	1.216	1.237	1.150	0.897
F(000)	2464.0	1244.0	1296.0	946.0
Crystal size/ mm^3	0.13 × 0.06 × 0.04	0.06 × 0.04 × 0.03	0.22 × 0.17 × 0.08	0.108 × 0.07 × 0.055
2θ range for data collection/°	6.992 to 147.7	8.458 to 154.768	4.774 to 161.178	8.16 to 146.518
Index ranges	-22 ≤ h ≤ 21, -7 ≤ k ≤ 10, -51 ≤ l ≤ 51	-18 ≤ h ≤ 20, -21 ≤ k ≤ 26, -11 ≤ l ≤ 11	-11 ≤ h ≤ 11, -25 ≤ k ≤ 25, -24 ≤ l ≤ 26	-12 ≤ h ≤ 13, -14 ≤ k ≤ 22, -16 ≤ l ≤ 16
Reflections collected	86337	18340	44695	20302
Independent reflections, R_{int}	13000, 0.0994	3144, 0.1780	14782, 0.0315	5395, 0.0285
Data/restraints/parameters	13000/14/745	3144/33/222	14782/27/792	5395/66/347
Goodness–of–fit on F^2	1.167	1.048	1.019	1.061
Final *R*1, *wR*2 [*I*>=2σ(*I*)]	0.0858, 0.2043	0.0804, 0.2047	0.0476, 0.1281	0.0419, 0.1105
Final *R*1, *wR*2 [all data]	0.1060, 0.2150	0.1547, 0.2555	0.0563, 0.1338	0.0474, 0.1139
Largest diff. peak/hole/ e $Å^{-3}$	0.82/-0.66	0.50/-0.95	1.00/-0.56	0.63/-0.40

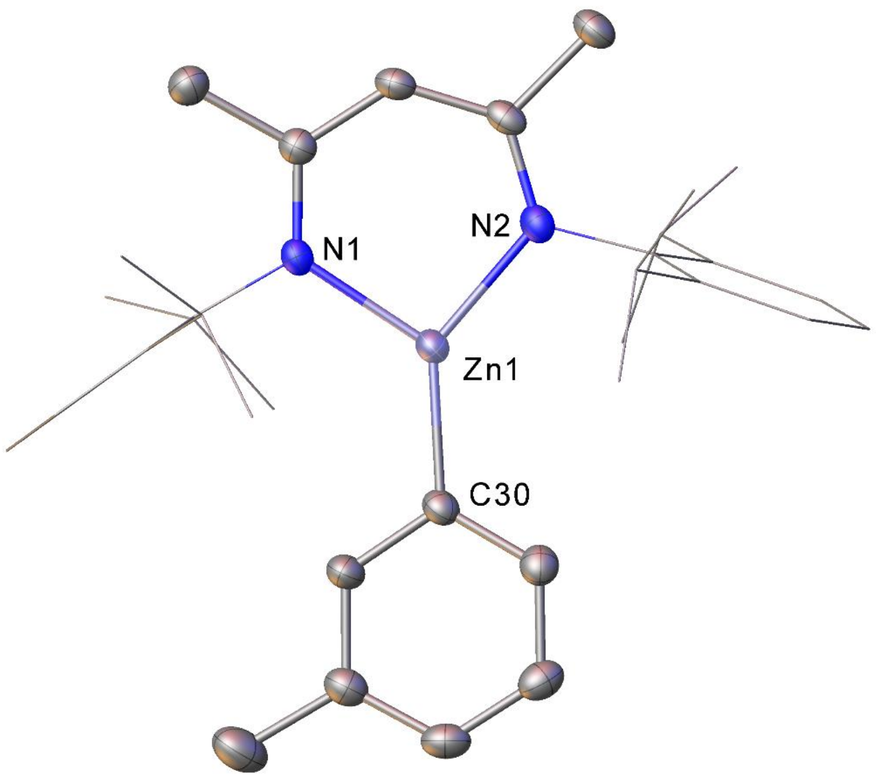

Figure S38. *Molecular structure of* ***4***^{m}*, displacement ellipsoids at 30%. For clarity, hydrogen atoms are omitted and the Dipp groups are displayed as wireframe.*

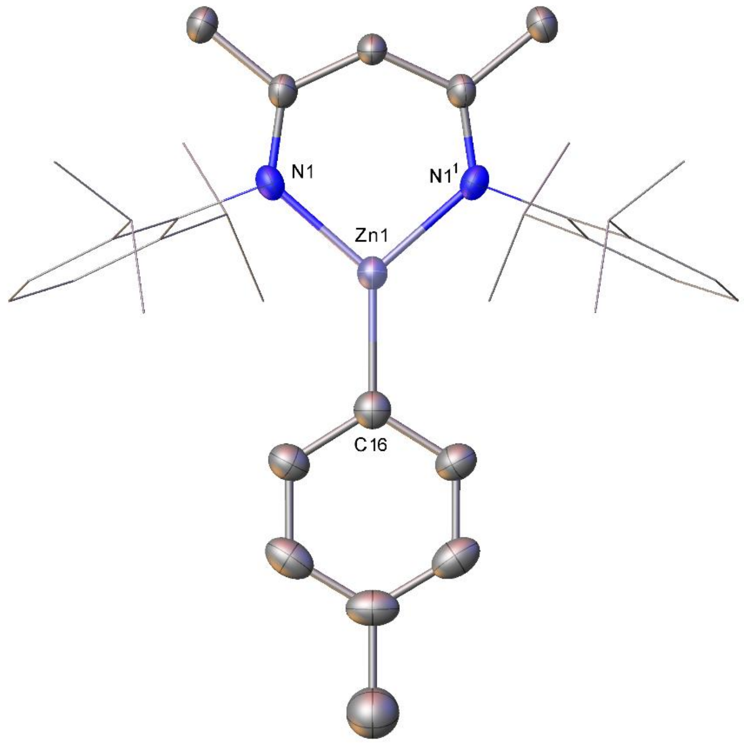

Figure S39. *Molecular structure of* ***4***^{p}*, displacement ellipsoids at 30%. For clarity, hydrogen atoms are omitted and the Dipp groups are displayed as wireframe.*

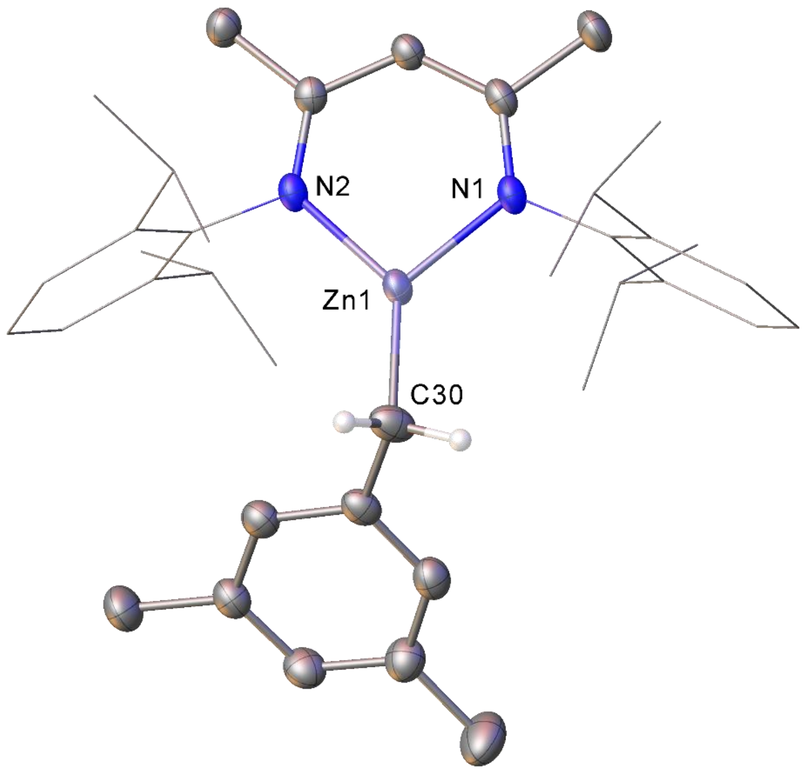

Figure S40. *Molecular structure of **5**, displacement ellipsoids at 30%. For clarity, hydrogen atoms are omitted and the Dipp groups are displayed as wireframe.*

Computational Details

DFT calculations were performed with Gaussian 16 (C.01).[10] Initial BP86[11] optimizations were carried out using the 'grid = ultrafine' option with the split-valence polarised basis set def2-SVP (BS1), for all the atoms (H, C, N and Ca).[12] Single point corrections to the BP86 results to account for dispersion employed Grimme's D3 parameter set with Becke-Johnson damping ($D3^{BJ}$),[13] and to account for solvent effects of benzene ($\varepsilon = 2.2706$) and toluene ($\varepsilon = 2.3741$) using the SMD continuum universal solvation model.[14] All energies were recomputed with a larger basis set (BS2), def2-TZVPP, a triple-zeta valence basis set with double polarisation.[2] Frequency calculations were carried out to characterise the stationary points, either as minima or transitions states. In the case of the minima, all eigenvalues of the Hessian matrix were real and positive, and for transition states they only presented one negative eigenvalue. Connections between the transition states and the corresponding minima were checked and verified via IRC (Intrinsic Reaction Coordinate) calculations. The visualisation of the stationary points was performed with CYLView software.[15]

Breakdown of Energy Contributions

Table S3. Contribution details for the relative energies of the successive corrections to the initial SCF energy . Terms used are:

$\mathbf{\Delta E_{BS1}}$	SCF energy computed with the BP86 functional and BS1
$\mathbf{\Delta G_{BS1}}$	Free energy at 298.15 K and 1 atm and BS1
$\mathbf{\Delta G_{BS1/C_6H_6}}$	Free energy corrected for Benzene solvent (SMD) with BS1
$\mathbf{\Delta G_{BS1/C_7H_8}}$	Free energy corrected for Toluene solvent (SMD) with BS1
$\mathbf{\Delta G_{BS1/D3^{BJ}/C_6H_6}}$	Free energy corrected for Benzene solvent (SMD) and dispersion ($D3^{BJ}$) with BS1
$\mathbf{\Delta G_{BS1/D3^{BJ}/C_7H_8}}$	Free energy corrected for Toluene solvent (SMD) and dispersion ($D3^{BJ}$) with BS1
$\mathbf{\Delta E_{BS2}}$	SCF energy computed with the BP86 functional and BS2
$\mathbf{\Delta G_{bnz}}$	Overall Free energy computed at the BP86-$D3^{BJ}$; SMD=Benzene/BS2//BP86/BS1 level
$\mathbf{\Delta G_{tol}}$	Overall Free energy computed at the BP86-$D3^{BJ}$; SMD=Toluene/BS2//BP86/BS1 level

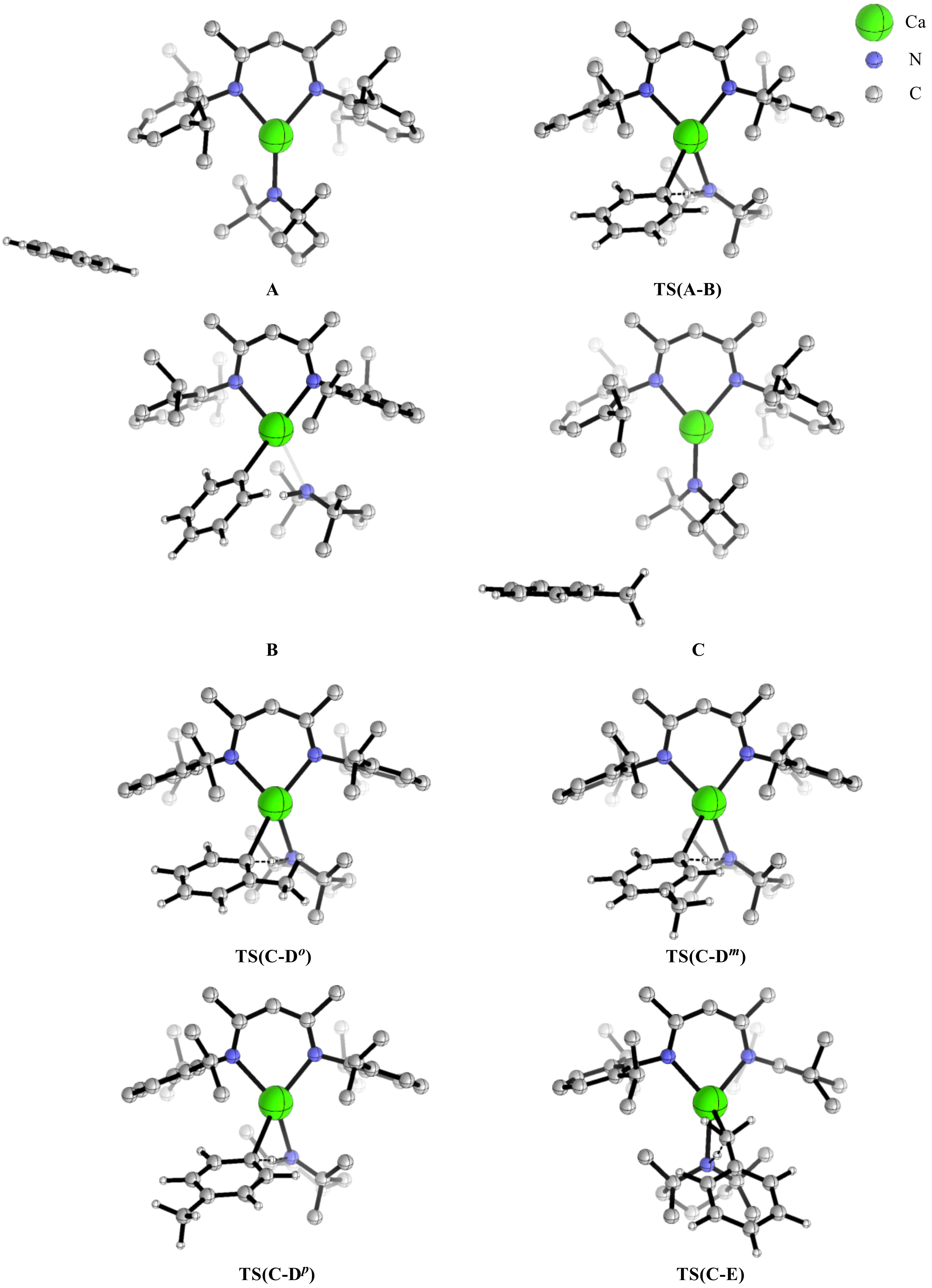

Figure S41. BP86/BS1 Optimised geometries. For clarity, hydrogen atoms from BDI ligand are omitted.

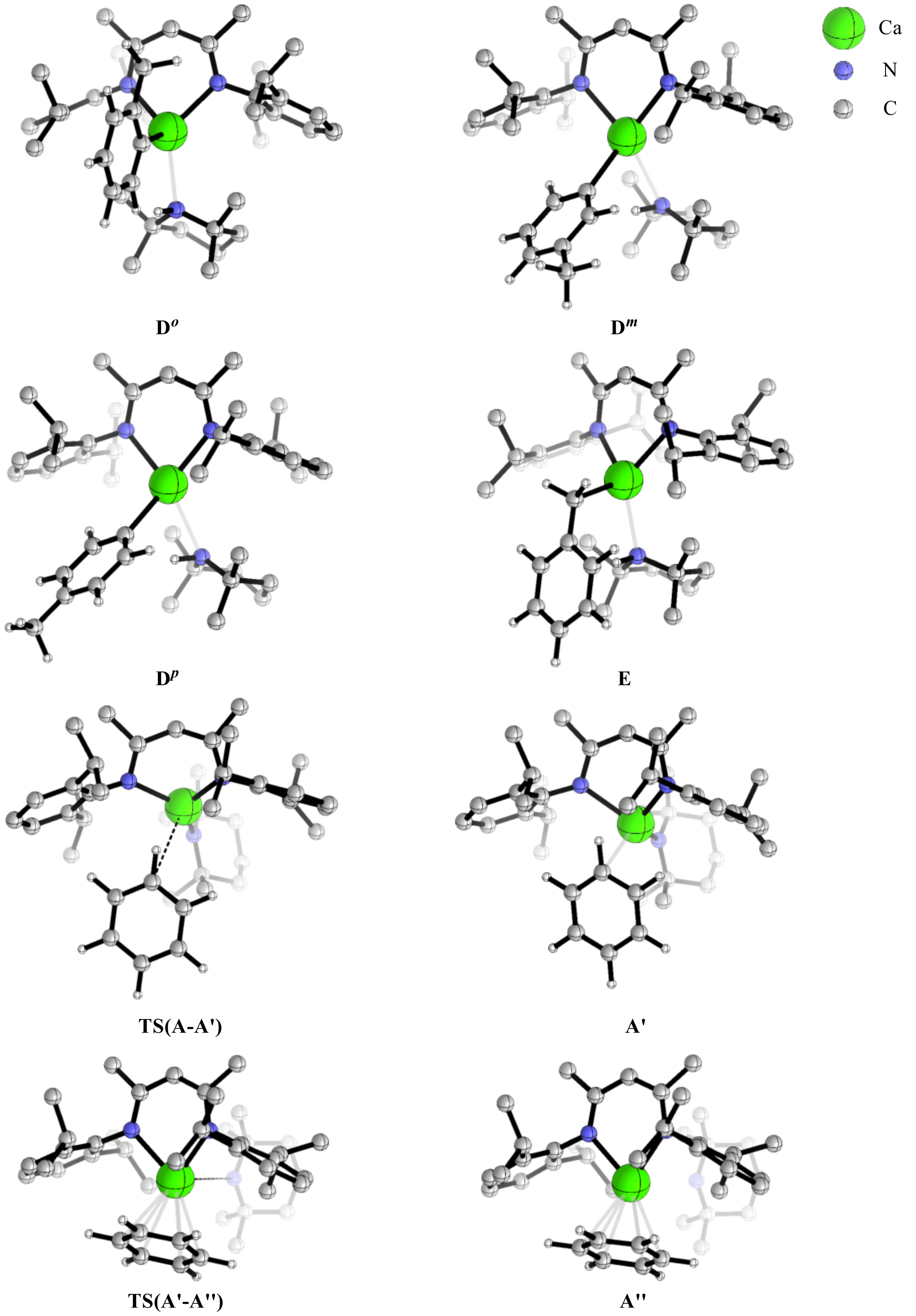

Figure S42. BP86/BS1 Optimised geometries. For clarity, hydrogen atoms from BDI ligand are omitted

Table S4. Relative energies (kcal mol^{-1}) for the formation of **B** or **D** through the C(sp^2)-H deprotonation of benzene and toluene by **A** and **C**, respectively. Free energies are quoted relative to **A** or **C**.

	ΔE_{BS1}	ΔG_{BS1}	$\Delta G_{BS1/C_6H_6}$	$\Delta G_{BS1/C_7H_8}$	$\Delta G_{BS1/D3^{BJ}/C_6H_6}$	$\Delta G_{BS1/D3^{BJ}/C_7H_8}$	ΔE_{BS2}	ΔG_{bnz}	ΔG_{tol}
**7** (+ C_6H_6)	6.4	6.6	4.1	4.7	0.0	7.7	7.8	**6.5**	**−2.3**
**A**	0.0	0.0	0.0	0.0	0.0	0.0	0.0	**0.0**	**0.0**
**TS(A-B)**	21.2	31.2	35.5	35.5	16.8	16.8	24.2	**19.9**	**19.8**
**B**	12.4	23.9	27.1	27.1	11.5	11.5	14.0	**13.0**	**13.0**
**7** (+ C_7H_8)	6.4	6.6	5.8	4.1	0.0	7.9	7.9	**6.6**	**−0.5**
**C**	0.0	0.0	0.0	0.0	0.0	0.0	0.0	**0.0**	**0.0**
**TS(C-D*ᵒ*)**	26.8	39.6	44.5	44.4	22.4	22.3	30.0	**25.6**	**25.5**
**TS(C-D*ᵐ*)**	21.3	33.3	37.6	37.6	17.8	17.8	24.4	**20.9**	**20.9**
**TS(C-D*ᵖ*)**	21.1	32.4	37.0	37.0	17.5	17.5	24.2	**20.6**	**20.6**
**TS(C-E)**	18.0	28.1	32.0	32.0	14.9	14.9	21.1	**18.0**	**18.0**
**D^{o}**	9.5	22.3	26.5	26.5	5.8	5.8	11.9	**8.2**	**8.2**
**D^{m}**	12.6	26.3	29.6	29.6	13.0	12.9	14.2	**14.6**	**14.5**
**D^{p}**	12.5	25.1	28.5	28.4	12.2	12.1	14.2	**13.8**	**13.8**
**E**	3.1	17.0	−29.1	7.7	−47.5	−10.6	5.5	**−45.1**	**−8.2**
**TS(A-A′)**	4.0	12.1	15.4	15.4	7.0	7.0	5.0	**8.0**	**8.0**
**A′**	2.8	11.6	16.9	16.9	0.3	0.3	4.5	**2.1**	**2.1**
**TS(A′-A″)**	46.2	54.0	53.2	53.0	44.3	44.0	46.4	**44.5**	**44.3**
**A″**	45.9	51.8	51.6	51.4	44.2	44.0	46.3	**44.6**	**44.4**

Table S5. Relative Gibbs energies (**ΔG_{tol}**) of backward IRC structures for the toluene deprotonation steps **TS(C-D)** and **TS(C-E)** with respect to "**C^{o}**", the lowest C isomer. (BP86-D3^{BJ}(SMD=Toluene)/def2-TZVPP//BP86/def2-SVP level, quoted in kcal mol^{-1}).

C

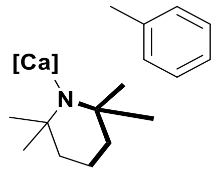

**C^{o}**	0.0
**C^{m}**	+1.9
**C^{p}**	+2.2
**C^{E}**	+1.3

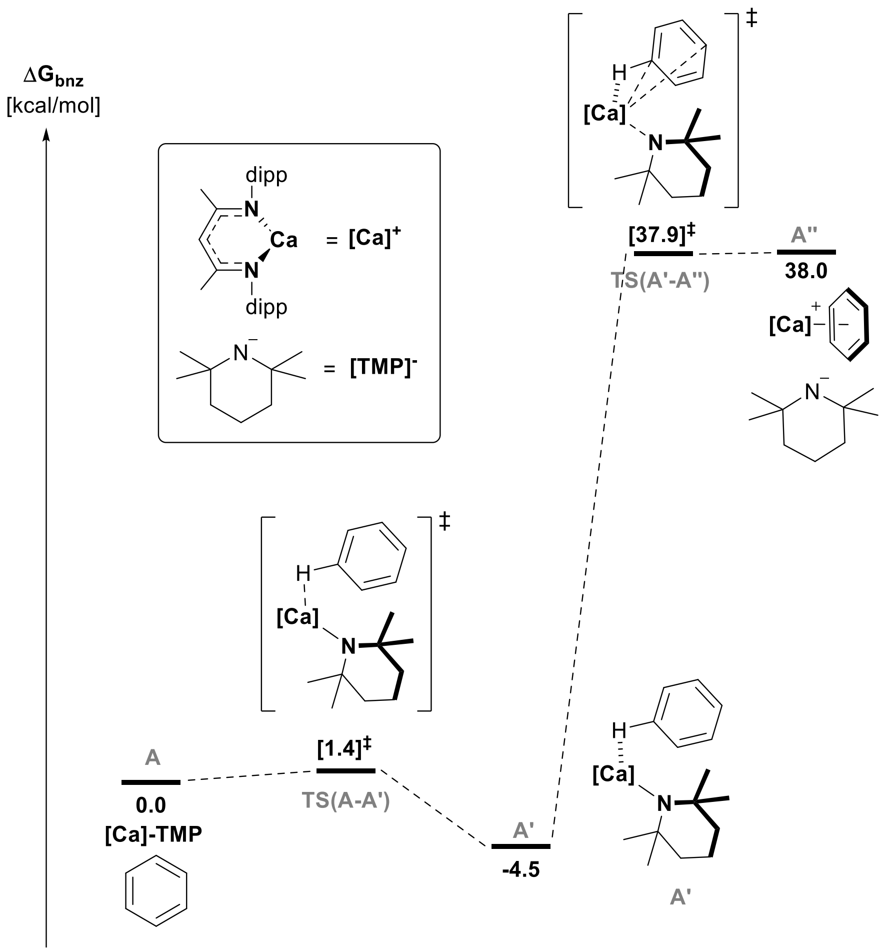

Figure S43. Computed free energy profile (BP86-D3^{BJ}(SMD=Benzene)/def2-TZVPP//BP86/def2-SVP level, quoted in kcal mol^{-1}) for the dissociation of [TMP]$^{-}$ from **A′**.

References

1. A. S. S. Wilson, M. S. Hill, M. F. Mahon, C. Dinoi, L. Maron, *Science*, 2017, **358**, 1168-1171.
2. W. S. Rees Jr, O. Just, H. Shumann and R. Weimann, *Polyhedron*, 1998, **17**, 1001-1004.
3. M. R. Crimmin, M. S. Hill, P. B. Hitchcock and M. F. Mahon, *New. J. Chem.*, 2010, **34**, 1572-1578.
4. J. Spielmann, D. Piesik, B. Wittkamp, G. Jansen and S. Harder, *Chem. Commun.*, 2009, 3455-3456.
5. M. Garçon, N. W. Mun, A. J. P. White and M. R. Crimmin, *Angew. Chem. Int. Ed.*, 2021, **60**, 6145-6153.
6. L.-L. Huang, M.-H. Xu and G.-Q. Lin, *J. Am. Chem. Soc.*, 2006, **128**, 5624-5625.
7. O. V. Dolomanov, L. J. Bourhis, R. J. Gildea, J. A. K. Howard and H. Puschmann, *J. Appl. Cryst.* 2009, **42**, 339-341.
8. G. M. Sheldrick, *Acta Cryst*. 2015, **A71**, 3-8.
9. G. M. Sheldrick, *Acta Cryst*. 2015, **C71**, 3-8.
10. M. J. Frisch, G. W. Trucks, H. B. Schlegel, G. E. Scuseria, M. A. Robb, J. R. Cheeseman, G. Scalmani, V. Barone, G. A. Petersson, H. Nakatsuji, X. Li, M. Caricato, A. V. Marenich, J. Bloino, B. G. Janesko, R. Gomperts, B. Mennucci, H. P. Hratchian, J. V. Ortiz, A. F. Izmaylov, J. L. Sonnenberg, Williams, F. Ding, F. Lipparini, F. Egidi, J. Goings, B. Peng, A. Petrone, T. Henderson, D. Ranasinghe, V. G. Zakrzewski, J. Gao, N. Rega, G. Zheng, W. Liang, M. Hada, M. Ehara, K. Toyota, R. Fukuda, J. Hasegawa, M. Ishida, T. Nakajima, Y. Honda, O. Kitao, H. Nakai, T. Vreven, K. Throssell, J. A. Montgomery Jr., J. E. Peralta, F. Ogliaro, M. J. Bearpark, J. J. Heyd, E. N. Brothers, K. N. Kudin, V. N. Staroverov, T. A. Keith, R. Kobayashi, J. Normand, K. Raghavachari, A. P. Rendell, J. C. Burant, S. S. Iyengar, J. Tomasi, M. Cossi, J. M. Millam, M. Klene, C. Adamo, R. Cammi, J. W. Ochterski, R. L. Martin, K. Morokuma, O. Farkas, J. B. Foresman, D. J. Fox, Wallingford, CT, 2016.
11. a) A. D. Becke, *Phys. Rev. A* 1988, **38**, 3098-3100; b) J. P. Perdew, *Phys. Rev. B* 1986, **33**, 8822-8824.
12. F. Weigend; R. Ahlrichs, *Phys. Chem. Chem. Phys*. 2005, **7**, 3297-3305.
13. S. Grimme, S. Ehrlich, L. Goerigk, *J. Comp. Chem*. 2011, **32**, 1456-1465.
14. J. Tomasi, B. Mennucci, R. Cammi, *Chem. Rev*. 2005, **105**, 2999-3094.
15. CYLview20; Legault, C. Y., Université de Sherbrooke, 2020 (http://www.cylview.org).

Cartesian Coordinates and Raw Electronic Energies (in Hartrees)

7

SCF (BP86) Energy = -2324.27751965
Enthalpy 0K = -2323.414921
Enthalpy 298K = -2323.363893
Free Energy 298K = -2323.498841
Lowest Frequency = 14.6964 cm^{-1}
Second Frequency = 20.2714 cm^{-1}
SCF (BP86-D3^{BJ}) Energy = -2324.52811416
SCF (C_6H_6) Energy = -2324.31452397
SCF ($C_6H_5CH_3$) Energy = -2324.31416633
SCF (BS2) Energy = -2326.13321097

```
Ca -0.04947  0.27964  0.02439
N   1.39832 -1.62534 -0.11491
N  -1.67006 -1.51177  0.17100
N   0.17575  2.52608  0.13871
C   1.08823 -2.93491 -0.13628
C  -0.20824 -3.46915  0.08735
H  -0.24753 -4.56747  0.11218
C  -1.46845 -2.83475  0.25663
C   2.18374 -3.95120 -0.42935
H   2.65850 -3.73284 -1.40907
H   1.78836 -4.98368 -0.45209
H   2.99920 -3.90175  0.32042
C  -2.65186 -3.75592  0.52288
H  -3.27166 -3.37724  1.36161
H  -2.32237 -4.78494  0.75974
H  -3.32317 -3.80448 -0.35983
C   2.75539 -1.21468 -0.28815
C   3.18038 -0.70952 -1.55785
C   4.48330 -0.18642 -1.68551
H   4.81383  0.20710 -2.66092
C   5.36718 -0.16010 -0.59855
H   6.38069  0.25505 -0.71585
C   4.95158 -0.67109  0.63967
H   5.65184 -0.65311  1.49089
C   3.66053 -1.20532  0.82300
C   2.24799 -0.72458 -2.77267
H   1.32831 -1.26856 -2.46543
C   1.83428  0.70358 -3.19266
H   1.38892  1.28248 -2.35029
H   1.09684  0.67829 -4.02321
H   2.71083  1.28973 -3.54249
C   2.85069 -1.49614 -3.96537
H   3.76349 -0.99849 -4.35644
H   2.12288 -1.55663 -4.80213
H   3.13060 -2.53154 -3.68134
C   3.25808 -1.74282  2.20138
H   2.27416 -2.24336  2.08786
C   4.25471 -2.79329  2.73697
H   4.41210 -3.62068  2.01423
H   3.88262 -3.23666  3.68473
H   5.24892 -2.34693  2.95122
C   3.07437 -0.60594  3.23044
H   4.01923 -0.04228  3.38330
H   2.75989 -1.01188  4.21551
H   2.30679  0.12679  2.90650
C  -2.97345 -0.95097  0.28370
C  -3.81604 -0.83025 -0.86769
C  -5.00621 -0.08137 -0.76022
H  -5.65542  0.02796 -1.64464
C  -5.38039  0.52665  0.44429
H  -6.30662  1.11968  0.50402
C  -4.58050  0.35486  1.58483
H  -4.90646  0.80526  2.53363
C  -3.38348 -0.38864  1.53870
C  -3.47251 -1.50440 -2.20074
H  -2.53185 -2.07426 -2.05060
C  -4.56779 -2.50754 -2.62771
H  -4.78353 -3.25027 -1.83211
H  -4.25753 -3.06299 -3.53804
H  -5.52271 -1.99152 -2.86380
C  -3.21672 -0.48817 -3.33406
H  -4.11731  0.12959 -3.53790
H  -2.95089 -1.01135 -4.27708
H  -2.38965  0.20702 -3.08736
C  -2.56722 -0.65391  2.81595
H  -2.37696 -1.75102  2.83689
C  -3.32847 -0.31183  4.10995
H  -3.48253  0.78224  4.22501
H  -2.74938 -0.65465  4.99216
H  -4.32260 -0.80217  4.14819
C  -1.17270  0.02883  2.83036
H  -0.41761 -0.60464  2.31601
H  -0.79648  0.14602  3.86760
H  -1.19183  1.04964  2.38461
C  -0.87951  3.32179 -0.51067
C   1.24297  3.29024  0.82466
C  -0.28632  4.31308 -1.58597
C   1.18647  4.67285 -1.31072
C   1.46229  4.70439  0.19368
C   2.55851  2.49079  0.67535
H   2.43614  1.44513  1.03688
H   2.88171  2.43442 -0.38410
H   3.38677  2.93193  1.27055
C   0.97686  3.45824  2.34823
H   0.01538  3.97448  2.53908
H   0.92805  2.46508  2.84588
H   1.78271  4.04383  2.84559
C  -1.81053  2.33019 -1.24111
H  -2.34845  1.65771 -0.53300
H  -2.59919  2.84686 -1.82594
H  -1.23195  1.71946 -1.97542
C  -1.78781  4.11413  0.48055
H  -2.20871  3.43009  1.24779
H  -1.23098  4.91425  1.00690
H  -2.63615  4.60233 -0.04873
H  -0.90406  5.23883 -1.62064
H  -0.36704  3.85769 -2.59750
H   1.44312  5.64495 -1.78492
H   1.85232  3.91627 -1.78012
H   2.49880  5.04817  0.40441
H   0.79288  5.45123  0.67645
```

C_6H_6

SCF (BP86) Energy = -232.076218496
Enthalpy 0K = -231.978537
Enthalpy 298K = -231.973085
Free Energy 298K = -232.004380
Lowest Frequency = 401.2144 cm^{-1}
Second Frequency = 401.2147 cm^{-1}
SCF (BP86-D3^{BJ}) Energy = -232.093113770

SCF (C_6H_6) Energy = -232.083921482
SCF ($C6H_5CH_3$) Energy = -232.083770483
SCF (BS2) Energy = -232.326587266

C	-0.00000	1.40669	0.00000
C	-1.21823	0.70334	-0.00000
C	1.21823	0.70334	0.00000
H	-2.17256	1.25433	-0.00000
H	2.17256	1.25433	0.00000
C	-1.21823	-0.70334	-0.00000
C	1.21823	-0.70334	0.00000
H	-2.17256	-1.25433	-0.00000
H	2.17256	-1.25433	0.00000
C	0.00000	-1.40669	-0.00000
H	0.00000	-2.50866	-0.00000
H	-0.00000	2.50866	-0.00000

C_7H_8

SCF (BP86) Energy = -271.362923604
Enthalpy 0K = -271.239009
Enthalpy 298K = -271.232562
Free Energy 298K = -271.268055
Lowest Frequency = -14.1456 cm^{-1}
Second Frequency = 203.4331 cm^{-1}
SCF (BP86-$D3^{BJ}$) Energy = -271.384692415
SCF (C_6H_6) Energy = -271.371237257
SCF ($C_6H_5CH_3$) Energy = -271.371051411
SCF (BS2) Energy = -271.656058462

H	-0.01214	0.74347	2.16917
C	0.02912	2.43702	-0.00000
H	-0.46854	2.85794	0.89743
H	-0.46854	2.85794	-0.89743
H	1.07584	2.81423	-0.00000
C	-0.00479	0.92388	-0.00000
C	-0.00768	0.19656	-1.21125
C	-0.00768	-1.20882	-1.21401
C	-0.00686	-1.91797	0.00000
C	-0.00768	-1.20882	1.21401
C	-0.00768	0.19656	1.21125
H	-0.01214	0.74347	-2.16917
H	-0.01231	-1.75397	-2.17177
H	-0.01033	-3.01968	0.00000
H	-0.01231	-1.75397	2.17177

A

SCF (BP86) Energy = -2556.36395942
Enthalpy 0K = -2555.403991
Enthalpy 298K = -2555.345482
Free Energy 298K = -2555.509799
Lowest Frequency = 2.8644 cm^{-1}
Second Frequency = 3.4940 cm^{-1}
SCF (BP86-$D3^{BJ}$) Energy = -2556.62876319
SCF (C_6H_6) Energy = -2556.41078571
SCF ($C_6H_5CH_3$) Energy = -2556.41031177
SCF (BS2) Energy = -2558.47018162

Ca	-0.94361	0.09511	-0.15355
N	-3.05082	-0.72987	0.63394
N	-0.29833	-2.15205	0.34387
C	-3.35172	-1.96610	1.06288
C	-2.41237	-3.01916	1.22527
H	-2.84294	-3.93941	1.65044
C	-1.02712	-3.11743	0.92570
C	-4.78828	-2.33370	1.42584
H	-5.51481	-1.56923	1.09156
H	-4.89749	-2.44841	2.52512
H	-5.06142	-3.30924	0.97509
C	-0.38605	-4.44895	1.30801
H	-0.73843	-5.26108	0.63741
H	-0.68138	-4.73535	2.33775
H	0.71815	-4.41579	1.24846
C	-4.01567	0.31393	0.57594
C	-4.46190	0.98682	1.76079
C	-5.30761	2.10697	1.62518
H	-5.65254	2.63073	2.53193
C	-5.71636	2.57431	0.36901
H	-6.37340	3.45479	0.29009
C	-5.28303	1.91018	-0.78756
H	-5.61363	2.27389	-1.77271
C	-4.43988	0.78382	-0.71205
C	-4.04152	0.54106	3.16559
H	-3.43786	-0.38329	3.05814
C	-5.25917	0.20565	4.05484
H	-5.86882	1.10940	4.26821
H	-4.92860	-0.20583	5.03209
H	-5.92697	-0.53968	3.57751
C	-3.14535	1.58925	3.86070
H	-2.23668	1.81251	3.26628
H	-2.81997	1.22751	4.85928
H	-3.68498	2.54846	4.01169
C	-4.04703	0.02833	-1.98528
H	-3.07842	-0.48663	-1.77785
C	-5.06144	-1.09775	-2.28914
H	-5.15789	-1.80119	-1.43845
H	-4.75343	-1.68077	-3.18304
H	-6.06665	-0.66938	-2.48915
C	-3.84541	0.93115	-3.21504
H	-4.80110	1.37815	-3.56133
H	-3.44137	0.34188	-4.06452
H	-3.13791	1.75951	-3.00396
C	1.05406	-2.35301	-0.05090
C	2.09047	-1.66570	0.66416
C	3.41681	-1.74723	0.19514
H	4.21776	-1.22464	0.74065
C	3.73945	-2.48738	-0.95127
H	4.78186	-2.53727	-1.30334
C	2.72544	-3.16400	-1.64260
H	2.98415	-3.74703	-2.54189
C	1.38108	-3.11646	-1.21999
C	1.78057	-0.91697	1.96376
H	0.70132	-0.63353	1.93071
C	2.60011	0.37195	2.15160
H	3.67884	0.15903	2.30494
H	2.25050	0.92453	3.04881
H	2.50245	1.04366	1.27401
C	1.93068	-1.85347	3.18432
H	1.28667	-2.75089	3.09287
H	1.65715	-1.32895	4.12459
H	2.98154	-2.20064	3.27981
C	0.32093	-3.87197	-2.02918
H	-0.64616	-3.76806	-1.49590
C	0.63937	-5.37982	-2.13279
H	1.56032	-5.56233	-2.72659
H	-0.18921	-5.92135	-2.63665
H	0.79213	-5.83894	-1.13510

```
C   0.12811 -3.26775 -3.43735
H  -0.16189 -2.19888 -3.39017
H  -0.66541 -3.81189 -3.99258
H   1.06070 -3.33221 -4.03758
C   9.44889  0.76295  1.91237
C   8.47510  1.72215  2.24487
H   8.44269  2.14460  3.26213
C   7.54335  2.14295  1.27853
H   6.78116  2.89516  1.53812
C   7.58531  1.60458 -0.02059
H   6.85566  1.93468 -0.77726
C   8.55939  0.64550 -0.35292
H   8.59310  0.22483 -1.37087
C   9.49105  0.22465  0.61342
H  10.25437 -0.52616  0.35274
N   0.01830  1.96748 -0.94238
C  -0.22426  3.28227 -0.32677
C   0.72561  1.92569 -2.23288
C  -0.83777  4.28581 -1.34852
C   0.12411  2.94375 -3.24864
C  -0.02673  4.34851 -2.65000
H  -0.93113  5.29767 -0.89328
H   0.73777  2.97395 -4.17737
H   0.97533  4.79357 -2.45723
H  10.17907  0.43374  2.66919
C  -1.25057  3.05108  0.80380
H  -0.83193  2.35046  1.56707
H  -1.50297  3.98437  1.34880
H  -2.20541  2.63645  0.40922
C   2.26818  2.13383 -2.13471
H   2.69155  1.45666 -1.36427
H   2.76935  1.91446 -3.10330
H   2.53795  3.17075 -1.85653
C   1.02785  3.92815  0.34314
H   1.51668  3.19585  1.01836
H   1.78563  4.25715 -0.39387
H   0.74790  4.82181  0.94346
C   0.51882  0.50193 -2.80045
H  -0.56819  0.28540 -2.93417
H   0.99275  0.36777 -3.79505
H   0.97710 -0.26642 -2.13415
H  -0.88556  2.57701 -3.54464
H  -0.52093  5.02627 -3.38029
H  -1.87099  3.94436 -1.58741
```

TS(A-B)

SCF (BP86) Energy = -2556.33017521
Enthalpy 0K = -2555.371625
Enthalpy 298K = -2555.315342
Free Energy 298K = -2555.460032
Lowest Frequency = -1151.4228 cm^{-1}
Second Frequency = 12.3675 cm^{-1}
SCF (BP86-D3^{BJ}) Energy = -2556.62471830
SCF (C_6H_6) Energy = -2556.37018275
SCF ($C_6H_5CH_3$) Energy = -2556.36977614
SCF (BS2) Energy = -2558.43157695

```
Ca -0.08053  0.15999 -0.03250
N  -1.40055 -1.79393 -0.45791
N   1.70546 -1.29401 -0.91203
C  -0.99273 -2.97606 -0.96289
C   0.31955 -3.27291 -1.40274
H   0.42839 -4.29131 -1.80430
C   1.54906 -2.56153 -1.33110
C  -1.96995 -4.14690 -1.03551
H  -2.99955 -3.82608 -1.28394
H  -2.02707 -4.65504 -0.04860
H  -1.63736 -4.89962 -1.77557
C   2.75506 -3.40886 -1.73636
H   2.52975 -3.98313 -2.65758
H   2.97470 -4.15518 -0.94368
H   3.67098 -2.81209 -1.89715
C  -2.75876 -1.67246 -0.02967
C  -3.14372 -2.10923  1.27710
C  -4.47531 -1.90823  1.69540
H  -4.78066 -2.23594  2.70232
C  -5.41992 -1.30644  0.85437
H  -6.45643 -1.16118  1.19792
C  -5.03886 -0.88806 -0.42941
H  -5.79030 -0.41788 -1.08119
C  -3.71900 -1.05387 -0.89735
C  -2.14416 -2.77480  2.22989
H  -1.25185 -3.05337  1.63192
C  -2.69751 -4.06817  2.86394
H  -3.53629 -3.86270  3.56242
H  -1.90635 -4.58377  3.44798
H  -3.07121 -4.77583  2.09521
C  -1.66410 -1.80002  3.32685
H  -1.16859 -0.90345  2.89902
H  -0.93221 -2.29069  4.00265
H  -2.51256 -1.43890  3.94614
C  -3.32799 -0.58939 -2.30726
H  -2.30217 -0.15977 -2.22043
C  -3.23948 -1.75101 -3.32400
H  -2.45590 -2.48537 -3.06062
H  -2.99916 -1.35875 -4.33511
H  -4.21005 -2.28731 -3.39303
C  -4.24627  0.50751 -2.87602
H  -5.25371  0.11188 -3.12639
H  -3.81864  0.91290 -3.81641
H  -4.37895  1.35188 -2.17047
C   3.03688 -0.78850 -0.74349
C   3.78060 -1.10989  0.43808
C   5.04895 -0.52330  0.62362
H   5.62014 -0.75993  1.53609
C   5.60051  0.34727 -0.32412
H   6.59340  0.79496 -0.15847
C   4.88370  0.63225 -1.49481
H   5.33314  1.29635 -2.24894
C   3.60962  0.07605 -1.73424
C   3.26414 -2.09064  1.49728
H   2.29133 -2.48806  1.14017
C   3.01234 -1.40527  2.85613
H   3.95435 -1.00559  3.28744
H   2.59349 -2.13024  3.58653
H   2.30775 -0.55466  2.76896
C   4.21928 -3.29242  1.67701
H   4.43837 -3.79990  0.71577
H   3.77727 -4.04159  2.36779
H   5.19058 -2.97607  2.11362
C   2.90544  0.34074 -3.07019
H   1.81231  0.27923 -2.87771
C   3.22925 -0.75087 -4.11696
H   4.32131 -0.79044 -4.31779
H   2.71466 -0.53481 -5.07763
H   2.90909 -1.75597 -3.78468
C   3.21722  1.72660 -3.66874
```

H	3.09491	2.54408	-2.92988
H	2.54638	1.93474	-4.52839
H	4.25665	1.78078	-4.05659
C	3.06809	2.34529	3.74697
C	1.77839	1.96744	4.16192
H	1.54315	1.89840	5.23742
C	0.78897	1.68281	3.20193
H	-0.21566	1.40608	3.57268
C	1.03073	1.75731	1.80226
H	-0.04535	2.13506	0.94816
C	2.34858	2.13982	1.42913
H	2.62252	2.22265	0.35989
C	3.35336	2.42710	2.37263
H	4.36175	2.71538	2.03304
N	-1.09095	2.38188	0.09627
C	-2.23880	2.84332	0.93436
C	-0.67189	3.25225	-1.03697
C	-3.43188	3.27684	0.04029
C	-1.89538	3.67044	-1.89741
C	-3.03151	4.26560	-1.05971
H	-4.23488	3.70090	0.68287
H	-1.56606	4.38117	-2.68806
H	-2.71844	5.23669	-0.61601
H	3.84702	2.57605	4.49202
C	-2.70023	1.64137	1.78289
H	-1.89995	1.30193	2.47285
H	-3.57343	1.90906	2.41350
H	-3.00471	0.79040	1.13911
C	0.13796	4.51533	-0.62082
H	0.95648	4.24795	0.07740
H	0.58842	5.00359	-1.51174
H	-0.49242	5.27347	-0.11937
C	-1.88134	3.99887	1.91550
H	-0.96485	3.76152	2.49162
H	-1.71257	4.96383	1.40010
H	-2.70884	4.15862	2.63947
C	0.25223	2.39592	-1.92605
H	-0.28566	1.49835	-2.31400
H	0.60318	2.95270	-2.81764
H	1.17493	2.08295	-1.38493
H	-2.27824	2.76497	-2.41961
H	-3.90464	4.49732	-1.70769
H	-3.85555	2.36348	-0.43315

B

SCF (BP86) Energy = -2556.34416584
Enthalpy 0K = -2555.380675
Enthalpy 298K = -2555.323746
Free Energy 298K = -2555.471742
Lowest Frequency = 11.4345 cm^{-1}
Second Frequency = 24.6912 cm^{-1}
SCF (BP86-$D3^{BJ}$) Energy = -2556.63381681
SCF (C_6H_6) Energy = -2556.38585195
SCF ($C_6H_5CH_3$) Energy = -2556.38546373
SCF (BS2) Energy = -2558.44794634

Ca	0.04292	0.05584	0.03204
N	-1.17169	-2.00341	-0.26869
N	1.88801	-1.34299	-0.73132
C	-0.67416	-3.20798	-0.61605
C	0.66937	-3.46722	-0.97829
H	0.85908	-4.51653	-1.24880
C	1.83880	-2.65695	-1.00207
C	-1.58040	-4.43675	-0.59074
H	-2.60794	-4.21104	-0.93548
H	-1.67442	-4.82068	0.44813
H	-1.16417	-5.25451	-1.20941
C	3.10828	-3.43596	-1.34268
H	2.92127	-4.12761	-2.18873
H	3.40920	-4.06535	-0.47873
H	3.96168	-2.78007	-1.59441
C	-2.55598	-1.90524	0.06309
C	-3.00976	-2.21880	1.38365
C	-4.37386	-2.03991	1.69291
H	-4.73256	-2.27474	2.70798
C	-5.28489	-1.57628	0.73470
H	-6.34751	-1.44809	0.99540
C	-4.83642	-1.27634	-0.56039
H	-5.56165	-0.91682	-1.30600
C	-3.48194	-1.42724	-0.92305
C	-2.04550	-2.72541	2.46166
H	-1.12175	-3.06127	1.94711
C	-2.60313	-3.93183	3.24452
H	-3.47149	-3.65247	3.87832
H	-1.82695	-4.34510	3.92228
H	-2.93387	-4.74566	2.56626
C	-1.63053	-1.59528	3.42786
H	-1.12052	-0.76376	2.89777
H	-0.92576	-1.97111	4.19930
H	-2.51229	-1.16618	3.94969
C	-3.01627	-1.10517	-2.34946
H	-2.00166	-0.65114	-2.25397
C	-2.85025	-2.36875	-3.22499
H	-2.07679	-3.05412	-2.83144
H	-2.55041	-2.08706	-4.25688
H	-3.80833	-2.92720	-3.29210
C	-3.91735	-0.09564	-3.08359
H	-4.89951	-0.53956	-3.35223
H	-3.44032	0.22393	-4.03341
H	-4.11312	0.81094	-2.47637
C	3.15795	-0.68187	-0.67793
C	3.98865	-0.78723	0.48478
C	5.17865	-0.03243	0.53621
H	5.81573	-0.10043	1.43296
C	5.56990	0.79869	-0.51992
H	6.50294	1.38072	-0.45437
C	4.76806	0.87696	-1.66739
H	5.09167	1.51577	-2.50310
C	3.56546	0.14822	-1.77487
C	3.65766	-1.70832	1.66515
H	2.70634	-2.22873	1.42787
C	3.44326	-0.93117	2.98135
H	4.36879	-0.40168	3.29289
H	3.17044	-1.63005	3.80096
H	2.64032	-0.17455	2.88518
C	4.74824	-2.78695	1.86208
H	4.95281	-3.35486	0.93214
H	4.44242	-3.51115	2.64689
H	5.70775	-2.33306	2.19022
C	2.75193	0.20012	-3.07362
H	1.67930	0.11057	-2.78769
C	3.05804	-1.00265	-3.99581
H	4.13306	-1.01598	-4.27575
H	2.46330	-0.93906	-4.93216
H	2.81988	-1.96868	-3.51332
C	2.93167	1.50807	-3.86767
H	2.81649	2.40837	-3.23124

H 2.18676 1.56587 -4.68872
H 3.93413 1.56233 -4.34293
C 2.39170 3.36411 3.56150
C 1.26601 2.66620 4.03542
H 0.92112 2.81884 5.07308
C 0.58292 1.77250 3.18481
H -0.29366 1.24981 3.61684
C 0.96931 1.51227 1.83805
H -0.58303 2.47378 0.62219
C 2.12034 2.24279 1.41573
H 2.52983 2.09444 0.39425
C 2.82072 3.14857 2.24035
H 3.70840 3.67865 1.85396
N -1.36488 2.44076 -0.07203
C -2.61360 2.79652 0.69334
C -0.96510 3.27272 -1.25484
C -3.77840 3.01613 -0.29902
C -2.18840 3.47331 -2.18099
C -3.43033 3.98032 -1.43923
H -4.66548 3.37043 0.26840
H -1.90219 4.16167 -3.00534
H -3.25847 5.00661 -1.04633
H 2.93189 4.06628 4.21812
C -2.93858 1.59260 1.59657
H -2.12035 1.41599 2.32442
H -3.86586 1.77681 2.17735
H -3.09575 0.67294 0.99687
C -0.32529 4.63329 -0.87407
H 0.48308 4.49051 -0.12687
H 0.11948 5.11469 -1.77045
H -1.05785 5.34485 -0.44894
C -2.42684 4.03602 1.60735
H -1.52488 3.91955 2.24373
H -2.32698 4.98304 1.04319
H -3.30272 4.14616 2.28056
C 0.09742 2.45478 -2.01316
H -0.32090 1.47798 -2.34765
H 0.43987 2.98000 -2.92705
H 1.00457 2.29150 -1.38841
H -2.43368 2.49655 -2.65390
H -4.28494 4.06498 -2.14432
H -4.05648 2.02962 -0.73168

C = C°

SCF (BP86) Energy = -2595.65059655
Enthalpy 0K = -2594.664380
Enthalpy 298K = -2594.603943
Free Energy 298K = -2594.776205
Lowest Frequency = 1.7915 cm^{-1}
Second Frequency = 2.3860 cm^{-1}
SCF (BP86-D3^{BJ}) Energy = -2595.91940653
SCF (C_6H_6) Energy = -2595.69831778
SCF ($C_6H_5CH_3$) Energy = -2595.69782207
SCF (BS2) Energy = -2597.79973185

Ca -1.17670 0.03100 0.25215
N -3.08402 -0.59559 -1.05492
N -1.44117 2.04534 -1.00933
C -3.74577 0.13508 -1.96620
C -3.35295 1.42614 -2.40980
H -3.98949 1.83597 -3.20970
C -2.30513 2.29623 -2.00572
C -5.01677 -0.40293 -2.61846
H -5.40776 -1.29891 -2.10037
H -4.82682 -0.67906 -3.67735
H -5.80424 0.37754 -2.62902
C -2.22037 3.59983 -2.79559
H -1.27925 4.14818 -2.60167
H -3.06489 4.27232 -2.53473
H -2.29938 3.39599 -3.88265
C -3.46970 -1.92080 -0.71109
C -3.20194 -3.02742 -1.58243
C -3.47506 -4.33214 -1.12274
H -3.26876 -5.18810 -1.78626
C -3.99644 -4.56911 0.15615
H -4.19640 -5.59850 0.49311
C -4.26355 -3.48401 1.00330
H -4.68264 -3.67283 2.00374
C -4.01292 -2.15868 0.59583
C -2.61675 -2.84274 -2.98677
H -2.54025 -1.75277 -3.17799
C -3.52775 -3.44815 -4.07732
H -3.58712 -4.55406 -3.99082
H -3.13232 -3.21856 -5.08951
H -4.56263 -3.05484 -4.01643
C -1.19014 -3.42326 -3.09881
H -0.50182 -2.96683 -2.35936
H -0.77120 -3.24367 -4.11179
H -1.18341 -4.52012 -2.92305
C -4.38948 -0.98732 1.50818
H -3.73102 -0.12899 1.23191
C -5.83291 -0.51317 1.22307
H -5.96775 -0.23126 0.15997
H -6.09331 0.36801 1.84721
H -6.55854 -1.32226 1.45286
C -4.19123 -1.26923 3.00795
H -4.90903 -2.02814 3.38463
H -4.36159 -0.34595 3.60011
H -3.16545 -1.63244 3.22510
C -0.49472 3.00935 -0.56272
C 0.89793 2.77597 -0.81663
C 1.85179 3.65075 -0.25962
H 2.92179 3.47882 -0.45378
C 1.46536 4.74117 0.53282
H 2.22594 5.41238 0.96197
C 0.10385 4.96995 0.77262
H -0.19559 5.82980 1.39431
C -0.89435 4.12714 0.24199
C 1.34371 1.63974 -1.74177
H 0.55097 0.85443 -1.70368
C 2.67336 0.98349 -1.33090
H 3.53378 1.67296 -1.46166
H 2.87668 0.09453 -1.96383
H 2.65203 0.65423 -0.27184
C 1.39192 2.11552 -3.21163
H 0.41457 2.51863 -3.54388
H 1.66786 1.28117 -3.89108
H 2.14816 2.91999 -3.33359
C -2.36423 4.43049 0.55353
H -2.98453 3.70485 -0.01126
C -2.77056 5.84928 0.09782
H -2.24242 6.63238 0.68251
H -3.85999 6.00996 0.24258
H -2.53786 6.02162 -0.97254
C -2.69104 4.23521 2.05031
H -2.46588 3.20410 2.38915
H -3.76696 4.43071 2.24574

H -2.10350 4.92969 2.68799
C 10.43365 -0.06218 -1.31420
C   9.91733 -1.35313 -1.52606
C   8.79018 -1.81431 -0.81230
C   8.19117 -0.93812 0.12177
H   7.30690 -1.27462 0.68881
C   8.70294 0.35176 0.33711
H   8.21815 1.01679 1.07009
C   9.82845 0.79582 -0.38076
H 10.23011 1.80798 -0.21365
N   0.21467 -0.77928 1.81986
C   0.76890 -2.14056 1.74452
C   0.41062 -0.01777 3.06487
C   0.38711 -2.98266 2.99864
C   0.04680 -0.86790 4.31971
C   0.71596 -2.24848 4.30569
H   0.88797 -3.97664 2.96795
H   0.30315 -0.31392 5.25103
H   1.81823 -2.14222 4.42018
C   0.13247 -2.80214 0.50268
H   0.40897 -2.23596 -0.42012
H   0.48765 -3.84157 0.34363
H -0.97671 -2.84585 0.58767
C   1.83784 0.58660 3.23808
H   2.12816 1.13730 2.31946
H   1.87738 1.29523 4.09459
H   2.60632 -0.18872 3.42227
C   2.31305 -2.20179 1.53193
H   2.60475 -1.54846 0.68381
H   2.87969 -1.86421 2.42100
H   2.64842 -3.23790 1.30493
C -0.56160 1.18363 3.00251
H -1.61713 0.83146 2.91135
H -0.51612 1.81460 3.91444
H -0.31345 1.85852 2.14953
H -1.05797 -1.01272 4.32781
H   0.37932 -2.84759 5.18011
H -0.70980 -3.17422 2.96274
H 11.31419 0.27464 -1.88492
C   8.24244 -3.20910 -1.02302
H   8.44168 -3.85734 -0.14166
H   7.14177 -3.19723 -1.16886
H   8.69882 -3.69944 -1.90612
H 10.39849 -2.01854 -2.26241

C^m

SCF (BP86) Energy = -2595.65059882
Enthalpy 0K = -2594.664201
Enthalpy 298K = -2594.603897
Free Energy 298K = -2594.772331
Lowest Frequency = 4.4039 cm^{-1}
Second Frequency = 4.9271 cm^{-1}
SCF (BP86-$D3^{BJ}$) Energy = -2595.92109247
SCF (C_6H_6) Energy = -2595.69795049
SCF ($C_6H_5CH_3$) Energy = -2595.69742617
SCF (BS2) Energy = -2597.79931475

Ca -1.10799 0.00302 0.30618
N -2.88118 0.49009 -1.22712
N -0.21971 2.01310 -0.62082
C -2.96948 1.52123 -2.08059
C -1.93494 2.46745 -2.31701
H -2.16270 3.18144 -3.12291
C -0.67146 2.67494 -1.70229
C -4.24165 1.75525 -2.89089
H -5.07448 1.10500 -2.56333
H -4.06104 1.56347 -3.96964
H -4.55962 2.81430 -2.80515
C   0.21893 3.71547 -2.37241
H   0.79855 4.30975 -1.63967
H -0.36649 4.40075 -3.01461
H   0.96410 3.20446 -3.02073
C -3.90525 -0.48787 -1.09267
C -4.10844 -1.50130 -2.08645
C -5.03869 -2.52824 -1.82452
H -5.19826 -3.31226 -2.58301
C -5.76106 -2.58108 -0.62512
H -6.47777 -3.39738 -0.44310
C -5.56583 -1.58378 0.34116
H -6.14313 -1.62176 1.27780
C -4.65216 -0.53183 0.13212
C -3.34606 -1.51419 -3.41604
H -2.71224 -0.60437 -3.44903
C -4.30161 -1.46609 -4.62896
H -4.91560 -2.38918 -4.69928
H -3.72772 -1.38018 -5.57600
H -4.99991 -0.60687 -4.57256
C -2.40025 -2.72952 -3.53048
H -1.66851 -2.76049 -2.69884
H -1.83228 -2.69548 -4.48449
H -2.96452 -3.68625 -3.51153
C -4.52242 0.59256 1.16434
H -3.49695 1.02286 1.06037
C -5.49200 1.75073 0.83516
H -5.32683 2.14319 -0.18775
H -5.36610 2.59156 1.54996
H -6.54482 1.40215 0.89763
C -4.69460 0.13257 2.62271
H -5.73839 -0.18042 2.83608
H -4.46113 0.96398 3.32002
H -4.02751 -0.71972 2.86732
C   1.02028 2.38682 -0.01839
C   2.24667 1.78958 -0.44810
C   3.44080 2.12581 0.22229
H   4.38942 1.66755 -0.10145
C   3.44468 3.03093 1.29201
H   4.38837 3.28528 1.80024
C   2.23768 3.60428 1.71888
H   2.24769 4.30656 2.56705
C   1.01374 3.29686 1.09079
C   2.27865 0.77128 -1.59055
H   1.28283 0.80149 -2.08227
C   2.48436 -0.66025 -1.04641
H   3.48357 -0.76712 -0.57328
H   2.41617 -1.41217 -1.86164
H   1.73507 -0.92484 -0.26581
C   3.33202 1.10622 -2.66621
H   3.20042 2.13453 -3.06226
H   3.25647 0.39887 -3.51901
H   4.36619 1.02959 -2.26936
C -0.29040 3.93838 1.57935
H -1.10889 3.23468 1.30569
C -0.59203 5.27150 0.85698
H   0.22269 6.00652 1.03249
H -1.53722 5.71605 1.23537
H -0.70052 5.13861 -0.23640
C -0.33679 4.15177 3.10509

H	-0.07741	3.22981	3.66315
H	-1.35449	4.46717	3.41717
H	0.36016	4.95223	3.43301
C	8.36829	-1.16849	-1.96781
C	7.76598	-2.22029	-1.24141
C	7.35766	-1.96130	0.08596
H	6.87936	-2.76489	0.67063
C	7.54995	-0.69778	0.67150
H	7.22225	-0.52082	1.70860
C	8.15490	0.33717	-0.06333
H	8.30678	1.32810	0.39347
C	8.56258	0.09653	-1.38753
H	9.03394	0.90106	-1.97495
N	-0.43937	-1.56699	1.76644
C	-0.52390	-3.00677	1.47200
C	-0.11873	-1.15969	3.14426
C	-1.42040	-3.74747	2.50872
C	-1.00257	-1.91425	4.18327
C	-1.00970	-3.43133	3.95342
H	-1.40179	-4.84570	2.32568
H	-0.67465	-1.67331	5.21988
H	-0.00371	-3.85671	4.16870
C	-1.18587	-3.12751	0.08185
H	-0.55319	-2.63030	-0.69272
H	-1.30218	-4.18218	-0.24400
H	-2.20173	-2.67155	0.07504
C	1.38523	-1.30426	3.53053
H	2.02225	-0.83613	2.75202
H	1.60347	-0.81072	4.50325
H	1.69712	-2.36202	3.62635
C	0.85384	-3.73159	1.36905
H	1.53344	-3.16597	0.69898
H	1.35986	-3.82727	2.34902
H	0.73812	-4.75976	0.96060
C	-0.44641	0.34869	3.24285
H	-1.52204	0.53792	3.01179
H	-0.26786	0.75259	4.26110
H	0.19870	0.94484	2.55470
H	-2.04683	-1.53840	4.08249
H	-1.70376	-3.92479	4.66871
H	-2.47197	-3.41338	2.35469
C	7.59053	-3.59089	-1.85799
H	6.73949	-4.13847	-1.40465
H	7.41721	-3.52915	-2.95186
H	8.49846	-4.21643	-1.70784
H	8.68928	-1.34537	-3.00818

C^{P}

SCF (BP86) Energy = -2595.65055855
Enthalpy 0K = -2594.664201
Enthalpy 298K = -2594.603869
Free Energy 298K = -2594.772239
Lowest Frequency = 4.0458 cm^{-1}
Second Frequency = 5.4337 cm^{-1}
SCF (BP86-D3^{BJ}) Energy = -2595.92078279
SCF (C_6H_6) Energy = -2595.69777455
SCF ($C_6H_5CH_3$) Energy = -2595.69724881
SCF (BS2) Energy = -2597.79932083

Ca	-1.17048	0.08322	-0.26871
N	-2.93811	-0.79400	1.08866
N	-0.19561	-2.03158	0.24209
C	-2.98101	-1.97880	1.71584
C	-1.89893	-2.89902	1.78223
H	-2.09690	-3.77073	2.42419
C	-0.62299	-2.91971	1.15880
C	-4.24993	-2.43803	2.42899
H	-5.11210	-1.77706	2.22051
H	-4.09538	-2.46384	3.52831
H	-4.50873	-3.47130	2.12002
C	0.31020	-4.03593	1.61468
H	-0.24658	-4.86352	2.09404
H	1.02796	-3.63712	2.36465
H	0.92024	-4.44083	0.78394
C	-4.01309	0.13659	1.12164
C	-4.29726	0.91516	2.29187
C	-5.27737	1.92577	2.20976
H	-5.49888	2.53011	3.10482
C	-5.97234	2.18545	1.02121
H	-6.72894	2.98495	0.98267
C	-5.69838	1.41626	-0.11882
H	-6.25446	1.61451	-1.04804
C	-4.73245	0.39093	-0.09417
C	-3.57052	0.69690	3.62373
H	-2.88508	-0.16582	3.49617
C	-4.55221	0.35309	4.76603
H	-5.22094	1.20924	4.99804
H	-3.99849	0.10693	5.69684
H	-5.19712	-0.51218	4.51229
C	-2.70110	1.91122	4.01661
H	-1.95046	2.14871	3.23693
H	-2.15779	1.71323	4.96502
H	-3.31986	2.82103	4.16990
C	-4.51670	-0.49348	-1.32626
H	-3.47120	-0.88377	-1.27756
C	-5.42582	-1.74243	-1.26700
H	-5.25999	-2.32413	-0.33865
H	-5.23772	-2.41419	-2.13137
H	-6.49510	-1.44270	-1.29171
C	-4.68330	0.24205	-2.66763
H	-5.73843	0.53746	-2.84765
H	-4.38781	-0.41811	-3.50954
H	-4.06107	1.15962	-2.71449
C	1.05814	-2.22453	-0.41439
C	2.26073	-1.68146	0.13702
C	3.46548	-1.82369	-0.58193
H	4.39592	-1.40511	-0.16517
C	3.50319	-2.48671	-1.81583
H	4.45513	-2.59211	-2.36008
C	2.31922	-3.00735	-2.35887
H	2.35499	-3.51829	-3.33376
C	1.08570	-2.88506	-1.68777
C	2.25629	-0.92457	1.46761
H	1.26222	-1.09219	1.93465
C	2.41089	0.59557	1.23829
H	3.40445	0.83403	0.80265
H	2.31958	1.15563	2.19364
H	1.64956	0.99234	0.52877
C	3.32140	-1.44182	2.45603
H	3.22583	-2.53415	2.62719
H	3.22285	-0.93158	3.43751
H	4.35178	-1.24920	2.08965
C	-0.19274	-3.46336	-2.30564
H	-1.03884	-2.87066	-1.89001
C	-0.43092	-4.93162	-1.88444
H	0.41254	-5.57625	-2.21286
H	-1.36000	-5.32613	-2.34852

H -0.53414 -5.03955 -0.78768
C -0.24032 -3.34743 -3.84183
H -0.02739 -2.31627 -4.18860
H -1.24470 -3.63546 -4.21716
H   0.49026 -4.02584 -4.33172
C   8.72271 0.88942 1.75496
C   7.91160 2.04245 1.66220
H   7.83127 2.71738 2.53091
C   7.20570 2.34114 0.48412
H   6.57863 3.24603 0.43651
C   7.29554 1.48754 -0.62968
H   6.74177 1.71913 -1.55331
C   8.09587 0.33393 -0.55186
H   8.17222 -0.34427 -1.41714
C   8.79942 0.03978 0.62880
H   9.42175 -0.86950 0.67981
N -0.54135 1.95511 -1.33786
C -0.70556 3.28978 -0.73916
C -0.17622 1.87430 -2.76154
C -1.61981 4.19764 -1.61490
C -1.07747 2.79817 -3.63587
C -1.16594 4.22591 -3.08076
H -1.66178 5.22844 -1.19580
H -0.71881 2.80554 -4.69010
H -0.17898 4.73344 -3.16791
C -1.39873 3.07084 0.62352
H -0.75611 2.44437 1.28862
H -1.57489 4.02113 1.16930
H -2.39034 2.57920 0.50011
C   1.32549 2.16653 -3.06358
H   1.97051 1.56456 -2.39083
H   1.58469 1.91207 -4.11498
H   1.58727 3.23154 -2.91348
C   0.63029 4.03780 -0.43921
H   1.32547 3.37164 0.11185
H   1.14948 4.36982 -1.35890
H   0.45311 4.94484 0.17983
C -0.42697 0.41112 -3.19638
H -1.49667 0.12919 -3.04692
H -0.20622 0.24792 -4.27184
H   0.23111 -0.29268 -2.63385
H -2.10314 2.36257 -3.64983
H -1.87049 4.83228 -3.69133
H -2.65529 3.78907 -1.56926
C   9.50913 0.58909 3.01236
H   9.00377 0.98552 3.91654
H   9.65912 -0.50096 3.15172
H 10.51922 1.05432 2.97367

C^E
SCF (BP86) Energy = -2595.65084693
Enthalpy 0K = -2594.664561
Enthalpy 298K = -2594.604153
Free Energy 298K = -2594.774226
Lowest Frequency = 2.6902 cm^{-1}
Second Frequency = 3.9595 cm^{-1}
SCF (BP86-$D3^{BJ}$) Energy =
-2595.92099528
SCF (C_6H_6) Energy = -2595.69743300
SCF ($C_6H_5CH_3$) Energy = -2595.69691735
SCF (BS2) Energy = -2597.79970206

Ca -1.29343 -0.22329 -0.13684
N -0.70882 1.88822 0.81241
N   0.31991 -0.97830 1.47045
C   0.30101 2.15564 1.66212
C   1.06362 1.18033 2.35426
H   1.79225 1.60051 3.06367
C   1.04644 -0.24236 2.32325
C   0.68089 3.60684 1.92365
H   1.55826 3.68699 2.59214
H -0.15846 4.17257 2.37609
H   0.91513 4.12142 0.96821
C   1.94673 -0.91002 3.35886
H   1.78688 -0.45332 4.35668
H   3.01669 -0.75533 3.10473
H   1.76843 -1.99937 3.43111
C -1.49708 2.94712 0.26896
C -1.27042 3.37669 -1.07816
C -2.15394 4.30964 -1.65905
H -1.98519 4.63701 -2.69821
C -3.23644 4.83391 -0.94039
H -3.91896 5.55858 -1.41195
C -3.43630 4.43509 0.38922
H -4.28023 4.86074 0.95692
C -2.58593 3.50418 1.01918
C -0.07976 2.86078 -1.89135
H   0.49808 2.18483 -1.22307
C   0.87314 4.00249 -2.30602
H   0.37718 4.71455 -2.99949
H   1.76678 3.59848 -2.82738
H   1.22371 4.58261 -1.42747
C -0.52988 2.04250 -3.12046
H -1.20379 1.20127 -2.83881
H   0.34525 1.61990 -3.65907
H -1.09046 2.67387 -3.84255
C -2.84813 3.12555 2.48194
H -2.02492 2.45490 2.80432
C -2.83704 4.36075 3.40991
H -1.90254 4.95037 3.30759
H -2.92831 4.05204 4.47286
H -3.68428 5.04487 3.19078
C -4.16869 2.34501 2.65697
H -5.04370 2.95375 2.34374
H -4.32059 2.06410 3.72093
H -4.18048 1.41495 2.05489
C   0.40314 -2.39837 1.42199
C   1.52472 -3.06221 0.82498
C   1.47814 -4.46371 0.67534
H   2.33550 -4.98125 0.21442
C   0.37056 -5.21390 1.09207
H   0.35754 -6.30717 0.95874
C -0.72104 -4.56135 1.68288
H -1.58532 -5.15473 2.01900
C -0.72881 -3.16358 1.86000
C   2.76393 -2.30587 0.33370
H   2.63342 -1.23654 0.59789
C   2.91552 -2.37632 -1.20161
H   3.05526 -3.42275 -1.54756
H   3.79919 -1.79196 -1.53440
H   2.02329 -1.97348 -1.72161
C   4.05491 -2.80414 1.02031
H   3.97356 -2.77348 2.12571
H   4.92239 -2.17912 0.72111
H   4.28900 -3.85164 0.73379
C -1.89658 -2.48432 2.58183
H -1.93838 -1.42770 2.22319
C -1.62424 -2.39733 4.10097

```
H  -1.54756 -3.41531  4.53889
H  -2.44476 -1.86052  4.62284
H  -0.67617 -1.86553  4.31608
C  -3.26636 -3.12852  2.30552
H  -3.46365 -3.21965  1.21735
H  -4.07793 -2.51724  2.75244
H  -3.34495 -4.14174  2.75297
C   7.83057 -0.37604 -2.22222
C   7.27070  0.88470 -2.48997
C   7.21933  1.86393 -1.48194
C   7.72562  1.60778 -0.18920
H   7.00938  3.49872  0.62933
C   8.28316  0.33376  0.06513
H   8.68217  0.10893  1.06867
C   8.33689 -0.64801 -0.93809
H   8.77673 -1.63373 -0.71580
N  -2.66330 -1.02852 -1.73163
C  -4.09634 -0.69075 -1.68827
C  -2.22727 -2.06798 -2.68001
C  -4.98370 -1.96949 -1.60103
C  -3.11166 -3.34567 -2.56949
C  -4.60837 -3.01757 -2.65695
H  -6.06131 -1.69958 -1.67797
H  -2.82472 -4.08661 -3.34962
H  -4.86128 -2.64498 -3.67503
C  -4.31190  0.13027 -0.39635
H  -3.73252  1.08328 -0.42307
H  -5.37284  0.42040 -0.24797
H  -4.01652 -0.46512  0.50074
C  -2.16936 -1.60992 -4.17035
H  -1.60533 -0.65871 -4.25780
H  -1.66467 -2.37152 -4.80473
H  -3.17321 -1.44031 -4.60484
C  -4.58872  0.20882 -2.86320
H  -3.92079  1.08777 -2.97380
H  -4.59968 -0.32730 -3.83159
H  -5.62216  0.57892 -2.68354
C  -0.78224 -2.44046 -2.28297
H  -0.73339 -2.83401 -1.24266
H  -0.34603 -3.21855 -2.94334
H  -0.11477 -1.54908 -2.37197
H  -2.90671 -3.82161 -1.58322
H  -5.21456 -3.93923 -2.51549
H  -4.83859 -2.41955 -0.59208
H   7.87178 -1.14498 -3.01001
H   6.86855  1.10931 -3.49109
C   7.69544  2.66803  0.89000
H   8.70433  3.10886  1.04842
H   7.37148  2.24908  1.86556
H   6.77592  2.84954 -1.70185
```

TS(C–D°)

SCF (BP86) Energy = -2595.60784887
Enthalpy 0K = -2594.622702
Enthalpy 298K = -2594.564716
Free Energy 298K = -2594.713142
Lowest Frequency = -1239.4149 cm^{-1}
Second Frequency = 9.3208 cm^{-1}
SCF (BP86-$D3^{BJ}$) Energy = -2595.91183660
SCF (C_6H_6) Energy = -2595.64778230
SCF ($C_6H_5CH_3$) Energy = -2595.64737288
SCF (BS2) Energy = -2597.75193313

```
Ca -0.07159  0.13823 -0.03094
N  -1.51260 -1.76899 -0.38506
N   1.64919 -1.42928 -0.84087
C  -1.14650 -2.97471 -0.86799
C   0.15420 -3.34003 -1.29213
H   0.21274 -4.37070 -1.67148
C   1.42023 -2.69624 -1.23099
C  -2.16685 -4.10958 -0.93530
H  -3.18974 -3.75312 -1.15969
H  -2.21832 -4.62822  0.04615
H  -1.87330 -4.86410 -1.68998
C   2.58077 -3.62029 -1.59953
H   2.84015 -4.26198 -0.73030
H   3.49734 -3.07407 -1.88668
H   2.28861 -4.30072 -2.42362
C  -2.87788 -1.60022  0.01239
C  -3.30710 -2.00022  1.31712
C  -4.64439 -1.76294  1.69752
H  -4.98028 -2.06293  2.70368
C  -5.55562 -1.16209  0.82111
H  -6.59720 -0.98805  1.13461
C  -5.13347 -0.78333 -0.46210
H  -5.85957 -0.31632 -1.14386
C  -3.80634 -0.98637 -0.89357
C  -2.35896 -2.67482  2.31450
H  -1.41198 -2.89139  1.77744
C  -2.91361 -4.01589  2.84184
H  -3.82070 -3.86835  3.46566
H  -2.15912 -4.52782  3.47579
H  -3.18860 -4.70338  2.01583
C  -2.01555 -1.74117  3.49438
H  -1.57394 -0.78469  3.14701
H  -1.28917 -2.22055  4.18428
H  -2.92148 -1.48341  4.08331
C  -3.37940 -0.56599 -2.30719
H  -2.35800 -0.12932 -2.20637
C  -3.26315 -1.75516 -3.28906
H  -2.47917 -2.47620 -2.99267
H  -3.00656 -1.38781 -4.30552
H  -4.22849 -2.30018 -3.36277
C  -4.28712  0.50863 -2.93354
H  -5.28503  0.09884 -3.19895
H  -3.83625  0.88710 -3.87420
H  -4.44469  1.37390 -2.25911
C   3.01820 -1.01709 -0.70643
C   3.74787 -1.33938  0.48230
C   5.07510 -0.88277  0.61069
H   5.63855 -1.12200  1.52726
C   5.69503 -0.13800 -0.40018
H   6.73502  0.20534 -0.28090
C   4.98188  0.16078 -1.56944
H   5.47791  0.73323 -2.36874
C   3.64931 -0.26486 -1.74961
C   3.14274 -2.17267  1.61734
H   2.13935 -2.51118  1.28428
C   2.94805 -1.33582  2.89908
H   3.92237 -0.99298  3.30731
H   2.44773 -1.93653  3.68875
H   2.34112 -0.42752  2.71089
C   3.97876 -3.43480  1.92445
H   4.15112 -4.05186  1.01900
H   3.46503 -4.06885  2.67795
H   4.97460 -3.17239  2.34093
C   2.93502  0.02675 -3.07409
```

```
H   1.84408  0.00727 -2.86181
C   3.19911 -1.07369 -4.12797
H   4.28515 -1.15439 -4.34854
H   2.67611 -0.83775 -5.07941
H   2.84769 -2.06714 -3.79018
C   3.28865  1.40468 -3.66850
H   3.18736  2.22264 -2.92628
H   2.62585  1.63619 -4.52846
H   4.33038  1.43166 -4.05269
C   3.72120  2.66154  2.76305
C   2.58818  2.43314  3.56176
C   1.34931  2.03947  3.00493
C   1.20664  1.88993  1.58401
H   0.02366  2.20273  0.79531
C   2.38229  2.12572  0.82240
H   2.35001  2.02948 -0.27977
C   3.62383  2.49083  1.37522
H   4.49998  2.64050  0.72461
N  -1.02535  2.37760 -0.07363
C  -2.14250  2.89557  0.77619
C  -0.66946  3.15969 -1.29555
C  -3.37071  3.30732 -0.07902
C  -1.93668  3.56332 -2.10028
C  -3.00969  4.23551 -1.24108
H  -4.13233  3.77230  0.58521
H  -1.63338  4.21626 -2.94862
H  -2.65033  5.21842 -0.86351
C  -2.58379  1.73216  1.68714
H  -1.74463  1.37420  2.31551
H  -3.39323  2.04309  2.38040
H  -2.97109  0.88572  1.08333
C   0.19345  4.42331 -1.01584
H   1.06726  4.17697 -0.38063
H   0.56841  4.86141 -1.96587
H  -0.38178  5.21350 -0.49685
C  -1.72588  4.09805  1.67405
H  -0.76072  3.90216  2.18143
H  -1.60724  5.03555  1.09641
H  -2.49602  4.29119  2.45117
C   0.14929  2.22109 -2.20594
H  -0.44678  1.32185 -2.48777
H   0.44192  2.71520 -3.15446
H   1.10258  1.90413 -1.72886
H  -2.37247  2.64278 -2.54761
H  -3.90961  4.45781 -1.85476
H  -3.83292  2.38186 -0.48853
H   4.67441  2.96257  3.22745
C   0.21086  1.77376  3.97332
H  -0.61078  2.51283  3.86916
H  -0.23898  0.77089  3.81662
H   0.55898  1.81519  5.02537
H   2.66959  2.55263  4.65627
```

TS(C–D^m)

SCF (BP86) Energy = -2595.61671453
Enthalpy 0K = -2594.631854
Enthalpy 298K = -2594.573682
Free Energy 298K = -2594.723147
Lowest Frequency = -1157.6537 cm^{-1}
Second Frequency = 11.5314 cm^{-1}
SCF (BP86-D3^{BJ}) Energy = -2595.91702416
SCF (C_6H_6) Energy = -2595.65762859
SCF ($C_6H_5CH_3$) Energy = -2595.65714308
SCF (BS2) Energy = -2597.76087747

```
Ca -0.11238  0.08408 -0.17432
N  -1.49422 -1.87159 -0.04249
N   1.59633 -1.60336 -0.73324
C  -1.13699 -3.15488 -0.25264
C   0.14513 -3.59484 -0.66033
H   0.21215 -4.68405 -0.79937
C   1.39211 -2.92927 -0.81740
C  -2.14015 -4.27457  0.01400
H  -3.17433 -3.99363 -0.26206
H  -2.15754 -4.51685  1.09864
H  -1.86021 -5.19901 -0.52631
C   2.55876 -3.89026 -1.04603
H   2.27537 -4.67167 -1.77966
H   2.80368 -4.41795 -0.09985
H   3.47728 -3.38393 -1.39363
C  -2.82779 -1.60373  0.39676
C  -3.15827 -1.68255  1.78636
C  -4.46471 -1.34262  2.19422
H  -4.72784 -1.39456  3.26320
C  -5.43723 -0.94701  1.26725
H  -6.45362 -0.68905  1.60476
C  -5.10983 -0.87956 -0.09537
H  -5.88273 -0.56817 -0.81386
C  -3.81673 -1.19710 -0.55959
C  -2.12795 -2.11525  2.83557
H  -1.26524 -2.55225  2.29135
C  -2.67098 -3.20043  3.78886
H  -3.47736 -2.81047  4.44566
H  -1.86303 -3.57484  4.45218
H  -3.08562 -4.06647  3.23266
C  -1.59075 -0.91190  3.64024
H  -1.10222 -0.15864  2.98735
H  -0.83804 -1.23832  4.38861
H  -2.40838 -0.39136  4.18282
C  -3.48421 -1.11550 -2.05599
H  -2.44714 -0.71021 -2.12264
C  -3.46691 -2.49772 -2.74892
H  -2.68551 -3.16553 -2.34142
H  -3.26790 -2.37986 -3.83542
H  -4.44963 -3.00443 -2.63983
C  -4.40659 -0.16905 -2.84560
H  -5.43238 -0.58342 -2.94655
H  -4.01596 -0.02694 -3.87457
H  -4.48897  0.82948 -2.37172
C   2.94672 -1.12010 -0.74309
C   3.73018 -1.16272  0.45561
C   5.02266 -0.60027  0.44360
H   5.62506 -0.62225  1.36637
C   5.55929 -0.01923 -0.71171
H   6.57176  0.41485 -0.69863
C   4.80087 -0.00732 -1.89070
H   5.23661  0.42894 -2.80261
C   3.50103 -0.55340 -1.93809
C   3.22886 -1.82041  1.74654
H   2.22921 -2.25402  1.53567
C   3.05871 -0.80282  2.89361
H   4.03180 -0.34945  3.17786
H   2.64703 -1.30087  3.79753
H   2.38078  0.02856  2.61573
C   4.15107 -2.97683  2.19500
H   4.31120 -3.72066  1.38840
H   3.71533 -3.50649  3.06875
```

H	5.15045	-2.60194	2.50307
C	2.74621	-0.59807	-3.27188
H	1.66137	-0.57272	-3.03076
C	2.99704	-1.92185	-4.03135
H	4.07806	-2.04704	-4.25555
H	2.44691	-1.92800	-4.99660
H	2.66657	-2.80435	-3.45234
C	3.06588	0.58969	-4.20098
H	2.99449	1.56558	-3.67965
H	2.36469	0.60553	-5.06148
H	4.08910	0.51335	-4.62641
C	3.24792	3.04205	2.78317
C	1.97112	2.84336	3.35611
C	0.94092	2.35562	2.52125
H	-0.05455	2.22401	2.98787
C	1.11403	2.05520	1.14247
H	0.00362	2.24120	0.26375
C	2.41873	2.27290	0.62579
H	2.64389	2.06585	-0.43769
C	3.47122	2.75430	1.42774
H	4.47226	2.90599	0.99138
N	-1.07194	2.29556	-0.58012
C	-2.16923	2.99151	0.15735
C	-0.68778	2.83477	-1.91382
C	-3.39354	3.22120	-0.76926
C	-1.94094	3.06104	-2.80310
C	-3.02458	3.88495	-2.10014
H	-4.15683	3.81996	-0.22479
H	-1.63362	3.53778	-3.76069
H	-2.67186	4.92667	-1.93173
C	-2.61438	2.06040	1.30293
H	-1.78987	1.88480	2.02484
H	-3.45304	2.50605	1.87728
H	-2.96344	1.08284	0.91078
C	0.16876	4.13415	-1.86601
H	1.01347	4.02417	-1.15646
H	0.58682	4.36550	-2.86941
H	-0.42000	5.01523	-1.54857
C	-1.74206	4.34661	0.79528
H	-0.80411	4.23397	1.37483
H	-1.57822	5.14256	0.04368
H	-2.53091	4.71155	1.48738
C	0.17455	1.75010	-2.59038
H	-0.40008	0.80133	-2.71325
H	0.49550	2.04936	-3.60801
H	1.11447	1.55583	-2.02399
H	-2.36675	2.06585	-3.06285
H	-3.92198	3.97175	-2.75060
H	-3.85756	2.23149	-0.97661
C	1.72586	3.15314	4.81747
H	0.67580	2.94674	5.10750
H	2.38368	2.54896	5.47912
H	1.93547	4.22018	5.04782
H	4.07258	3.42732	3.40712

TS(C-D^{P})

SCF (BP86) Energy = -2595.61701237
Enthalpy 0K = -2594.632246
Enthalpy 298K = -2594.573991
Free Energy 298K = -2594.724515
Lowest Frequency = -1153.4487 cm^{-1}
Second Frequency = 10.8631 cm^{-1}
SCF (BP86-D3^{BJ}) Energy = -2595.91693932
SCF (C_6H_6) Energy = -2595.65744015
SCF ($C_6H_5CH_3$) Energy = -2595.65698992
SCF (BS2) Energy = -2597.76116679

Ca	-0.17639	0.09172	-0.13966
N	-1.68997	-1.76501	-0.04123
N	1.38462	-1.68093	-0.84874
C	-1.43543	-3.05733	-0.33027
C	-0.20555	-3.56474	-0.81379
H	-0.22398	-4.64702	-1.01011
C	1.08013	-2.98255	-0.98990
C	-2.50641	-4.11580	-0.07746
H	-3.52743	-3.74739	-0.29310
H	-2.49808	-4.41490	0.99303
H	-2.31682	-5.02708	-0.67625
C	2.16296	-4.01149	-1.31475
H	1.79557	-4.72922	-2.07570
H	2.40198	-4.60626	-0.40772
H	3.10331	-3.55492	-1.67260
C	-2.98101	-1.42756	0.47101
C	-3.25820	-1.55852	1.86832
C	-4.51824	-1.15014	2.35211
H	-4.73966	-1.24157	3.42787
C	-5.49699	-0.63643	1.49200
H	-6.47691	-0.32615	1.88823
C	-5.22234	-0.51736	0.12131
H	-5.99943	-0.11291	-0.54431
C	-3.97637	-0.89981	-0.41699
C	-2.21790	-2.11642	2.84623
H	-1.41457	-2.58660	2.24206
C	-2.79696	-3.20579	3.77298
H	-3.54314	-2.79249	4.48438
H	-1.99038	-3.66887	4.37954
H	-3.29766	-4.01171	3.19753
C	-1.55837	-0.99570	3.67870
H	-1.04482	-0.24863	3.03801
H	-0.79949	-1.41113	4.37490
H	-2.31068	-0.44469	4.28223
C	-3.70124	-0.75937	-1.92074
H	-2.64224	-0.42114	-2.01279
C	-3.80923	-2.09970	-2.68407
H	-3.06004	-2.84007	-2.34763
H	-3.64832	-1.93644	-3.77101
H	-4.81953	-2.54403	-2.55651
C	-4.58734	0.28881	-2.61806
H	-5.64320	-0.04857	-2.69232
H	-4.23174	0.45899	-3.65544
H	-4.57885	1.26378	-2.09101
C	2.76524	-1.29466	-0.89213
C	3.58954	-1.45351	0.26886
C	4.91805	-0.98390	0.22907
H	5.55272	-1.09645	1.12319
C	5.45009	-0.38350	-0.91844
H	6.49124	-0.02343	-0.92758
C	4.64923	-0.25764	-2.06220
H	5.07963	0.19278	-2.96977
C	3.31252	-0.70800	-2.08080
C	3.09247	-2.13946	1.54656
H	2.05396	-2.48362	1.35970
C	3.05088	-1.17498	2.75000
H	4.06845	-0.81812	3.01555
H	2.63482	-1.68621	3.64447
H	2.43383	-0.27797	2.54438
C	3.93927	-3.38500	1.89366

H	4.00615	-4.09509	1.04469
H	3.50103	-3.92549	2.75948
H	4.97717	-3.10391	2.17292
C	2.50598	-0.63262	-3.38244
H	1.43547	-0.54764	-3.09529
C	2.63609	-1.92915	-4.21561
H	3.69652	-2.11388	-4.49113
H	2.04967	-1.84922	-5.15589
H	2.26914	-2.81649	-3.66676
C	2.87108	0.57755	-4.26480
H	2.88768	1.52775	-3.69379
H	2.14000	0.68503	-5.09325
H	3.86941	0.45471	-4.73603
C	3.53544	2.66747	2.82168
C	2.25587	2.50807	3.39745
H	2.12550	2.68042	4.48033
C	1.15055	2.13692	2.61097
H	0.17441	2.04521	3.12310
C	1.24283	1.89634	1.21281
H	0.11878	2.20940	0.39206
C	2.54418	2.05654	0.66318
H	2.71804	1.88554	-0.41659
C	3.65963	2.42577	1.43617
H	4.64754	2.52594	0.95473
N	-0.98446	2.38477	-0.39777
C	-1.99484	3.11744	0.42350
C	-0.61839	2.96401	-1.71938
C	-3.23670	3.48436	-0.43282
C	-1.88794	3.32724	-2.53717
C	-2.87564	4.18954	-1.74445
H	-3.92917	4.10711	0.17568
H	-1.58705	3.83019	-3.48332
H	-2.43822	5.19207	-1.54046
C	-2.45915	2.15991	1.53945
H	-1.62008	1.88545	2.21209
H	-3.23660	2.63303	2.17472
H	-2.89708	1.23333	1.11389
C	0.33382	4.19381	-1.64705
H	1.19801	3.98562	-0.98455
H	0.72401	4.44794	-2.65616
H	-0.17339	5.09663	-1.25803
C	-1.44215	4.40211	1.10909
H	-0.49231	4.19158	1.63983
H	-1.25076	5.22324	0.39182
H	-2.17264	4.78544	1.85332
C	0.13159	1.85682	-2.48736
H	-0.51653	0.95962	-2.63046
H	0.43144	2.18526	-3.50248
H	1.07713	1.56583	-1.97420
H	-2.39798	2.38070	-2.82541
H	-3.79069	4.37649	-2.34750
H	-3.78200	2.54348	-0.66769
C	4.72068	3.10973	3.65077
H	4.61964	2.79784	4.71061
H	5.67262	2.69667	3.25820
H	4.82288	4.21807	3.64672

TS(C-E)

SCF (BP86) Energy = -2595.62188505
Enthalpy 0K = -2594.637644
Enthalpy 298K = -2594.579441
Free Energy 298K = -2594.731352
Lowest Frequency = -1265.1945 cm^{-1}
Second Frequency = 8.9694 cm^{-1}
SCF (BP86-D3^{BJ}) Energy = -2595.91796344
SCF (C_6H_6) Energy = -2595.66340653
SCF ($C_6H_5CH_3$) Energy = -2595.66297662
SCF (BS2) Energy = -2597.76604136

Ca	0.01124	-0.02639	-0.07761
N	2.10798	-0.79936	-1.00361
N	-0.82978	-2.08588	-1.01297
C	2.22233	-1.80548	-1.89225
C	1.16799	-2.64250	-2.34512
H	1.48867	-3.36914	-3.10572
C	-0.16215	-2.85446	-1.89520
C	3.59487	-2.18734	-2.44142
H	3.50443	-2.65133	-3.44257
H	4.07154	-2.93834	-1.77484
H	4.28845	-1.32801	-2.49925
C	-0.80571	-4.12807	-2.43787
H	-0.58553	-4.97982	-1.75836
H	-0.39189	-4.38785	-3.43134
H	-1.90691	-4.05582	-2.50931
C	3.31257	-0.17294	-0.54411
C	3.73764	1.06877	-1.11709
C	4.90071	1.68572	-0.61228
H	5.23996	2.63716	-1.05078
C	5.63615	1.11641	0.43689
H	6.54096	1.61739	0.81614
C	5.20903	-0.09353	0.99909
H	5.78708	-0.53779	1.82593
C	4.05898	-0.75900	0.52681
C	2.97196	1.71871	-2.27436
H	1.92755	1.33688	-2.22013
C	3.52390	1.29535	-3.65482
H	4.58923	1.59315	-3.76000
H	2.95217	1.78626	-4.47077
H	3.46054	0.20153	-3.81264
C	2.91951	3.25621	-2.18264
H	2.58973	3.60205	-1.18142
H	2.21489	3.66442	-2.93636
H	3.90994	3.71648	-2.38435
C	3.63759	-2.07711	1.18567
H	2.80871	-2.50098	0.58153
C	4.77721	-3.11814	1.20524
H	5.19524	-3.28989	0.19202
H	4.41031	-4.09204	1.59268
H	5.61584	-2.79959	1.86021
C	3.09427	-1.84693	2.61293
H	3.86762	-1.39880	3.27254
H	2.77122	-2.80408	3.07441
H	2.22173	-1.16011	2.61828
C	-2.10717	-2.54708	-0.55505
C	-3.30800	-2.02691	-1.13638
C	-4.54979	-2.46301	-0.63031
H	-5.47950	-2.07439	-1.07450
C	-4.62936	-3.37978	0.42744
H	-5.61095	-3.70437	0.80771
C	-3.45024	-3.87804	0.99682
H	-3.51552	-4.59690	1.82990
C	-2.18166	-3.48455	0.52340
C	-3.26747	-1.03403	-2.30288
H	-2.27881	-0.52471	-2.25450
C	-4.36183	0.04801	-2.21985
H	-5.37201	-0.36944	-2.41670
H	-4.18459	0.83596	-2.98077

```
H  -4.39108  0.53390 -1.22299
C  -3.33263 -1.73958 -3.67676
H  -2.48555 -2.43567 -3.82954
H  -3.30739 -0.99405 -4.49967
H  -4.27374 -2.32205 -3.77522
C  -0.92506 -4.05624  1.18898
H  -0.05479 -3.75786  0.56833
C  -0.93919 -5.59810  1.25551
H  -1.73653 -5.97589  1.93022
H   0.02769 -5.98084  1.64513
H  -1.10779 -6.05105  0.25680
C  -0.71035 -3.45673  2.59595
H  -0.61592 -2.35052  2.56456
H   0.21326 -3.85903  3.06339
H  -1.56099 -3.69307  3.27013
C  -2.69922  6.18070 -1.25068
C  -1.30398  6.00487 -1.21986
C  -0.74691  4.71700 -1.16839
C  -1.56146  3.55277 -1.14584
H  -0.86507  1.93289  0.40393
C  -2.96761  3.75498 -1.18219
H  -3.63454  2.87737 -1.18581
C  -3.52554  5.04251 -1.23361
H  -4.62165  5.15663 -1.26705
N  -0.65887  1.44463  1.69126
C   0.38014  2.25071  2.39213
C  -1.96250  1.24919  2.38648
C   0.58721  1.71377  3.83543
C  -1.72312  0.72633  3.83006
C  -0.72657  1.58694  4.61600
H   1.31047  2.36381  4.37636
H  -2.69511  0.65335  4.36686
H  -1.15715  2.59368  4.81292
C   1.69172  2.05599  1.60785
H   1.59913  2.42363  0.56322
H   2.53420  2.61331  2.06552
H   2.00588  0.98892  1.58864
C  -2.87371  2.51008  2.42707
H  -2.95023  2.97863  1.42507
H  -3.89804  2.23288  2.75654
H  -2.50817  3.28378  3.12807
C   0.10026  3.78095  2.43281
H  -0.18112  4.16115  1.42995
H  -0.71480  4.05107  3.13003
H   1.00732  4.32882  2.76714
C  -2.72714  0.16933  1.59773
H  -2.17044 -0.79304  1.57120
H  -3.71122 -0.05659  2.05663
H  -2.92915  0.49586  0.55519
H  -1.31994 -0.30986  3.75969
H  -0.53740  1.13943  5.61591
H   1.05635  0.70562  3.76415
H  -3.13644  7.19063 -1.29415
H  -0.63721  6.88266 -1.24237
C  -0.97183  2.18948 -1.03268
H   0.01735  2.16827 -1.55423
H  -1.63288  1.45279 -1.55275
H   0.34943  4.60333 -1.16129
```

D°

SCF (BP86) Energy = -2595.63542369
Enthalpy 0K = -2594.646485
Enthalpy 298K = -2594.587237
Free Energy 298K = -2594.740704
Lowest Frequency = 15.9210 cm^{-1}
Second Frequency = 24.7539 cm^{-1}
SCF (BP86-D3^{BJ}) Energy = -2595.93719247
SCF (C_6H_6) Energy = -2595.67642131
SCF ($C_6H_5CH_3$) Energy = -2595.67598634
SCF (BS2) Energy = -2597.78076540

```
Ca -0.02140  0.48249  0.01060
N  -1.39912 -0.83428  1.46560
N   1.76330 -0.61082  1.23504
C  -1.00706 -1.16267  2.70797
C   0.33197 -1.10407  3.18140
H   0.42852 -1.35130  4.24949
C   1.58564 -0.96484  2.52076
C  -2.02798 -1.63357  3.74054
H  -2.93635 -2.05311  3.26737
H  -2.35042 -0.77798  4.37250
H  -1.59173 -2.39226  4.41913
C   2.78758 -1.31230  3.39674
H   2.61996 -0.96647  4.43602
H   3.72972 -0.87458  3.01742
H   2.92870 -2.41338  3.44231
C  -2.72978 -1.07395  1.01015
C  -3.79915 -0.16508  1.29472
C  -5.05643 -0.38743  0.69310
H  -5.88143  0.31261  0.90405
C  -5.28242 -1.47517 -0.15926
H  -6.27259 -1.62635 -0.61800
C  -4.23990 -2.37931 -0.41348
H  -4.42748 -3.24517 -1.06719
C  -2.96338 -2.20583  0.15741
C  -3.63316  1.02964  2.23850
H  -2.61803  0.96131  2.68094
C  -4.65344  0.98806  3.39779
H  -5.69173  1.14411  3.03485
H  -4.43927  1.79281  4.13242
H  -4.63175  0.01792  3.93502
C  -3.71184  2.38201  1.49811
H  -2.89003  2.49755  0.76268
H  -3.62482  3.22474  2.21586
H  -4.67848  2.49584  0.96226
C  -1.86090 -3.24342 -0.07366
H  -0.89202 -2.69628 -0.02925
C  -1.83333 -4.28436  1.06947
H  -1.67624 -3.80900  2.05691
H  -1.01434 -5.01871  0.91450
H  -2.79153 -4.84549  1.11063
C  -1.94481 -3.96077 -1.43291
H  -2.82393 -4.63707 -1.49435
H  -1.04446 -4.58984 -1.59082
H  -2.01302 -3.24594 -2.27871
C   3.05605 -0.76972  0.64485
C   3.86712  0.38066  0.37157
C   5.11053  0.19941 -0.26846
H   5.74152  1.07866 -0.47126
C   5.56381 -1.07156 -0.64944
H   6.54000 -1.18830 -1.14678
C   4.76167 -2.19051 -0.39129
H   5.11526 -3.18955 -0.69566
C   3.51188 -2.06998  0.25126
C   3.42871  1.78389  0.79614
H   2.31502  1.80434  0.80463
C   3.87617  2.89738 -0.16881
```

```
H   4.97079  3.08280 -0.11864
H   3.36468  3.84656  0.09026
H   3.62368  2.66024 -1.22354
C   3.88332  2.11463  2.23508
H   3.48516  1.38977  2.97202
H   3.52874  3.12449  2.52980
H   4.99225  2.10236  2.31134
C   2.67495 -3.33458  0.47518
H   1.79276 -3.05200  1.08511
C   3.44746 -4.42600  1.24688
H   4.30006 -4.82327  0.65573
H   2.78285 -5.28415  1.48216
H   3.85904 -4.04130  2.20234
C   2.14337 -3.89898 -0.86053
H   1.52478 -3.15415 -1.40263
H   1.51547 -4.79917 -0.68855
H   2.97526 -4.19398 -1.53539
C   0.32458  5.82687  0.60879
C   0.24607  4.98042  1.72932
C   0.10714  3.58065  1.58189
C   0.04539  2.95946  0.29006
H  -0.06717  1.76750 -2.44099
C   0.13266  3.86459 -0.80236
H   0.10369  3.48657 -1.84890
C   0.26793  5.26358 -0.67470
H   0.33294  5.90646 -1.56959
N  -0.37474  0.79817 -2.64068
C  -1.78372  0.91521 -3.17797
C   0.69285  0.17397 -3.50379
C  -2.21996 -0.46144 -3.72946
C   0.16744 -1.18183 -4.03126
C  -1.20737 -1.07696 -4.70327
H  -3.21820 -0.35282 -4.20434
H   0.92255 -1.61341 -4.72240
H  -1.14122 -0.47308 -5.63504
C  -2.67421  1.30084 -1.98338
H  -2.33122  2.24862 -1.51709
H  -3.72607  1.44159 -2.30451
H  -2.68660  0.50307 -1.21039
C   1.16299  1.08131 -4.66922
H   1.42107  2.09677 -4.30122
H   2.07548  0.65513 -5.13521
H   0.40791  1.19293 -5.46930
C  -1.93835  2.01925 -4.25494
H  -1.51986  2.98338 -3.89691
H  -1.44925  1.76944 -5.21500
H  -3.01436  2.18591 -4.46851
C   1.90254 -0.06267 -2.58351
H   1.66820 -0.78014 -1.76791
H   2.75768 -0.49963 -3.13698
H   2.26441  0.88559 -2.13065
H   0.09020 -1.88516 -3.17149
H  -1.55052 -2.08463 -5.02116
H  -2.36046 -1.15204 -2.86841
H   0.43337  6.91620  0.74083
C  -0.00072  2.73588  2.84132
H  -1.05957  2.47066  3.05803
H   0.55070  1.77497  2.75465
H   0.38741  3.26233  3.73851
H   0.29452  5.42081  2.74170
```

D[m]

SCF (BP86) Energy = -2595.63048319
Enthalpy 0K = -2594.640659
Enthalpy 298K = -2594.581854
Free Energy 298K = -2594.734258
Lowest Frequency = 12.3428 cm^{-1}
Second Frequency = 24.1571 cm^{-1}
SCF (BP86-D3^{BJ}) Energy = -2595.92580083
SCF (C_6H_6) Energy = -2595.67297227
SCF ($C_6H_5CH_3$) Energy = -2595.67255598
SCF (BS2) Energy = -2597.77704165

```
Ca  0.02374 -0.02796 -0.08069
N  -1.23200 -2.06322  0.21299
N   1.82758 -1.62712 -0.46338
C  -0.76386 -3.32823  0.20460
C   0.56816 -3.70805 -0.08635
H   0.73379 -4.79519 -0.05733
C   1.75034 -2.96284 -0.35513
C  -1.69519 -4.48132  0.57158
H  -2.71851 -4.33932  0.17336
H  -1.79592 -4.55628  1.67587
H  -1.29831 -5.44826  0.20813
C   2.99829 -3.83425 -0.48793
H   2.77951 -4.73163 -1.10105
H   3.31101 -4.20152  0.51230
H   3.85523 -3.29447 -0.93084
C  -2.60806 -1.84620  0.52068
C  -3.04643 -1.78362  1.88169
C  -4.40185 -1.49818  2.14552
H  -4.74883 -1.44457  3.18997
C  -5.31900 -1.28762  1.10741
H  -6.37481 -1.07082  1.33504
C  -4.88532 -1.35420 -0.22521
H  -5.61515 -1.19003 -1.03235
C  -3.53987 -1.62721 -0.54796
C  -2.07394 -2.00204  3.04584
H  -1.16674 -2.48839  2.63188
C  -2.64231 -2.93483  4.13491
H  -3.49229 -2.47114  4.67929
H  -1.86237 -3.16752  4.89000
H  -3.00314 -3.89405  3.70887
C  -1.61780 -0.66226  3.66214
H  -1.09984 -0.01975  2.91963
H  -0.90736 -0.83165  4.49864
H  -2.48084 -0.08533  4.05746
C  -3.08956 -1.70602 -2.01300
H  -2.06842 -1.25779 -2.05235
C  -2.94965 -3.15992 -2.51991
H  -2.17874 -3.72578 -1.96459
H  -2.66022 -3.16898 -3.59239
H  -3.91490 -3.70189 -2.42594
C  -3.98795 -0.91587 -2.98172
H  -4.97929 -1.39995 -3.11136
H  -3.52035 -0.87014 -3.98725
H  -4.16366  0.12328 -2.63785
C   3.10778 -1.00664 -0.63087
C   3.97451 -0.80166  0.49137
C   5.17542 -0.08865  0.29665
H   5.84049  0.08239  1.15870
C   5.54308  0.40561 -0.96025
H   6.48536  0.96231 -1.08593
C   4.70571  0.17717 -2.06140
H   5.01063  0.54918 -3.05125
C   3.49076 -0.52611 -1.92735
C   3.67009 -1.34997  1.89058
```

H	2.70617	-1.89747	1.83382
C	3.50381	-0.23175	2.94125
H	4.44306	0.34829	3.06557
H	3.25068	-0.66832	3.93121
H	2.70434	0.48020	2.65783
C	4.75451	-2.35088	2.35280
H	4.92474	-3.15990	1.61404
H	4.46486	-2.82095	3.31668
H	5.72833	-1.84149	2.51567
C	2.63663	-0.82312	-3.16581
H	1.57233	-0.80173	-2.83817
C	2.89296	-2.24465	-3.71760
H	3.95836	-2.36382	-4.00903
H	2.27057	-2.43246	-4.61865
H	2.65280	-3.02908	-2.97614
C	2.81201	0.20220	-4.30226
H	2.73296	1.24820	-3.94396
H	2.04158	0.04467	-5.08582
H	3.79919	0.09461	-4.79998
C	2.50745	4.03277	2.39154
C	1.38566	3.52201	3.08297
C	0.67244	2.45086	2.49312
H	-0.20169	2.08027	3.06743
C	1.01054	1.83585	1.25424
H	-0.54345	2.46880	-0.15241
C	2.15678	2.39737	0.62005
H	2.53275	1.97691	-0.33634
C	2.89179	3.47190	1.16386
H	3.77428	3.86770	0.63185
N	-1.34118	2.27281	-0.80066
C	-2.56396	2.85423	-0.13843
C	-0.94981	2.74788	-2.16890
C	-3.74607	2.83412	-1.13460
C	-2.18922	2.72937	-3.09513
C	-3.40197	3.45016	-2.49568
H	-4.61154	3.35156	-0.66803
H	-1.90641	3.16513	-4.07770
H	-3.19788	4.53876	-2.39312
C	-2.89481	1.94515	1.05948
H	-2.06355	1.94708	1.79402
H	-3.80382	2.30400	1.58493
H	-3.08664	0.90323	0.73130
C	-0.26943	4.14168	-2.17496
H	0.55181	4.17916	-1.42908
H	0.16646	4.35397	-3.17396
H	-0.97492	4.96183	-1.94387
C	-2.32827	4.28710	0.40760
H	-1.41382	4.31926	1.03589
H	-2.22129	5.04619	-0.39077
H	-3.18503	4.59694	1.04203
C	0.07528	1.72750	-2.69923
H	-0.37261	0.70887	-2.75191
H	0.40626	1.97948	-3.72651
H	0.99406	1.71219	-2.07034
H	-2.46759	1.66928	-3.28630
H	-4.27018	3.36835	-3.18432
H	-4.05671	1.77635	-1.28413
C	0.97710	4.10224	4.42107
H	0.01337	3.67859	4.77070
H	1.73706	3.89159	5.20564
H	0.86588	5.20716	4.37531
H	3.08261	4.87130	2.82144

D^{P}

SCF (BP86) Energy = -2595.63065069
Enthalpy 0K = -2594.641025
Enthalpy 298K = -2594.582089
Free Energy 298K = -2594.736149
Lowest Frequency = 11.0992 cm^{-1}
Second Frequency = 12.9966 cm^{-1}
SCF (BP86-D3^{BJ}) Energy = -2595.92542948
SCF (C_6H_6) Energy = -2595.67307938
SCF ($C_6H_5CH_3$) Energy = -2595.67265624
SCF (BS2) Energy = -2597.77715053

Ca	-0.03555	-0.05068	-0.05263
N	-1.52015	-1.93373	0.20346
N	1.54404	-1.83709	-0.56535
C	-1.21108	-3.24411	0.12564
C	0.05322	-3.76780	-0.23582
H	0.08607	-4.86712	-0.25976
C	1.30793	-3.15776	-0.51516
C	-2.26051	-4.29183	0.48974
H	-3.27523	-4.00936	0.14874
H	-2.32071	-4.40469	1.59387
H	-2.00155	-5.28202	0.06890
C	2.43615	-4.16517	-0.73176
H	2.08983	-5.00080	-1.37272
H	2.73526	-4.61274	0.23952
H	3.33723	-3.71231	-1.18445
C	-2.84831	-1.56528	0.57174
C	-3.22805	-1.50099	1.95005
C	-4.52822	-1.06320	2.27578
H	-4.82954	-1.00690	3.33415
C	-5.44833	-0.70464	1.28161
H	-6.46090	-0.36967	1.55741
C	-5.07321	-0.77514	-0.06843
H	-5.80513	-0.49499	-0.84115
C	-3.78346	-1.19744	-0.45198
C	-2.24948	-1.88061	3.06668
H	-1.42733	-2.46232	2.60120
C	-2.89139	-2.77258	4.14887
H	-3.65492	-2.22555	4.74186
H	-2.12013	-3.12837	4.86383
H	-3.38652	-3.66286	3.70837
C	-1.60399	-0.63151	3.70385
H	-1.03471	-0.03450	2.96072
H	-0.89135	-0.91800	4.50581
H	-2.37139	0.03621	4.15012
C	-3.39976	-1.28229	-1.93537
H	-2.32566	-0.98823	-2.00012
C	-3.49412	-2.72063	-2.49478
H	-2.79747	-3.41457	-1.98871
H	-3.24594	-2.73157	-3.57753
H	-4.52514	-3.11836	-2.38010
C	-4.20524	-0.33199	-2.83992
H	-5.26079	-0.66090	-2.94732
H	-3.77045	-0.31396	-3.86097
H	-4.21459	0.70681	-2.45287
C	2.88637	-1.36885	-0.74082
C	3.79658	-1.32727	0.36492
C	5.07162	-0.75750	0.16986
H	5.77170	-0.71182	1.01987
C	5.46932	-0.25014	-1.07262
H	6.47018	0.19251	-1.19893
C	4.58546	-0.31865	-2.15893
H	4.91170	0.06280	-3.13831

C	3.29604	-0.87347	-2.02343
C	3.45725	-1.90157	1.74563
H	2.43279	-2.32526	1.68993
C	3.45139	-0.82383	2.85049
H	4.45691	-0.36829	2.97521
H	3.16890	-1.27441	3.82611
H	2.73892	-0.00708	2.62270
C	4.42067	-3.04778	2.13248
H	4.47477	-3.83478	1.35338
H	4.09626	-3.52534	3.08148
H	5.45283	-2.66887	2.29192
C	2.38243	-0.99776	-3.24874
H	1.33759	-0.85722	-2.88936
C	2.44063	-2.41089	-3.87365
H	3.47473	-2.65045	-4.20146
H	1.77869	-2.47219	-4.76393
H	2.11823	-3.19428	-3.16294
C	2.66097	0.05270	-4.34071
H	2.72307	1.08119	-3.93190
H	1.85925	0.03231	-5.10828
H	3.61505	-0.15316	-4.87094
C	3.01206	3.60516	2.49709
C	1.83519	3.19062	3.16067
H	1.59446	3.61944	4.15059
C	0.97263	2.24092	2.58040
H	0.07425	1.96799	3.16918
C	1.20599	1.62461	1.31655
H	-0.30166	2.50149	-0.00195
C	2.40641	2.06505	0.68624
H	2.71638	1.63337	-0.28883
C	3.28319	3.01984	1.24342
H	4.20112	3.30731	0.69989
N	-1.13243	2.43517	-0.63492
C	-2.26228	3.12270	0.08681
C	-0.71857	2.92625	-1.99040
C	-3.46090	3.28598	-0.87607
C	-1.97187	3.09524	-2.88226
C	-3.07816	3.92213	-2.21737
H	-4.25019	3.87634	-0.36331
H	-1.66224	3.54177	-3.85184
H	-2.74542	4.97339	-2.07155
C	-2.67001	2.20205	1.25148
H	-1.82909	2.07387	1.96347
H	-3.52048	2.63658	1.81624
H	-2.98698	1.20570	0.88153
C	0.11897	4.23131	-1.95342
H	0.95520	4.13906	-1.22934
H	0.55462	4.43868	-2.95357
H	-0.48122	5.11547	-1.66733
C	-1.84905	4.49155	0.68898
H	-0.92301	4.38894	1.29229
H	-1.67300	5.27024	-0.07776
H	-2.64936	4.86747	1.36037
C	0.16914	1.82087	-2.59307
H	-0.39591	0.86482	-2.68004
H	0.50597	2.08201	-3.61618
H	1.09212	1.66896	-1.98903
H	-2.37649	2.08479	-3.11309
H	-3.96538	3.97471	-2.88429
H	-3.89465	2.27887	-1.06409
C	3.93202	4.63978	3.10860
H	4.18590	4.39120	4.16134
H	4.88144	4.72797	2.54221
H	3.45984	5.64718	3.12479

E

SCF (BP86) Energy = -2595.64566756
Enthalpy 0K = -2594.655791
Enthalpy 298K = -2594.597324
Free Energy 298K = -2594.749155
Lowest Frequency = 10.6471 cm^{-1}
Second Frequency = 21.0850 cm^{-1}
SCF (BP86-D3^{BJ}) Energy = -2595.94368490
SCF (C_6H_6) Energy = -2595.766858
SCF ($C_6H_5CH_3$) Energy = -2595.707605
SCF (BS2) Energy = -2597.79097521

Ca	0.01034	0.21490	-0.47923
N	1.52196	-1.62118	-0.93197
N	-1.71671	-1.41874	-0.94768
C	1.14667	-2.62282	-1.74910
C	-0.17579	-2.88125	-2.20483
H	-0.22384	-3.71124	-2.92564
C	-1.46035	-2.45590	-1.76738
C	2.16580	-3.66231	-2.21407
H	1.93618	-3.99938	-3.24419
H	2.11148	-4.56093	-1.56263
H	3.20715	-3.29309	-2.17689
C	-2.59870	-3.35185	-2.25386
H	-2.69127	-4.23885	-1.59093
H	-2.38710	-3.73254	-3.27243
H	-3.57940	-2.84135	-2.25379
C	2.85570	-1.63079	-0.41029
C	3.86278	-0.79413	-0.99618
C	5.15571	-0.79486	-0.43405
H	5.93759	-0.16264	-0.88164
C	5.47237	-1.58170	0.68290
H	6.49043	-1.56460	1.10354
C	4.48161	-2.38618	1.25798
H	4.72980	-3.00245	2.13789
C	3.17331	-2.43272	0.73203
C	3.56160	0.07729	-2.21983
H	2.49419	0.38264	-2.14276
C	3.70320	-0.69837	-3.54938
H	4.73600	-1.09082	-3.66894
H	3.49159	-0.03037	-4.41099
H	3.00356	-1.55294	-3.61212
C	4.40583	1.36420	-2.28264
H	4.37932	1.92966	-1.32877
H	4.02656	2.02936	-3.08573
H	5.47046	1.15230	-2.51914
C	2.13989	-3.33805	1.40993
H	1.20520	-3.28162	0.81442
C	2.58805	-4.81563	1.44204
H	2.84313	-5.19099	0.43015
H	1.78283	-5.46102	1.85262
H	3.48398	-4.95778	2.08330
C	1.81037	-2.84875	2.83610
H	2.70874	-2.86561	3.48932
H	1.04001	-3.49315	3.30955
H	1.42427	-1.80825	2.83207
C	-3.04544	-1.27453	-0.43265
C	-3.93840	-0.30864	-1.00422
C	-5.22496	-0.16038	-0.44704
H	-5.91976	0.57268	-0.88446
C	-5.64363	-0.92476	0.65167
H	-6.65432	-0.79055	1.06901

```
C  -4.76361 -1.85792  1.21252
H  -5.09158 -2.45678  2.07818
C  -3.46776 -2.05506  0.69080
C  -3.52595  0.54081 -2.21017
H  -2.42828  0.70312 -2.12876
C  -4.19598  1.92735 -2.24631
H  -5.27779  1.86050 -2.49020
H  -3.72888  2.55629 -3.03204
H  -4.10298  2.46279 -1.27916
C  -3.76413 -0.18447 -3.55417
H  -3.18163 -1.12184 -3.63200
H  -3.46483  0.46579 -4.40327
H  -4.83897 -0.43668 -3.68101
C  -2.55736 -3.09161  1.35759
H  -1.62640 -3.15554  0.75723
C  -3.19289 -4.49870  1.38792
H  -4.09286 -4.52851  2.03846
H  -2.47356 -5.24500  1.78669
H  -3.50471 -4.83216  0.37712
C  -2.15870 -2.65484  2.78337
H  -1.63784 -1.67481  2.78047
H  -1.47857 -3.39746  3.25130
H  -3.04859 -2.55436  3.44071
C   0.42328  6.08212 -0.73953
C   1.57808  5.33950 -1.05792
C   1.48194  4.03240 -1.55905
C   0.22218  3.38556 -1.77688
H   0.11109  2.75032  0.70407
C  -0.93130  4.17157 -1.45246
H  -1.93016  3.74255 -1.63935
C  -0.83260  5.47935 -0.95232
H  -1.75573  6.04523 -0.73992
N   0.10439  2.05071  1.47278
C   1.42936  2.19355  2.18613
C  -1.16836  2.28998  2.25159
C   1.38416  1.36380  3.49009
C  -1.12039  1.45460  3.55195
C   0.16222  1.67040  4.36343
H   2.33073  1.52604  4.04830
H  -2.02403  1.68369  4.15610
H   0.20940  2.71205  4.75001
C   2.48964  1.60562  1.23817
H   2.48753  2.13992  0.26419
H   3.50801  1.69910  1.66628
H   2.33336  0.51894  1.06356
C  -1.43106  3.78557  2.56074
H  -1.31340  4.40275  1.64585
H  -2.47017  3.91584  2.92830
H  -0.75668  4.19442  3.33600
C   1.81837  3.66526  2.47458
H   1.71287  4.28736  1.56124
H   1.21041  4.12666  3.27503
H   2.87883  3.71582  2.79826
C  -2.31649  1.78872  1.35817
H  -2.24834  0.69623  1.16591
H  -3.30205  1.95230  1.83879
H  -2.32721  2.32947  0.38799
H  -1.19217  0.37725  3.27931
H   0.16061  1.01439  5.25996
H   1.36414  0.28429  3.21762
H   0.49970  7.11085 -0.35460
H   2.57522  5.79345 -0.92748
C   0.12045  2.00442 -2.25813
H   0.98700  1.71042 -2.88964
H  -0.80938  1.82836 -2.84208
H   2.40457  3.49191 -1.82890
```

TS(A–A′)

SCF (BP86) Energy = -2556.35759551
Enthalpy 0K = -2555.396846
Enthalpy 298K = -2555.339960
Free Energy 298K = -2555.490582
Lowest Frequency = -14.6759 cm^{-1}
Second Frequency = 13.0744 cm^{-1}
SCF (BP86-D3^{BJ}) Energy = -2556.63579073
SCF (C_6H_6) Energy = -2556.39908819
SCF ($C_6H_5CH_3$) Energy = -2556.39859444
SCF (BS2) Energy = -2558.46227482

```
Ca  0.57885 -0.61339 -0.49591
N   1.02435  1.70305 -0.78376
N  -1.67346 -0.20653 -1.15246
C   0.71709  1.79158 -2.08353
C  -0.20417  0.92679 -2.77584
H  -0.13301  1.04509 -3.86848
C  -1.39410  0.21848 -2.39320
C   1.34666  2.85775 -2.97560
H   1.67005  2.41283 -3.93855
H   2.20905  3.35658 -2.49607
H   0.59344  3.63561 -3.22263
C  -2.34789 -0.05243 -3.55459
H  -1.79751 -0.53583 -4.38819
H  -2.75811  0.89826 -3.95375
H  -3.19379 -0.70024 -3.25924
C   1.81065  2.70864 -0.14457
C   1.20419  3.93183  0.28286
C   2.00334  4.90389  0.92006
H   1.53980  5.85091  1.24157
C   3.36575  4.69222  1.15770
H   3.97362  5.46589  1.65299
C   3.94592  3.47677  0.77026
H   5.01452  3.30706  0.97063
C   3.19808  2.46962  0.12670
C  -0.28829  4.22078  0.10250
H  -0.74998  3.32435 -0.36172
C  -0.53997  5.42233 -0.83482
H  -0.14158  6.36439 -0.40090
H  -1.62784  5.56956 -1.00379
H  -0.05696  5.28456 -1.82334
C  -0.97837  4.44765  1.46513
H  -0.85078  3.57588  2.13847
H  -2.06673  4.61707  1.33336
H  -0.56533  5.33643  1.98755
C   3.89688  1.17572 -0.30173
H   3.14127  0.35393 -0.27599
C   4.42565  1.25642 -1.75138
H   3.61712  1.45465 -2.48152
H   4.91911  0.30505 -2.04283
H   5.17398  2.07186 -1.84901
C   5.03359  0.75159  0.64682
H   5.90531  1.43677  0.58384
H   5.39979 -0.25890  0.37321
H   4.70112  0.72063  1.70459
C  -2.97907 -0.64921 -0.79324
C  -4.04087  0.29738 -0.61643
C  -5.30486 -0.16436 -0.19321
H  -6.12179  0.56333 -0.05878
```

C	-5.54527	-1.51841	0.06530
H	-6.54020	-1.85662	0.39547
C	-4.50187	-2.43952	-0.10075
H	-4.69162	-3.50536	0.09744
C	-3.21919	-2.03776	-0.52552
C	-3.86292	1.80126	-0.84633
H	-2.80331	1.97913	-1.12365
C	-4.14622	2.60690	0.43988
H	-5.20089	2.49086	0.76816
H	-3.97329	3.69034	0.26859
H	-3.49874	2.28338	1.27943
C	-4.74862	2.31809	-2.00221
H	-4.57755	1.75502	-2.94146
H	-4.54527	3.39123	-2.20422
H	-5.82785	2.22672	-1.75460
C	-2.14713	-3.10268	-0.76384
H	-1.14667	-2.63431	-0.60670
C	-2.18500	-3.60645	-2.22438
H	-3.16368	-4.08334	-2.44576
H	-1.38935	-4.35977	-2.40478
H	-2.04341	-2.78196	-2.95107
C	-2.22178	-4.29038	0.21281
H	-2.24412	-3.95315	1.26929
H	-1.33633	-4.94506	0.08169
H	-3.11900	-4.92068	0.03608
C	-0.62084	0.25936	5.04939
C	-1.56145	-0.75463	5.30737
H	-1.59665	-1.23284	6.29955
C	-2.45791	-1.15740	4.30061
H	-3.19614	-1.94946	4.50469
C	-2.41613	-0.54752	3.03332
H	-3.11874	-0.85667	2.24304
C	-1.47264	0.46586	2.77601
H	-1.44785	0.94949	1.78492
C	-0.57564	0.87020	3.78309
H	0.16143	1.66363	3.57965
N	1.82358	-2.44743	-0.15111
C	2.18166	-2.84795	1.22041
C	2.45827	-3.15319	-1.27503
C	3.72507	-2.96043	1.40052
C	4.00192	-3.25368	-1.08586
C	4.38073	-3.80181	0.29689
H	3.96881	-3.37165	2.40606
H	4.45380	-3.87340	-1.89313
H	4.06678	-4.86617	0.38615
H	0.07978	0.57554	5.83883
C	1.66265	-1.72690	2.15043
H	0.55097	-1.64130	2.09908
H	1.89846	-1.91467	3.21829
H	2.12111	-0.74500	1.88839
C	1.88262	-4.57535	-1.55642
H	0.77569	-4.52907	-1.61381
H	2.26523	-4.98642	-2.51664
H	2.14288	-5.30319	-0.76396
C	1.51096	-4.16802	1.70870
H	0.41880	-4.13042	1.52208
H	1.90758	-5.06247	1.19037
H	1.67233	-4.32395	2.79804
C	2.17751	-2.30876	-2.54059
H	2.59932	-1.28302	-2.43736
H	2.62413	-2.75370	-3.45436
H	1.08103	-2.23147	-2.73432
H	4.42785	-2.22938	-1.19102
H	5.48606	-3.80415	0.42035
H	4.15166	-1.93361	1.36334

A′

SCF (BP86) Energy = -2556.35955440
Enthalpy 0K = -2555.398129
Enthalpy 298K = -2555.340738
Free Energy 298K = -2555.491275
Lowest Frequency = 10.5751 cm^{-1}
Second Frequency = 18.6856 cm^{-1}
SCF (BP86-D3^{BJ}) Energy = -2556.65071881
SCF (C_6H_6) Energy = -2556.39802502
SCF ($C_6H_5CH_3$) Energy = -2556.39755135
SCF (BS2) Energy = -2558.46300513

Ca	0.36765	-0.58607	0.02342
N	0.91549	1.56499	-0.90225
N	-1.81117	-0.22286	-0.92592
C	0.49561	1.64117	-2.17612
C	-0.56449	0.87299	-2.74787
H	-0.63495	0.99901	-3.84009
C	-1.68030	0.16856	-2.20628
C	1.15832	2.59933	-3.16477
H	1.51037	2.03436	-4.05306
H	2.01684	3.13802	-2.72338
H	0.42825	3.34775	-3.53642
C	-2.76147	-0.15802	-3.23623
H	-2.35393	-0.87860	-3.97726
H	-3.06309	0.74475	-3.80483
H	-3.66090	-0.60590	-2.77556
C	1.77658	2.56040	-0.34866
C	1.25787	3.84803	0.01398
C	2.11695	4.78789	0.62169
H	1.71821	5.77884	0.89379
C	3.45826	4.49371	0.88836
H	4.11171	5.24237	1.36372
C	3.96024	3.23242	0.54130
H	5.01696	3.00454	0.74603
C	3.15249	2.25414	-0.07430
C	-0.19809	4.25978	-0.22722
H	-0.73600	3.37820	-0.63435
C	-0.30708	5.40065	-1.26429
H	0.16299	6.33341	-0.88534
H	-1.37087	5.63101	-1.48575
H	0.19272	5.14345	-2.21899
C	-0.89914	4.67456	1.08427
H	-0.88802	3.86076	1.83710
H	-1.95819	4.94534	0.89360
H	-0.41274	5.56097	1.54365
C	3.78593	0.92645	-0.49964
H	3.00662	0.12881	-0.43962
C	4.25622	0.97593	-1.97091
H	3.42457	1.20281	-2.66508
H	4.69516	0.00245	-2.27577
H	5.03276	1.75914	-2.10628
C	4.94973	0.47999	0.40386
H	5.83851	1.13693	0.29461
H	5.27372	-0.54365	0.12658
H	4.66565	0.46915	1.47644
C	-3.08074	-0.62022	-0.40585
C	-4.06170	0.37282	-0.06945
C	-5.28663	-0.03659	0.49735
H	-6.04177	0.72640	0.74655
C	-5.56611	-1.38388	0.75445

H	-6.53007	-1.68077	1.19728
C	-4.60114	-2.34911	0.44042
H	-4.81977	-3.41031	0.63684
C	-3.36158	-2.00038	-0.13537
C	-3.83957	1.87057	-0.30361
H	-2.77693	2.00922	-0.59475
C	-4.09197	2.69865	0.97443
H	-5.15056	2.63367	1.30347
H	-3.87504	3.77162	0.79278
H	-3.45845	2.36094	1.81972
C	-4.71645	2.40461	-1.45899
H	-4.54934	1.84468	-2.39985
H	-4.50000	3.47617	-1.65602
H	-5.79632	2.32159	-1.21012
C	-2.39383	-3.12042	-0.52084
H	-1.37387	-2.68150	-0.61663
C	-2.76360	-3.71603	-1.89822
H	-3.77580	-4.17343	-1.86727
H	-2.04197	-4.50582	-2.19600
H	-2.77004	-2.94415	-2.69270
C	-2.30173	-4.23674	0.53663
H	-2.08746	-3.82992	1.54647
H	-1.48910	-4.94481	0.27590
H	-3.23959	-4.82795	0.60268
C	0.69447	0.78584	4.27842
C	-0.09078	-0.21442	4.88000
H	0.20068	-0.62625	5.85944
C	-1.24566	-0.69301	4.23394
H	-1.85817	-1.47639	4.70767
C	-1.62485	-0.16480	2.98612
H	-2.53681	-0.52211	2.48128
C	-0.84084	0.84434	2.38697
H	-1.16331	1.28937	1.42918
C	0.32250	1.31725	3.02980
H	0.92912	2.10760	2.55852
N	1.59243	-2.47906	-0.12640
C	2.16939	-3.02556	1.11639
C	1.97301	-3.11950	-1.39862
C	3.70808	-3.24596	1.00080
C	3.51540	-3.31591	-1.50309
C	4.09742	-4.01463	-0.26791
H	4.09209	-3.76259	1.90945
H	3.77106	-3.87636	-2.43063
H	3.73088	-5.06428	-0.21126
H	1.59895	1.15701	4.78567
C	1.92641	-1.97125	2.22050
H	0.83601	-1.81866	2.40620
H	2.35408	-2.27711	3.19768
H	2.39663	-0.99711	1.95451
C	1.27193	-4.48609	-1.67350
H	0.17756	-4.38580	-1.52827
H	1.45312	-4.82946	-2.71583
H	1.62422	-5.29189	-1.00096
C	1.50323	-4.34305	1.62010
H	0.40163	-4.22302	1.65180
H	1.72389	-5.20770	0.96508
H	1.85508	-4.60988	2.64130
C	1.53104	-2.16598	-2.53007
H	2.02070	-1.17314	-2.42724
H	1.78960	-2.56163	-3.53487
H	0.42730	-2.01703	-2.51994
H	3.98393	-2.30965	-1.59757
H	5.20437	-4.08749	-0.34914
H	4.19683	-2.24730	0.97168

TS(A′-A′′)
SCF (BP86) Energy = -2556.29035679
Enthalpy 0K = -2555.333060
Enthalpy 298K = -2555.276203
Free Energy 298K = -2555.423713
Lowest Frequency = -29.4821 cm^{-1}
Second Frequency = 16.5599 cm^{-1}
SCF (BP86-D3^{BJ}) Energy =
-2556.56944973
SCF (C_6H_6) Energy = -2556.33845039
SCF ($C_6H_5CH_3$) Energy = -2556.33835222
SCF (BS2) Energy = -2558.39617026

Ca	0.67130	0.01399	0.69132
N	1.29798	-1.53410	-0.91593
N	1.33825	1.50214	-0.95224
C	1.33240	-1.32204	-2.24689
C	1.29962	-0.03944	-2.85151
H	1.28790	-0.05207	-3.95032
C	1.33611	1.26009	-2.27692
C	1.39743	-2.51647	-3.18386
H	0.55626	-3.21434	-2.98925
H	2.32551	-3.10126	-3.01927
H	1.36296	-2.20674	-4.24472
C	1.32652	2.42446	-3.25676
H	0.29837	2.84125	-3.33544
H	1.63796	2.10645	-4.26959
H	1.97511	3.25674	-2.92109
C	1.43513	-2.85200	-0.37553
C	2.73033	-3.39142	-0.08954
C	2.81322	-4.62120	0.59649
H	3.80559	-5.04259	0.82758
C	1.66323	-5.32481	0.98337
H	1.75344	-6.28180	1.52160
C	0.39841	-4.81438	0.65755
H	-0.50287	-5.38510	0.93257
C	0.25706	-3.59129	-0.02949
C	4.01968	-2.69230	-0.53434
H	3.72508	-1.75830	-1.05676
C	4.82093	-3.55878	-1.53205
H	5.19588	-4.48835	-1.05335
H	5.70219	-3.00247	-1.91587
H	4.20632	-3.86224	-2.40422
C	4.91747	-2.29180	0.65515
H	4.39248	-1.60845	1.35218
H	5.83256	-1.77233	0.30005
H	5.24491	-3.17866	1.23855
C	-1.12930	-3.05901	-0.39822
H	-0.98141	-2.30984	-1.20516
C	-2.07057	-4.14294	-0.95803
H	-1.61398	-4.67684	-1.81704
H	-3.01928	-3.68250	-1.30285
H	-2.33698	-4.90048	-0.19090
C	-1.80549	-2.32035	0.77721
H	-2.15835	-3.03570	1.55202
H	-2.65700	-1.66968	0.45233
H	-1.09623	-1.65130	1.32295
C	1.47007	2.83837	-0.45697
C	2.75983	3.32336	-0.06896
C	2.85313	4.61735	0.48626
H	3.83892	5.00963	0.78190
C	1.71529	5.41744	0.66743
H	1.81213	6.42635	1.09935

C	0.45424	4.92805	0.29718
H	-0.43532	5.56009	0.44516
C	0.30249	3.64353	-0.26401
C	4.01823	2.47198	-0.26733
H	3.68154	1.41144	-0.28119
C	5.04970	2.63041	0.86744
H	5.53576	3.62899	0.85211
H	5.85996	1.87915	0.76009
H	4.59510	2.50196	1.87174
C	4.69405	2.74757	-1.63015
H	4.01769	2.53184	-2.47973
H	5.59974	2.11650	-1.75457
H	5.00769	3.81076	-1.70695
C	-1.08444	3.09764	-0.61214
H	-0.94569	2.31883	-1.39204
C	-2.04004	4.15375	-1.19694
H	-2.31222	4.92953	-0.45029
H	-2.98523	3.67146	-1.51916
H	-1.59588	4.66828	-2.07438
C	-1.73774	2.39988	0.60229
H	-1.00994	1.75054	1.15054
H	-2.61632	1.74809	0.32529
H	-2.05297	3.14677	1.36407
C	0.65091	-1.13160	3.39106
C	-0.11700	0.03243	3.56972
H	-1.17058	-0.04537	3.88005
C	0.46397	1.31484	3.34490
H	-0.12775	2.22804	3.49917
C	1.82374	1.40441	2.95084
H	2.27691	2.39336	2.77953
C	2.58857	0.23853	2.76090
H	3.64414	0.32109	2.45828
C	2.00393	-1.04632	2.96852
H	2.59714	-1.96171	2.82981
N	-4.10245	0.21790	0.03768
C	-5.06410	0.18523	1.13741
C	-4.61036	0.04231	-1.32078
C	-6.03466	-1.03687	1.03376
C	-5.57478	-1.18332	-1.43312
C	-6.66812	-1.16208	-0.35745
H	-6.81823	-0.97706	1.82343
H	-6.02021	-1.23498	-2.45282
H	-7.36775	-0.31505	-0.53878
H	0.19998	-2.11964	3.57154
C	-4.25772	0.04891	2.45309
H	-3.57747	0.91843	2.57654
H	-4.92599	0.00516	3.34051
H	-3.64121	-0.87335	2.43674
C	-5.33125	1.30330	-1.90810
H	-4.71317	2.20689	-1.72678
H	-5.48492	1.20126	-3.00519
H	-6.32423	1.48292	-1.45273
C	-5.90998	1.49582	1.28717
H	-5.24155	2.38068	1.24924
H	-6.66494	1.61273	0.48590
H	-6.45460	1.51164	2.25732
C	-3.38842	-0.21523	-2.23421
H	-2.83106	-1.11215	-1.89410
H	-3.68728	-0.37294	-3.29323
H	-2.69076	0.64871	-2.19775
H	-4.96657	-2.10831	-1.30398
H	-7.29001	-2.08286	-0.41855
H	-5.44395	-1.95879	1.24039

A’’

SCF (BP86) Energy = -2556.29081560
Enthalpy 0K = -2555.333563
Enthalpy 298K = -2555.275754
Free Energy 298K = -2555.427206
Lowest Frequency = 12.7604 cm^{-1}
Second Frequency = 18.9236 cm^{-1}
SCF (BP86-D3^{BJ}) Energy = -2556.56741564
SCF (C_6H_6) Energy = -2556.33797779
SCF ($C_6H_5CH_3$) Energy = -2556.33782442
SCF (BS2) Energy = -2558.39642795

Ca	-1.03194	-0.00175	0.71642
N	-1.38684	1.50317	-0.99399
N	-1.42778	-1.48294	-1.01570
C	-1.38319	1.30790	-2.32642
C	-1.35914	0.02713	-2.93317
H	-1.32538	0.03378	-4.03180
C	-1.38154	-1.26518	-2.34283
C	-1.38780	2.51355	-3.25032
H	-0.52814	3.18033	-3.02825
H	-2.30028	3.12704	-3.10100
H	-1.33743	2.21517	-4.31394
C	-1.29559	-2.44300	-3.30238
H	-0.23746	-2.77141	-3.40121
H	-1.65290	-2.16876	-4.31335
H	-1.86511	-3.32060	-2.93990
C	-1.45838	2.80661	-0.41567
C	-2.72756	3.41132	-0.14149
C	-2.76005	4.60009	0.61686
H	-3.73182	5.07066	0.84064
C	-1.58232	5.20191	1.08471
H	-1.63176	6.12775	1.67980
C	-0.34013	4.63467	0.76499
H	0.58347	5.13229	1.10105
C	-0.24772	3.44853	0.00680
C	-4.03635	2.82033	-0.67673
H	-3.77084	1.93439	-1.29032
C	-4.77921	3.81931	-1.59148
H	-5.13218	4.70779	-1.02602
H	-5.67064	3.34223	-2.05095
H	-4.12987	4.18763	-2.41230
C	-4.97001	2.33042	0.44990
H	-4.48829	1.54176	1.06304
H	-5.90591	1.90464	0.03008
H	-5.25701	3.15872	1.13253
C	1.11929	2.86826	-0.37006
H	0.95444	2.17691	-1.22354
C	2.11411	3.94724	-0.84317
H	1.68966	4.56230	-1.66354
H	3.04535	3.47136	-1.21281
H	2.40608	4.63428	-0.02102
C	1.74795	2.03347	0.76533
H	2.01493	2.67633	1.63237
H	2.65379	1.45609	0.43847
H	1.04233	1.27294	1.18063
C	-1.48450	-2.80559	-0.47827
C	-2.74899	-3.35067	-0.08118
C	-2.77221	-4.61777	0.53868
H	-3.73653	-5.05561	0.84081
C	-1.58997	-5.33451	0.77661
H	-1.63117	-6.32350	1.26036
C	-0.35604	-4.78838	0.39529

H	0.56766	-5.35665	0.58647
C	-0.27252	-3.52802	-0.23237
C	-4.05356	-2.59179	-0.34647
H	-3.78866	-1.51111	-0.38813
C	-5.11226	-2.78113	0.75740
H	-5.52973	-3.81033	0.76145
H	-5.96630	-2.09073	0.59525
H	-4.70334	-2.58764	1.77077
C	-4.66047	-2.95390	-1.72146
H	-3.96697	-2.72458	-2.55329
H	-5.59826	-2.38576	-1.89949
H	-4.90393	-4.03684	-1.77072
C	1.08854	-2.92979	-0.60071
H	0.91763	-2.18562	-1.40680
C	2.08641	-3.96873	-1.14675
H	2.40057	-4.69587	-0.36836
H	3.00603	-3.46043	-1.50087
H	1.65749	-4.54348	-1.99384
C	1.71414	-2.16444	0.58592
H	1.00255	-1.42245	1.02708
H	2.63684	-1.58262	0.30580
H	1.95678	-2.85837	1.42040
C	-0.95209	1.19769	3.36062
C	-0.14558	0.05645	3.51869
H	0.91847	0.17102	3.78022
C	-0.68388	-1.24251	3.32719
H	-0.05009	-2.13180	3.45142
C	-2.06668	-1.38621	3.00468
H	-2.49410	-2.39123	2.86811
C	-2.87741	-0.24505	2.86121
H	-3.94677	-0.36389	2.62265
C	-2.32986	1.05667	3.01325
H	-2.96330	1.94807	2.90018
N	4.30990	-0.15949	0.05308
C	5.23987	-0.16320	1.18005
C	4.86271	-0.02152	-1.29191
C	6.27076	1.01011	1.09298
C	5.88609	1.15737	-1.38477
C	6.94803	1.09239	-0.28033
H	7.02689	0.92006	1.90600
H	6.35895	1.18014	-2.39301
H	7.61120	0.21164	-0.43624
H	-0.52383	2.20170	3.49988
C	4.40520	0.02755	2.47030
H	3.67250	-0.80006	2.57993
H	5.05021	0.04058	3.37563
H	3.84323	0.98296	2.43116
C	5.53984	-1.32092	-1.84530
H	4.87726	-2.19336	-1.66907
H	5.72307	-1.23827	-2.93935
H	6.51276	-1.53941	-1.36440
C	6.01487	-1.51233	1.36121
H	5.30479	-2.36331	1.30837
H	6.78759	-1.67200	0.58467
H	6.52724	-1.54665	2.34819
C	3.68070	0.28468	-2.24174
H	3.15826	1.21033	-1.92476
H	4.01891	0.41827	-3.29220
H	2.94118	-0.54376	-2.21828
H	5.31785	2.10988	-1.27679
H	7.61431	1.98224	-0.33047
H	5.71846	1.96053	1.27453
